# Supplementary material for: Trends in the prevalence, incidence and surgical management of carpal tunnel syndrome between 1993 and 2013: an observational analysis of UK primary care records
Source: BMJ Open. 2018 Jun 19;8(6):e020166. doi: 10.1136/bmjopen-2017-020166 (PMC6020969; doi:10.1136/bmjopen-2017-020166)
Supplement: Supplementary file 5 [file bmjopen-2017-020166supp005.pdf]

Suppl. Table 1 The age and sex standardised estimates of the annual prevalence and incidence of CTS

Year	Age sex standardised prevalence (per 10,000 person years, 95% CI)	Age sex standardised incidence (per 10,000 person years, 95% CI)
1993	26.27 (26.13 – 26.42)	19.95 (19.83 – 20.07)
1994	26.83 (26.69 – 26.98)	20.46 (20.34 – 20.59)
1995	25.90 (25.77 – 26.05)	19.20 (19.08 – 19.33)
1996	25.64 (25.50 – 25.78)	19.61 (19.49 – 19.74)
1997	24.64 (24.20 – 25.07)	19.42 (19.30 – 19.55)
1998	25.42 (25.88 – 25.56)	20.05 (19.93 – 20.18)
1999	24.57 (24.44 – 24.71)	19.51 (19.39 – 19.64)
2000	24.77 (24.63 – 24.91)	19.73 (19.61 – 19.86)
2001	26.22 (26.08 – 26.36)	20.75 (20.63 – 20.88)
2002	28.22 (28.07 – 28.37)	22.22 (22.10 – 22.36)
2003	30.81 (30.65 – 30.96)	24.28 (24.15 – 24.42)
2004	33.51 (33.35 – 33.67)	27.00 (26.86 – 27.14)
2005	32.98 (32.82 – 33.14)	24.56 (24.42 – 24.70)
2006	32.55 (32.39 – 32.70)	24.14 (24.00 – 24.27)
2007	33.48 (33.32 – 33.64)	25.52 (25.38 – 25.66)
2008	35.59 (35.43 – 25.76)	27.07 (26.92 – 27.21)
2009	36.81 (36.64 – 36.98)	28.19 (28.05 – 28.34)
2010	36.40 (36.24 – 36.66)	27.53 (27.39 – 27.68)
2011	35.28 (35.12 – 35.44)	26.59 (26.45 – 26.74)
2012	35.50 (35.34 – 35.67)	26.75 (26.61 – 26.89)
2013	35.45 (35.29 – 35.61)	26.34 (26.01 – 26.49)
