# Supplementary material for: Trends in the prevalence, incidence and surgical management of carpal tunnel syndrome between 1993 and 2013: an observational analysis of UK primary care records
Source: BMJ Open. 2018 Jun 19;8(6):e020166. doi: 10.1136/bmjopen-2017-020166 (PMC6020969; doi:10.1136/bmjopen-2017-020166)
Supplement: Supplementary file 6 [file bmjopen-2017-020166supp006.pdf]

Suppl. Table 2. Demographics of the crude prevalent population presenting with CTS in each calendar year

Year	Female median age (25% - 75% Interquartile range)	Male median age (25% - 75% Interquartile range)
**1993**	49 (38 – 62)	53 (42 – 66)
**1994**	49 (39 – 62)	53 (42 – 66)
**1995**	50 (39 – 62)	52 (41 – 64)
**1996**	50 (40 – 62)	53 (41 – 66)
**1997**	51 (40 – 62)	53 (42 – 67)
**1998**	51 (40 – 62)	54 (43 – 67)
**1999**	51 (40 – 62)	54 (44 – 66)
**2000**	52 (41 – 64)	55 (44 – 67)
**2001**	53 (42 – 65)	55 (44 – 68)
**2002**	53 (41 – 64)	55 (44 – 67)
**2003**	54 (42 – 65)	55 (44 – 68)
**2004**	55 (43 – 65)	56 (45 – 68)
**2005**	54 (43 – 65)	58 (45 – 70)
**2006**	54 (43 – 66)	58 (45 – 70)
**2007**	54 (42 – 66)	54 (42 – 66)
**2008**	54 (43 – 66)	58 (46 – 70)
**2009**	54 (43 – 67)	58 (47 – 70)
**2010**	54 (43 – 67)	57 (46 – 71)
**2011**	54 (43 – 67)	58 (47 – 71)
**2012**	54 (43 – 67)	59 (48 – 71)
