# Supplementary material for: Trends in the prevalence, incidence and surgical management of carpal tunnel syndrome between 1993 and 2013: an observational analysis of UK primary care records
Source: BMJ Open. 2018 Jun 19;8(6):e020166. doi: 10.1136/bmjopen-2017-020166 (PMC6020969; doi:10.1136/bmjopen-2017-020166)
Supplement: Supplementary file 7 [file bmjopen-2017-020166supp007.pdf]

Suppl. Table 3. Demographics of the crude incident population presenting with CTS in each calendar year

Year	Female median age (25% - 75% Interquartile range)	Male median age (25% - 75% Interquartile range)
1993	50 (39 – 63)	51 (42 – 65)
1994	50 (40 – 63)	53 (43 – 66)
1995	51 (40 – 63)	53 (42 – 64)
1996	51 (40 – 64)	52 (41 – 65)
1997	51 (40 – 64)	55 (45 – 67)
1998	51 (40 – 63)	54 (44 – 68)
1999	52 (41 – 64)	55 (45 – 67)
2000	53 (42 – 65)	55 (44 – 68)
2001	53 (42 – 66)	55 (45 – 68)
2002	54 (42 – 66)	55 (44 – 67)
2003	55 (43 – 66)	56 (45 – 68)
2004	55 (44 – 66)	57 (45 – 68)
2005	55 (43 – 66)	58 (46 – 70)
2006	55 (44 – 67)	58 (46 – 70)
2007	54 (43 – 66)	58 (47 – 70)
2008	55 (44 – 67)	58 (47 – 70)
2009	55 (44 – 67)	59 (47 – 71)
