# Supplementary material for: Trends in the prevalence, incidence and surgical management of carpal tunnel syndrome between 1993 and 2013: an observational analysis of UK primary care records
Source: BMJ Open. 2018 Jun 19;8(6):e020166. doi: 10.1136/bmjopen-2017-020166 (PMC6020969; doi:10.1136/bmjopen-2017-020166)
Supplement: Supplementary file 9 [file bmjopen-2017-020166supp009.pdf]

Suppl. Table 5 The crude incidence of CTS by age and gender

Incidence by age and gender	1993	1994	1995	1996	1997	1998	1999	2000	2001	2002	2003	2004	2005	2006	2007	2008	2009	2010	2011	2012	2013
Female 18-29	12.69	12.69	12.84	12.88	11.45	12.32	11.77	10.14	8.63	10.23	11.54	12.26	10.70	10.48	11.61	9.96	13.90	12.19	11.55	11.89	10.68
Female 30-39	31.40	28.58	28.00	27.62	28.09	30.09	27.83	25.88	27.18	28.31	30.31	33.31	30.53	30.06	32.17	33.13	34.97	33.96	32.51	31.72	28.69
Female 40-49	38.43	37.75	33.08	36.31	35.17	37.42	31.74	34.72	34.05	34.94	35.63	37.37	36.27	35.62	40.06	42.51	40.89	40.86	40.05	42.06	39.50
Female 50-59	39.86	41.02	41.93	40.82	41.44	43.24	44.70	42.33	43.94	46.44	54.52	61.11	54.56	52.25	58.55	54.70	56.14	54.10	51.62	50.07	50.97
Female 60-69	25.54	29.79	24.64	25.70	25.08	26.41	26.43	26.86	30.23	32.54	39.12	46.20	38.44	36.30	31.00	42.47	42.24	41.87	37.74	35.80	37.32
Female 70+	24.45	22.29	24.21	25.28	26.17	25.09	25.25	27.67	29.29	32.87	34.96	39.57	37.78	39.60	39.13	41.89	46.03	44.89	44.46	42.29	43.53
Male18-29	3.58	3.69	3.09	3.02	2.32	2.29	2.35	1.44	2.24	2.75	2.60	3.14	2.60	2.58	3.33	3.13	2.77	3.00	3.56	3.51	3.35
Male 30-39	9.09	8.63	8.63	10.67	7.82	8.45	5.92	8.74	8.60	10.01	10.72	11.28	9.76	8.80	9.53	9.16	9.90	10.30	9.19	9.67	9.72
Male 40-49	14.23	14.35	12.63	11.83	11.86	12.41	13.43	13.26	14.39	16.32	16.08	17.78	14.28	14.25	16.36	18.23	17.76	19.58	17.15	18.13	17.63
Male 50-59	13.90	17.40	17.11	15.36	16.26	15.17	16.54	15.87	18.00	18.59	19.87	21.36	20.23	20.02	22.30	23.69	23.38	23.91	23.51	24.40	25.45
Male 60-69	13.62	15.14	11.42	12.22	13.02	13.48	15.58	15.96	18.78	18.49	19.80	23.71	21.12	21.81	23.95	24.79	27.56	23.24	24.59	27.90	26.23

Male 70+	14.88	18.38	15.72	16.64	18.34	18.06	17.29	19.27	20.53	21.95	24.71	27.24	27.92	26.94	29.67	31.84	33.81	32.52	33.23	33.78	33.83
