# Supplementary figures and images for: Trends in the prevalence, incidence and surgical management of carpal tunnel syndrome between 1993 and 2013: an observational analysis of UK primary care records
Source: BMJ Open. 2018 Jun 19;8(6):e020166. doi: 10.1136/bmjopen-2017-020166 (PMC6020969; doi:10.1136/bmjopen-2017-020166)

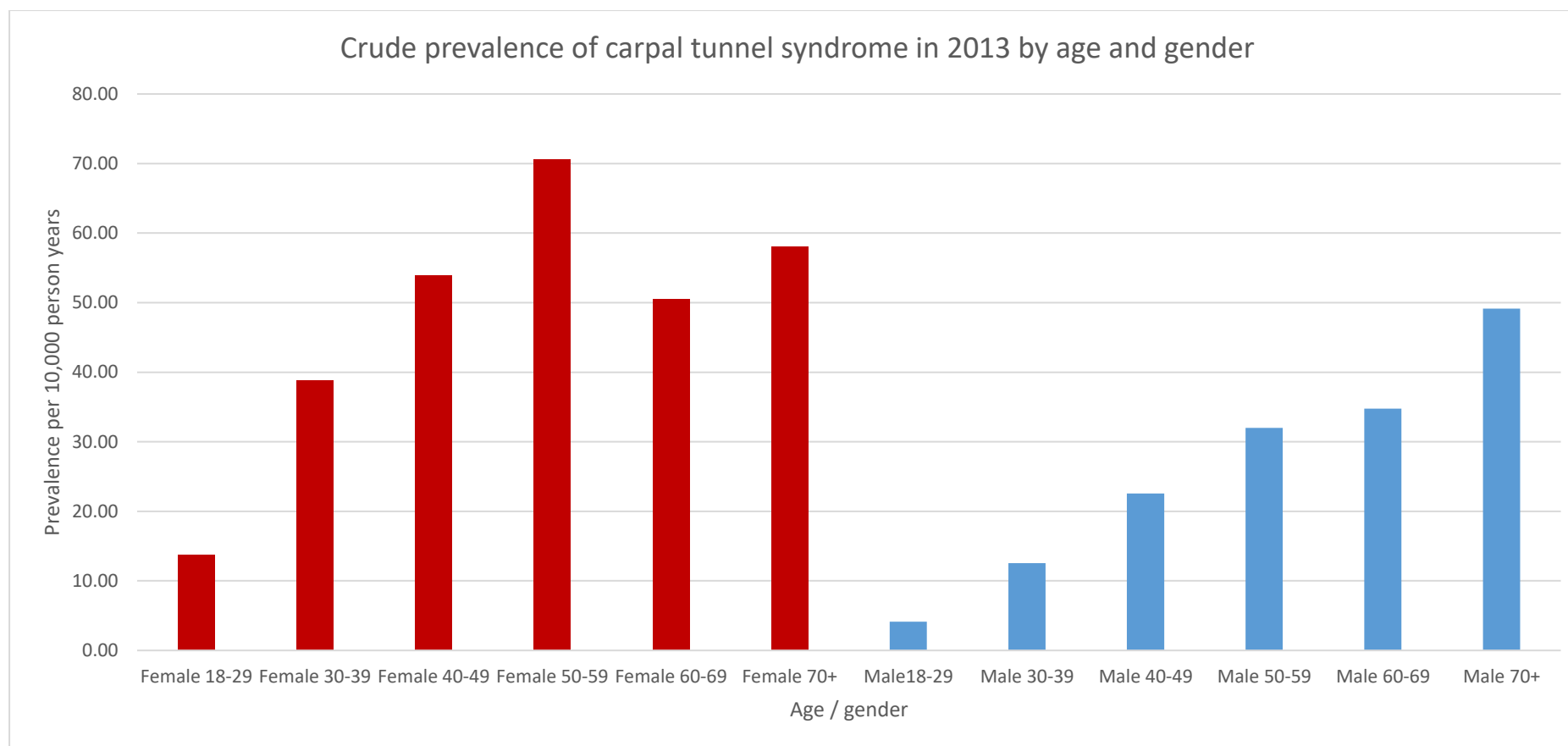

Suppl. Fig 2 Crude prevalence of carpal tunnel syndrome in 2013 by age and gender

Supplement: Supplementary file 2 [file bmjopen-2017-020166supp002.pdf]

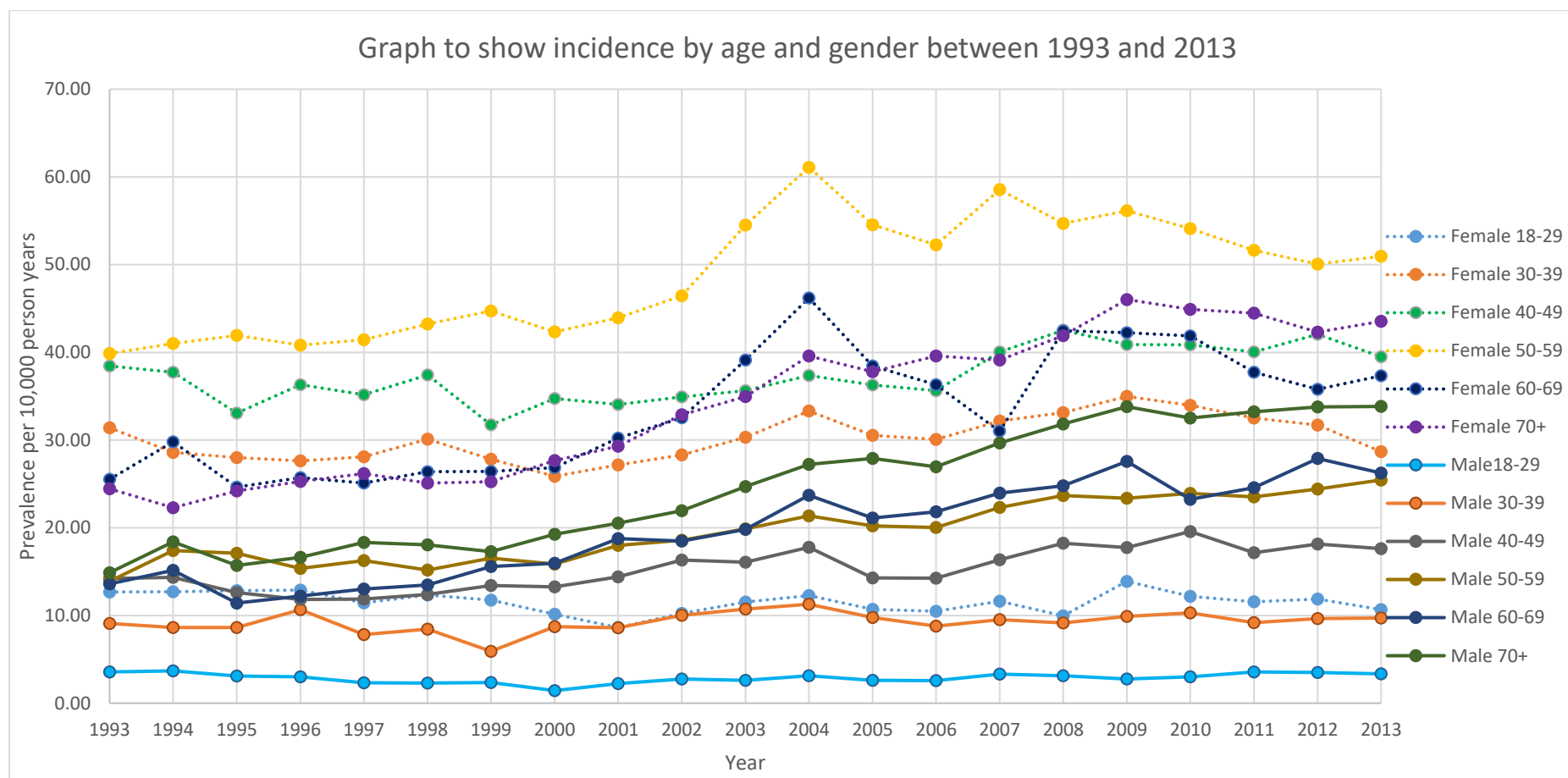

Suppl. Fig 3 Graph to show incidence by age and gender between 1993 and 2013

Supplement: Supplementary file 3 [file bmjopen-2017-020166supp003.pdf]

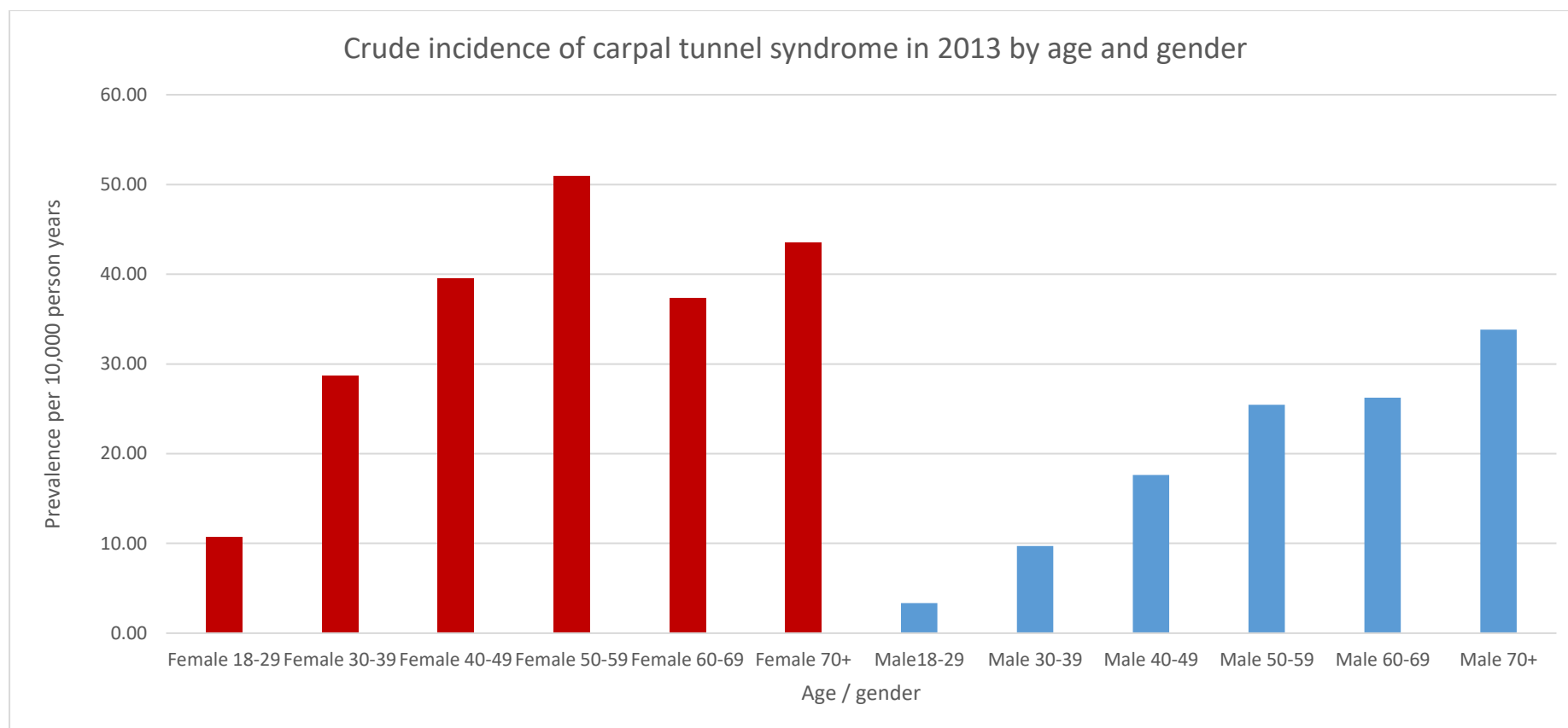

Suppl. Fig 4 Crude incidence of carpal tunnel syndrome in 2013 by age and gender

Supplement: Supplementary file 4 [file bmjopen-2017-020166supp004.pdf]
