# Supplementary material for: Trends in the prevalence, incidence and surgical management of carpal tunnel syndrome between 1993 and 2013: an observational analysis of UK primary care records
Source: BMJ Open. 2018 Jun 19;8(6):e020166. doi: 10.1136/bmjopen-2017-020166 (PMC6020969; doi:10.1136/bmjopen-2017-020166)
Supplement: Supplementary file 10 [file bmjopen-2017-020166supp010.pdf]

Suppl. Table 6 Comparison of population studies reporting the prevalence and / or incidence of carpal tunnel syndrome

Study Identifier	Study method	Definition of CTS	Comments
De Krom et al. 1992	Survey of a random age sex stratified sample of the general population taken from the population register of Maastricht between 1983 and 1985	Questionnaire based on symptoms and signs	[1]
Ferry et al. 1998	i) Cross sectional survey to estimate the point prevalence of hand symptoms (from a random sample of 1000 individuals from the UK general population, aged 18 to 75 years) and<br>ii) nerve conduction testing of a weighted sample<br>- Circa. 1998 (not stated)<br>- point prevalence determined	Based on nerve conduction studies using defined cut offs	Subjects over 54yrs had a higher prevalence than younger participants. No difference between genders was noted.[2]
Nordstrom et al. 1998	Prospective study conducted in the general population of the Marshfield Epidemiologic Study Area, Wisconsin, between 1991 and 1993	1. any diagnosis of possible, probable or definite CTS;<br>2. any diagnosis of probable or definite CTS; and<br>3. any diagnosis of possible , probable or definite CTS plus at least one of six clinical signs	A 3.5 fold increase in CTS incidence was noted compared with data from 20 years previously in the same study population[3]
Atroshi et al. 2000	Survey of a random sample of the age sex stratified general population of Southern Sweden, in 1997	Diagnosis based on clinical examination and positive electrophysiological findings	The population prevalence of symptoms was 14.4%; the prevalence of clinically and electrophysiologically confirmed CTS was 2.7% [4]
Papanicolaou, McCable & Firrell 2001	Cross-sectional study to evaluate prevalence of carpal tunnel syndrome in the General population of the United States	Katz hand diagram	After correcting for nonresponders the lowest possible estimate of CTS was 3.72% [5]

Mondelli, Giannini & Giacchi 2002	Prospective study of patients referred to four electrodiagnostic laboratories in the Siena area, Italy. Mean annual incidence calculated from time period 1991 to 1998	Diagnosis based on clinical history and electrodiagnostic evidence of a reduced distal conduction velocity of the median nerve (American Academy of Neurology standards)	Of the patients presenting 79.7% were women. The mean age at diagnosis was 55.0 +/- 14.4 years (range 16 to 97) [6]
Bland, Rudolfer 2003	Prospective collection of neurophysiological and clinical data of patients referred to two electromyography clinics in the UK between 1991 to 1993 and 1992 to 2001	Based on nerve conduction studies using defined cut offs	An increase in diagnosed cases was observed between the two data collection periods; attributed to referral of milder cases. Median nerve impairment was more severe in the elderly and men at all ages. [7]
Latinovic, Gulliford & Hughes 2006	Population study based in a general practice database of consulting primary care patients from 253 practices between January 1992 and 31 December 2000.	Read and Oxmis codes for carpal tunnel syndrome	Most frequent in women aged 45-54. In 2000 operative treatment was undertaken for 31% of incident CTS presentations [8]
Bonger et al. 2007	Analysis of the first and second Dutch National Survey of General Practice, conducted in 1987 and 2001	(International Classification of Primary Care) ICPC coded diagnosis	A crude increase in incidence over time was not statistically significant after subdividing by age and sex. Incidence rates were related to the job level in women, but not men [9]
Dieleman et al. 2008	Population study based in a general practice database (Integrated Primary Care Information (IPCI) database): data of consulting primary care patients in the Netherlands between 1996 and 2003	ICPC coded diagnosis	Neuropathic pain was noted to affect almost 1% of the population. Mononeuropathies and carpal tunnel syndrome were the most common causes [10]
Gelfman et al. 2009	Analysis of medical records linkage system 1981-1985 to 2000-2005 of residents of Olmsted County, Minnesota (Rochester Epidemiology Project)	Clinical coding with a sample verified by full record review	An increase in incidence was observed over the study period. An increase in young individuals seeking care for less severe CTS in the mid-1980's was followed in the 1990's by an increasing incidence in older people [11]

Atroshi et al. 2011	Analysis of the Skane Health Care Register (SCHR) (inhabitants presenting to public health providers), incident cases identified between 2006 - 2008	Physician diagnosed	[12]
Jenkins et al. 2012b	Prospective audit of patients referred to a regional hand service based in secondary care in Scotland between November 2004 and May 2010	Symptoms of pain or paraesthesia in the median nerve distribution and one or more of: nerve conduction deficit, thenar muscle wasting or positive Tinel or Phalen signs	Mean age of presentation 55.1years (range 22 to 96, SD 13.5 years).<br>Mean body mass index at presentation 29.5 kg/m2<br>CTS more common in: females (OR 1.9, 95% CI 1.5 to 2.5)<br>Incidence varied significantly between deprivation groups: most deprived 81/100,000 and least deprived 62/100,000 (OR 1.3, 95% CI 1.1 to 1.6) [13]
Jenkins et al. 2013	Prospective audit of patients referred to a regional hand service based in secondary care in Scotland between November 2004 and May 2010, who were employed	Clinical diagnosis based on history and examination, in most cases substantiated by nerve conduction studies	The greatest incidence as in caring and leisure occupations (197 per 100,000) and the lowest incidence was in the associate professional group (37 per 100,000) [14]
Dale 2013	Pooled analysis of six prospective studies collecting data from >50 workplaces, over variable time frames	A pooled case definition was derived to include clinical and elctrodiagnostic criteria	7.8% of 4321 subjects studied had prevalent CTS, with an additional 204 subjects meeting the CTS criteria, leading to an incidence of 2.3 cases per 100 person years [15]

1 de Krom MC, Knipschild PG, Kester AD, et al. Carpal tunnel syndrome: prevalence in the general population. *J Clin Epidemiol* 1992;45:373-6.

2 Ferry S, Pritchard T, Keenan J, et al. Estimating the prevalence of delayed median nerve conduction in the general population. *Br J Rheumatol* 1998;37:630-5.

3 Nordstrom DL, DeStefano F, Vierkant RA, et al. Incidence of diagnosed carpal tunnel syndrome in a general population. *Epidemiology* 1998;9:342-5.

4 Atroshi I. Carpal tunnel syndrome--prevalence, electrodiagnosis and outcome instruments. *Acta Orthop Scand* 1999;70:70-1.

5 Papanicolaou GD, McCabe SJ, Firrell J. The prevalence and characteristics of nerve compression symptoms in the general population. *Journal of Hand Surgery - American Volume* 2001;26:460-6.

6 Mondelli M, Giannini F, Giacchi M. Carpal tunnel syndrome incidence in a general population. *Neurology* 2002;58:289-94.

7 Bland JDP, Rudolfer SM. Clinical surveillance of carpal tunnel syndrome in two areas of the United Kingdom, 1991–2001. *Journal of Neurology, Neurosurgery & Psychiatry* 2003;74:1674-9 doi:10.1136/jnnp.74.12.1674.

8 Latinovic R, Gulliford MC, Hughes RA. Incidence of common compressive neuropathies in primary care. *J Neurol Neurosurg Psychiatry* 2006;77:263-5 doi:10.1136/jnnp.2005.066696.

9 Bongers FJ, Schellevis FG, van den Bosch WJ, et al. Carpal tunnel syndrome in general practice (1987 and 2001): incidence and the role of occupational and non-occupational factors. *Br J Gen Pract* 2007;57:36-9.

10 Dieleman JP, Kerklaan J, Huygen FJ, et al. Incidence rates and treatment of neuropathic pain conditions in the general population. *Pain* 2008;137:681-8 doi:10.1016/j.pain.2008.03.002 [doi].

11 Gelfman R, Melton LJ,3rd, Yawn BP, et al. Long-term trends in carpal tunnel syndrome. *Neurology* 2009;72:33-41 doi:10.1212/01.wnl.0000338533.88960.b9; 10.1212/01.wnl.0000338533.88960.b9.

12 Atroshi I, Englund M, Turkiewicz A, et al. Incidence of physician-diagnosed carpal tunnel syndrome in the general population. *Arch Intern Med* 2011;171:943-5.

13 Jenkins PJ, Watts AC, Duckworth AD, et al. Socioeconomic deprivation and the epidemiology of carpal tunnel syndrome. *Journal of Hand Surgery-European Volume* 2012;37E:123-9 doi:10.1177/1753193411419952.

14 Jenkins PJ, Srikantharajah D, Duckworth AD, et al. Carpal tunnel syndrome: the association with occupation at a population level. *Journal of Hand Surgery-European Volume* 2013;38E:67-72 doi:10.1177/1753193412455790.

15 Dale AM, Harris-Adamson C, Rempel D, et al. Prevalence and incidence of carpal tunnel syndrome in US working populations: Pooled analysis of six prospective studies. *Scandinavian Journal of Work, Environment and Health* 2013;39:495-505.
