# Supplementary material for: Trends in the prevalence, incidence and surgical management of carpal tunnel syndrome between 1993 and 2013: an observational analysis of UK primary care records
Source: BMJ Open. 2018 Jun 19;8(6):e020166. doi: 10.1136/bmjopen-2017-020166 (PMC6020969; doi:10.1136/bmjopen-2017-020166)
Supplement: Supplementary file 11 [file bmjopen-2017-020166supp011.pdf]

Suppl. Table 7 summary of reported prevalence and incidence by gender

Study Identifier	Country of Origin Data collection (Prevalence or Incidence)	Prevalence or Incidence per 100,000, per annum			Female / male ratio
		All	Female	Male	
De Krom et al. 1992	The Netherlands 1983 - July 1985 (Prevalence)	5700	5800	600	9.66
Atroshi et al. 2000	Sweden 1997 (Prevalence)	3800	4600	2800	1.64
Papanicolaou, McCable & Firrell 2001	United States 2001 (Prevalence)	3720			4.8
Ferry et al. 1998	United Kingdom Not stated (Incidence)	8200	6400	8200	0.78
Nordstrom et al. 1998	United States 1991 - 1993 (Incidence)	346	373	318	1.17
Mondelli, Giannini & Giacchi 2002	Italy 1991 – 1998 (mean) (Incidence)	276	506	139	3.64
Bland, Rudolfer 2003	Kent, UK 1991 - 2001 (Incidence)	105	120.5	60	2
	Huddersfield, UK		61.5	30	2

Latinovic, Gulliford & Hughes 2006	United Kingdom (Incidence)		192.8	87.8	2.23
Bongers et al. 2007	The Netherlands (Incidence) 1987	130	190	60	3.17
	2001	180	280	90	3.11
Dieleman et al. 2008	The Netherlands 1996 - 2003 (Incidence)	233.1			
Gelfman et al. 2009	United States (Incidence) 1981-1985	258	337	177	1.90
	2001-2005	424	542	303	1.79
Atroshi et al. 2011	Sweden 2006 - 2008 (Incidence)		428	182	2.35
Jenkins et al. 2012b	Scotland 2004 - 2010 (Incidence)	72	98	43	2.28
Jenkins et al. 2013	Scotland 2004 - 2010 (Incidence)	103			
Dale 2013	United States (Incidence)	2300			
