# Supplementary material for: Trends in the prevalence, incidence and surgical management of carpal tunnel syndrome between 1993 and 2013: an observational analysis of UK primary care records
Source: BMJ Open. 2018 Jun 19;8(6):e020166. doi: 10.1136/bmjopen-2017-020166 (PMC6020969; doi:10.1136/bmjopen-2017-020166)
Supplement: Author's manuscript [file bmjopen-2017-020166.draft_revisions.pdf]

BMJ Open

BMJ Open is committed to open peer review. As part of this commitment we make the peer review history of every article we publish publicly available.

When an article is published we post the peer reviewers' comments and the authors' responses online. We also post the versions of the paper that were used during peer review. These are the versions that the peer review comments apply to.

The versions of the paper that follow are the versions that were submitted during the peer review process. They are not the versions of record or the final published versions. They should not be cited or distributed as the published version of this manuscript.

BMJ Open is an open access journal and the full, final, typeset and author-corrected version of record of the manuscript is available on our site with no access controls, subscription charges or pay-per-view fees (http://bmjopen.bmj.com).

If you have any questions on BMJ Open's open peer review process please email info.bmjopen@bmj.com

BMJ Open

Trends in the prevalence, incidence and surgical management of carpal tunnel syndrome between 1993 and 2013: an observational analysis of UK primary care records

Journal:	*BMJ Open*
Manuscript ID	bmjopen-2017-020166
Article Type:	Research
Date Submitted by the Author:	18-Oct-2017
Complete List of Authors:	Burton, Claire; Keele University, Arthritis Research UK Primary Care Centre, Research Institute for Primary Care & Health Sciences<br>Chen, Ying; Keele University, Arthritis Research UK Primary Care Centre, Research Institute for Primary Care & Health Sciences<br>Chesterton, Linda; Keele University, Arthritis Research UK Primary Care Centre, Research Institute for Primary Care & Health Sciences<br>van der Windt, Danielle; Keele University, Arthritis Research UK Primary Care Centre, Research Institute for Primary Care & Health Sciences
Keywords:	PRIMARY CARE, SURGERY, Musculoskeletal disorders < ORTHOPAEDIC & TRAUMA SURGERY, Hand & wrist < ORTHOPAEDIC & TRAUMA SURGERY

SCHOLARONE™
Manuscripts

For peer review only

Trends in the prevalence, incidence and surgical management of carpal tunnel syndrome between 1993 and 2013: an observational analysis of UK primary care records

Authors

Claire L. Burton[a] c.burton@keele.ac.uk GP Research Fellow

Ying Chen[a] Research[a] Research Associate in Biostatistics

Linda S. Chesterton[a] Senior Lecturer in Physiotherapy

Danielle A. van der Windt[a] Professor of Primary Care Epidemiology

[a] Arthritis Research UK Primary Care Centre, Research Institute for Primary Care & Health Sciences, Keele University, Staffordshire, England. ST5 5BG

Word count (excluding title page, abstract, references, figures and tables) 4266

For peer review only

Abstract

Objectives

To describe the prevalence, incidence and surgical management of carpal tunnel syndrome (CTS), between 1993 and 2013, as recorded in the Clinical Practice Research Datalink (CPRD)

Design

We completed a series of cross-sectional epidemiological analyses to observe trends over time.

Setting

Primary care data collected between 1993 and 2013, stored in the Clinical Practice Research Datalink

Population

Individuals ≥ 18 years were selected. Prevalent and incident episodes of Carpal Tunnel syndrome (CTS) and episodes surgical intervention were identified using a list of pre-identified Read codes.

Analysis

We defined incident episodes as those with no preceding diagnostic code for CTS in the past 2 years of data. Episodes of surgery were expressed as a percentage of the prevalent population during the same calendar year. Joinpoint regression was used to determine significant changes in the underlying trend.

Results

The prevalence of CTS increased over the study period, with a particular incline between 2000 and 2004 (annual percentage change 7.81). The female to male prevalence ratio reduced over time from 2.74 in 1993 to 1.93 in 2013. The median age of females and males with CTS were noted to increase from 49 and 42 years respectively in 1993 to 54 and 48 years respectively in 2013. Incidence was also noted to increase over time. After an initial increase between 1993 and 2007, the percentage of prevalent patients with a coded surgical episode began to decrease after 2007 to 27.41% in 2013 (annual percentage change -1.7)

Conclusion

This study has demonstrated that the prevalence and incidence of carpal tunnel syndrome increased over the study period between 1993 and 2013. Rates of surgery for CTS also increased over the study period, however after 2007, the percent of patients receiving surgery showed a statistically significant decline back to the rate seen in 2004.

Key words

Carpal tunnel syndrome; primary care; epidemiology; incidence; prevalence; surgery

For peer review only - http://bmjopen.bmj.com/site/about/guidelines.xhtml

Strengths and Limitations

- Provides updated epidemiological data about a common and bothersome condition
- Set in primary care, where most cases of carpal tunnel syndrome present
- Utilises a large primary care database, generalizable to the UK population
- Relies on the correct coding and capture of episodes of carpal tunnel syndrome and carpal tunnel release surgery

For peer review only

INTRODUCTION

Carpal tunnel syndrome (CTS) is a chronic focal compressive neuropathy caused by the entrapment of the median nerve at the level of the carpal tunnel in the wrist.[1] CTS is the most common presentation of the entrapment neuropathies [2] and is characterised by symptoms including paraesthesia, dysthesia, sensory loss and eventually weakness and atrophy of the thenar muscle. Symptoms are usually localised to the hand but can spread proximally to the forearm, upper arm and even shoulder.[3] Despite causing relatively localised symptoms, CTS can have substantial physical, psychological and economic consequences.[4, 5]

The diagnosis of CTS is generally accepted to be a clinical one (based on history and examination findings) [6], although electrodiagnostic tests are commonly requested to confirm the diagnosis or differentiate among diagnoses, especially in the presence of thenar atrophy and / or persistent numbness or if surgical management is being considered.[7] The treatment of CTS is usually defined as either surgical or conservative (non-surgical). Local steroid injections and night splinting form the mainstay of primary care interventions in carpal tunnel syndrome, as indicated by national care pathways.[8, 9] Patients with moderate, severe or deteriorating symptoms following conservative treatment or sudden and severe symptoms should be referred for consideration of surgery.[10] Carpal tunnel release surgery (CTR) is routinely carried out under local anaesthetic as day surgery. Open and endoscopic approaches are used to release the flexor retinaculum. Adjuncts to the release include a tenosynovectomy, neurolysis of the median nerve or lengthening or reconstruction of the flexor retinaculum.[11] Previous studies have sought to estimate the prevalence and / or incidence of CTS. Such epidemiological studies have been diverse in their approach to the populations studied and case definitions applied.[12] The reported estimates for annual prevalence range from 3720-5700 per 100,000 per year [13-15] and the reported incidence from 72 – 8200 per 100,000 per year.[12, 16-22] CTS is generally accepted to be more common in women; the female to male ratio ranges between 0.78 and 9.66 [12, 13] A number of previous studies have observed the trends of prevalence or incidence over time and identified an increase [17, 18, 23], with 2005 being the latest data collection point. The most recent primary care based study in the UK utilised data from between 1992 and 2000.[16]

Episodes of CTR have also been shown to increase, with audit data from one major tertiary UK Hand Centre suggesting that referral for CTR increased over a 10 year period from 59.7 to 112 per 100,000 population per year between 1989-9 and 2000-1.[24] Using Hospital Episode Statistics (HES) between 1998 and 2011, Bebbington and Furniss also observed an increase in the absolute number of patients with CTS and episodes of CTR, however they also noted a decrease in the use of surgery post 2008.[25]

Previous studies have used a range of methods to classify episodes of CTS and have been conducted in a number of population settings. CTS is essentially a clinical diagnosis, and in the UK, the majority of patients will first present to and be managed within primary care. Only a proportion of these patients will be referred into more specialised services and since not all surgical episodes will take place in secondary care (hospitals), as community clinics are now receiving referrals, primary care records should capture the majority of episodes. Data from a high quality source, representative of the UK population is necessary to support the planning and commissioning of services.

The aim of this study is therefore to provide updated estimates of the prevalence, incidence and surgical management of carpal tunnel syndrome and describe trends over a 20 year

For peer review only - http://bmjopen.bmj.com/site/about/guidelines.xhtml

period, using data from a large national primary care database (Clinical Practice Research Datalink (CPRD).

METHODS

This was an observational study using the Clinical Practice Research Datalink (CPRD) to estimate the prevalence, incidence and surgical management of CTS from 1993 to 2013. CPRD is a live, primary care database of anonymised medical records from general practices. It holds information of over 11.3 million patients from 674 practices in the UK since 1987. 4.4 million active (alive and currently registered) patients are currently contributing information to the datalink, which equates to 6.9% of the UK population. [26] CPRD is broadly representative of the UK general population in terms of age, gender and ethnicity. [26] The CPRD has National Research Ethics Committee (NRES) approval for observational research using primary care data and as such no further permissions were required. The Independent Scientific Advisory Committee (ISAC) study protocol 14_167 was approved in September 2014. Patients were not directly involved in the design of this study, however the results will be used to inform discussions regarding further research in this field with our local Research User Group.

During clinical interactions, Read Codes are used to record signs and symptoms, treatments and therapies, investigations, occupations, diagnoses and appliances. Read Codes make up a hierarchical 'thesaurus' stored by the computer. Clinical information is hence stored in a retrievable and analysable format.[27]

The study population consisted of men and women over 18 years of age. Patients were required to have up to standard (practice level) research quality (patient level) data in CPRD, for two years prior to an incident episode and at the point of diagnosis for a prevalent episode. The 'up to standard' metric (defined by CPRD) is based on continuity of recording and the recording of deaths, and set at the latest date at which practices met the quality criteria. The acceptable patient metric is based on registration status, the patient record itself and valid age and gender.[26]

Prevalent and incident patients were identified by a consultation recorded using one of the Read codes listed in Table 1. Some treatment codes and in the case of in injections, linked prescription data, were included as evidence of diagnosis as per previous studies.[16] Pilot work using a local primary care database (Consultations in Primary Care Archive, CiPCA [28]) had noted that 30% of CTS cases with a treatment code (i.e. CTR or a coded carpal tunnel injection) had not initially received a diagnosis code. This means that at presentation, patients may have been attributed a more generic term such as 'hand pain' and later gone on to receive condition specific treatment. Hence, treatment codes were used to capture such cases, which would be missed when using diagnostic codes only.

Table 1 Readcodes used to define a prevalent or incident episode of carpal tunnel syndrome

The prevalence of individuals consulting with CTS was calculated per annum. The numerator for prevalence was the number of patients with a record of a CTS diagnosis or evidence of an episode of CTR or a carpal tunnel injection (CTI), in each calendar year. In order to determine annual incidence, the numerator was the number of patients with a record of CTS or evidence of CTR or CTI, without a prior record of these codes during a run-in-period of two years. This two year run-in period was based on expert consensus with the aim of estimating the period of time during which a new episode of CTS may develop. It was felt unlikely that a patient with ongoing bothersome symptoms would not have presented in primary care within this 2 year period. CTS could present as a new episode in the contralateral wrist sometime after the initial presentation, hence it was not felt possible to

For peer review only - http://bmjopen.bmj.com/site/about/guidelines.xhtml

define this criterion as 'no previous recorded episode'. All incidence patients were therefore required to have complete registration for the 2 calendar years prior to the event date. Pilot work in CiPCA had shown that over 9 years observed, 4% of potential incident cases were lost due to the lack of 2 years registration data required to define an incident episode.

The denominator population for calculation of prevalence was the total up-to-standard person-years contributed to CPRD by patients over the age of 18 years, for each annual period between 1993 and 2013. In order to apply the same criteria to both the numerator and denominator populations, the denominator populations for calculating incidence were also required to have registration at the mid-point of the year, two calendar years before the index year.

Episodes of carpal tunnel release (CTR) were identified using Read codes as shown in Table 2. In addition, codes used to define 're-release of carpal tunnel' and 'revision of carpal tunnel release' were included as a surgical episode (if first recorded). These terms were not included in the definition of CTS for the estimation of prevalence and incidence as they may not have indicated an episode of 'idiopathic' CTS but rather iatrogenic symptoms following previous (unsuccessful) surgery. Of note revision codes contributed 1.00% of the total surgical codes used. Results were expressed as the percentage of patients with a prevalent episode of CTS having a code of CTR in the same calendar year. Percentages were calculated based on the number of prevalent cases as opposed to incident cases as it was felt likely that patients would receive surgery in the annual period following their index consultation.

Table 2 Read codes used to define a surgical episode

Statistical methods

Age and sex specific annual prevalence and incidence were determined for each calendar year, between 1993 and 2013 and presented as n / 10,000 person years. For confidence interval calculation a Poisson distribution was used. As a sensitivity analysis, age and sex standardised annual figures of CTS prevalence and incidence for each year were also calculated, using population estimates provided by the website of the Office of National Statistics.[29] Un-standardised and standardised rates were very similar, hence we report un-standardised rates as the primary outcome.

Episodes of CTR were identified and the frequency in each calendar year expressed as a percentage of the prevalent population for the same time period. Emerging trends were described. Joinpoint regression was used to determine mean Annual Percentage Change (APC) and assess when significant changes ('joinpoints') occurred in the underlying trend. This method assists the exploration of the potential influence of changes in practice, although such potential associations cannot be proven.[30, 31] Models were fitted using the JOINPOINT REGRESSION PROGRAM (version 4.3.1.0) and the best fitting model chosen (up to 5 joinpoints).

RESULTS

Trends in prevalence

Table 3 presents the prevalence (crude estimates) of patients presenting in primary care with carpal tunnel syndrome between 1993 and 2013 and the demographics of the population. The denominator population for prevalence increased from 1,117,433 person years in 1993 to 3,473,094 person years in 2013. The total prevalence in 1993 was 26.03 per 10,000 person years (95% CI 25.10 – 27.00), and for 2013, 36.08 per 10,000 person years (95% CI 35.45 – 36.72). As shown in Figure 1, prevalence appeared to decrease

For peer review only - http://bmjopen.bmj.com/site/about/guidelines.xhtml

between 1993 and 2000 (annual percentage change APC = -0.84%, 95% confidence interval -2.6 to 1.0). It then increased between 2000 and 2004 (APC = 7.81%, 95% CI 3.1 – 12.7) and then increased at a slower rate between 2004 and 2013 (APC = 1.08%, 95% CI 0.4 – 1.8). The female to male ratio reduced over time from 2.74 in 1993 to 1.93 in 2013. The median age of female and male patients with CTS increased from 49 and 42 years respectively in 1993 to 54 and 48 years respectively in 2013 (see Supplementary Table 1).

Table 3 Crude prevalence of carpal tunnel syndrome (n/10,000 person years) per calendar year, as presented in UK primary care (CPRD)

Trends in incidence

Table 5 presents the annual incidence (crude estimates) for patients presenting in UK primary care with carpal tunnel syndrome between 1993 and 2013 and the demographics of the population. The denominator population for incidence, which is dependent on patients having 2 years up to standard data prior to the midpoint of the year in question, increased from 783,330 person years in 1993 to 3,015,670 person years in 2013. The crude incidence in 1993 was 20.22 per 10,000 person years (95% CI 19.24 - 21.24)), and for 2013, 27.09 per 10,000 person years (95% CI 28.28 – 35.95). As shown in Figure 2, the results of the best fitting Joinpoint regression suggest the incidence increased between 1993 and 2000 (APC = 0.3, 95% CI -2.3 – 2.9). It then increased more quickly between 2000 and 2004 (APC = 6.9, 95% CI 0.5 – 13.7), before slowing between 2004 and 2013 (APC = 0.7. 95% CI -0.2 – 1.6). The female to male ratio reduced over time from 2.57 in 1993 to 1.88 in 2013. The median age of female and male patients were noted to increase from 50 and 55 years respectively in 1993 to 51 and 59 years respectively in 2013 (see Supplementary Table 2). The age and sex standardised estimates of the annual prevalence and incidence of CTS are shown in Supplementary Table 3.

Table 4 Crude incidence of carpal tunnel syndrome (n/10,000 person years) per calendar

Figure 1 Joinpoint analysis of the crude prevalence of carpal tunnel syndrome between 1993 and 2013

year, as presented in UK primary care (CPRD)

Figure 2 Joinpoint analysis of crude incidence

Trends in the percentage of patients with carpal tunnel syndrome referred and receiving surgical management

Table 5 presents the percentage of prevalent patients with a recorded episode of CTR in each calendar year between 1993 and 2013 and the demographics of this sample. The percentage of all patients with a recorded episode of CTR in 1993 was 19.35%, and for 2013, 27.41%. As shown in Figure 3 the percentage of patients with a coded episode of CTR increased between 1993 - 2007 (annual percentage change APC = 2.6, 95% CI 1.9 – 3.2). It then appeared to decrease between 2007 and 2013 (APC = -1.7, 95% CI -3.3 - -0.3). The median age of females and males receiving CTR were noted to increase from 53 and 55 years respectively in 1993 to 57 and 62 years respectively in 2013.

Table 5 Percentage of patients with carpal tunnel syndrome with a recorded episode of carpal tunnel release surgery per calendar year, as presented in UK primary care (CPRD)

For peer review only - http://bmjopen.bmj.com/site/about/guidelines.xhtml

Figure 3 Joinpoint analysis of percentage of prevalent patients having carpal tunnel surgery

DISCUSSION

Whilst the prevalence and incidence of CTS have increased over the study period 1993-2013, results show that episodes of surgery, increased until 2007 and declined thereafter.

Supplementary Tables 4 and 5 summarise estimates of the prevalence, incidence and sex ratios of CTS from previous research, demonstrating the substantial variation in results between studies, which may partly be the results of differences in definition of CTS applied and population observed. Studies which also utilised primary care data showed a similar estimate of the incidence of CTS in a UK primary care population [16] and similarly reported an increase in incidence over time, albeit in a Dutch primary care population.[19]

The variability in the case definition of carpal tunnel syndrome was highlighted by Descatha et al 2011 who identified seven case definitions of CTS proposed for use in population based studies. Definitions included variations of: symptoms only; symptoms and examination findings; symptoms and either physical examination or electrodiagnostic results and symptoms and electrodiagnostic restults. This study showed a range in the population prevalence of CTS from 2.5% to 11%, with studies using less specific case definitions yielding higher prevalence rates.[32] Misclassification ranged between 1 and 10%.The prevalence of CTS in any given population is likely therefore to depend on the definition of CTS applied. The case definition in our study is derived from GP recorded diagnosis and treatment codes, which may have been based on clinical findings alone; those who have had further investigations and those who have received definitive condition specific treatment. Hence it utilises a pragmatic approach, across a large population that will include all patients presenting to their GP with symptoms. Our study methods do however assume that patients with symptoms will be presenting in primary care or be receiving definitive coded treatment. The study will not capture patients with chronic symptoms who are not presenting in primary care or who had a coded episode of surgery or injection.

Although Joinpoint analysis does not provide evidence for the cause of a change in observed outcomes, it highlights when a significant change in trend has taken place. Our results suggest that the annual percentage change in prevalence and incidence was highest between 2000 and 2004. A possible reason for this may be the publication of the UK Government's information technology strategy for the NHS in 1998,[33] which proposed that by 2005, the person-based electronic health record (HER), would have been fully implemented.[34] Although no direct evidence for this was found, it may be possible that with the increasing use of IT systems in primary care and attention to providing Read codes for each consultation, episodes of CTS were more frequently and accurately recorded. This would not however explain the continuing increase of the incidence in CTS post 2005.

Between 2000 and 2004, the Government implemented the second phase of its 'War on Waiting,' i.e. the reduction of waiting times. For example, the maximum wait for a day-case procedure (e.g. a CTR) was reduced from 18 months to 6 months. [35] The peak in prevalence of CTS (with our definition partly based also on treatment codes, which in 2013 constituted 29.36% of prevalent patients) observed in 2004 may therefore be partly explained by the fact that patients requiring surgery were 'accumulating' between 2000 and 2004 and subsequently received definitive treatment. This effect would however not be expected to impact so heavily upon the incidence, which disregards repeat patient presentations in subsequent annual periods, unless patients with a less specific code received treatment and appeared as an incident case. The introduction of the 18 week target

For peer review only - http://bmjopen.bmj.com/site/about/guidelines.xhtml

of time from referral to treatment in 2008 did not seem to have a similar impact on estimates of prevalence or incidence of CTS, which makes it less certain to what extent these policy changes may have influenced our results. There are likely to be further reasons behind the observed changes.

The change in trends of 2004 may also represent a change in service. The introduction of the Quality and Outcome Framework (QOF) occurred with the advent of the General Medical Services (GMS) contract in 2004. Although there has never been a musculoskeletal health domain, the importance of coding to maintain registers and evidence of outcomes in line with QOF may have influenced coding behaviour.

At the same time as QOF, Primary Care Trusts (PCT's) were given a role in commissioning services. The ability of PCT's to commission new services heralded the development of the Musculoskeletal Interface Clinics (MIC), which act as a 'one stop shop' for patients with musculoskeletal problems. A referral to this clinic from primary care may also be a reason prevalent patients with persisting symptoms stopped presenting in primary care.

These three factors (improved coding, service redevelopment and a reduction in waiting times) may all partly explain the change in incidence and prevalence of CTS between 2000 and 2004 but are unlikely to fully explain the observed trends. Further factors of potential influence may include the increasing rates of risk factors of CTS such as diabetes and obesity.[36, 37]

The joinpoint analysis suggested an increase in surgical management of CTS between 1989 and 2007 (APC = 2.74), followed by a reducing trend between 2007 (95% CI 2004-2009) and the end of the study in 2013 (APC = -1.83).

Previous studies have described the epidemiology and the rates of CTR in the UK. This study provides updated data observing the presenting primary care population. Using data from the General Practice Research Database (GPRD) (forerunner to CPRD) Latinovic et al reported that 31% of patients with CTS had surgery in 2000 [16], which is similar to the 25.5% found in our study at the same time point. The small difference between the estimates may be the result of a difference in the calculation used to derive the denominator population. Audit data from one tertiary hand centre, Wild et al also showed that the rate of referrals for CTR surgery had increased over the 10 years between 1989-9 and 2000-1.[24] Furthermore, Bebbington and Furniss observed demographic population shifts in hand conditions including CTS within Hospital Episode Statistics, which record diagnoses and procedures performed within NHS Hospitals in England. They used linear regression to predict future trends in hand surgery, showing that whilst absolute numbers of CTS diagnoses and CTR procedures increased between 1998 and 2011, the pre-2008 increase in CTR was significantly steeper than the post-2008 slope ($p < 0.001$).[25] This is suggestive of a decrease in the surgical management of CTS in terms of the proportion of patients with CTS having an operation, but not necessarily in the numbers of surgical episodes in absolute terms, which Bebbington and Furniss predict will have increased by 99% (95% CI 65 – 132) in 2030 compared to 2011.[25] The data from CPRD however, suggested a reduction in both real term episodes of CTR as well as the proportion of the (increasing) prevalent population receiving surgical treatment.

We may speculate regarding potential reasons for the initial increase in surgical management of CTS, for example, increased access to specialist services (e.g. community based Musculoskeletal Interface Clinics); increased litigation leading to more definitive treatments being sought, and increased patient expectations and demand, but we have no evidence for such explanations.

For peer review only - http://bmjopen.bmj.com/site/about/guidelines.xhtml

The decreasing trend in the use of the use of CTR post 2007 is likely to be multifactorial, however the changing structure of the NHS and its funding streams may have influenced the observed trend. Around 2007 – 2008, practice-based commissioning (PBC) was being introduced. This gave primary care notional budgets with which to purchase care for their patients with the aim of aligning clinical and financial responsibility. Restricting access to certain procedures including CTR, by implementing pre-specified criteria, was one way to help achieve this, which may have resulted in a reduction in the use of CTR.

There are a number of limitations associated with the data in this study. The accuracy of consultation data is dependent on the validity of the computerised information it uses. In a review of 212 publications which aimed to validate diagnoses recorded in GPRD data, Herrett et reported that the median proportion of cases with a confirmed diagnosis was 89% (range 24 – 100%), but the majority of publications did not present the sensitivity of a coded diagnosis, which means that information regarding the proportion of missed cases is lacking. Potential misclassification; non-attendance in primary care; variation in between GP coding and a lack of coding may all lead to an unmeasured shortfall in observed cases.[26, 38]. This study relies on the diagnosis of CTS to be correct and the subsequent coding to be precise. Whilst CTS diagnoses have not been validated, in a study comparing musculoskeletal diagnoses in four different databases, Jordan et al suggested that musculoskeletal coding in GPRD was less reliable than in its other healthcare datasets including CiPCA.[39] We took measures to reduce the effect of miscoding (e.g. including surgery and injection codes in prevalence measures, if diagnostic codes had not been used), but it is possible that results will not be entirely representative of the true prevalence and incidence of CTS.

Given the lack of clarity in the accuracy of coding and the likelihood that associated clinical encounters following a CTR were coded using a surgical code, only the first surgical code could reliably be used to indicate an episode of surgery. This is likely to have led to an underestimation of surgical episodes being identified as episodes on the contralateral hand will have been automatically discounted as they were undistinguishable. Furthermore, prevalence and incidence were similarly likely to have been underestimated as repeat presentations for the ipsilateral hand are indistinguishable from presentations in the contralateral hand.

Whilst CPRD provides a large generalizable sample, which has substantial benefits when estimating epidemiological trends, it cannot directly measure patient reported outcomes. Furthermore, surgery can be seen as a ‘gold standard’ treatment, but it does not necessarily signify cure. A review of the surgical treatment of CTS reported that 70% - 90% of patients undergoing a CTR have a good outcome (definitions varied).[40] In a retrospective cohort study over a mean follow up of 13 years post-surgery, 88% of patients were either completely satisfied or very satisfied with surgery. 74% reported their symptoms had completely resolved. 1.8% (113 patients) had undergone repeat surgery. [41] There is little evidence however that CTR is an appropriate initial management option for patients presenting to primary care with mild to moderate symptoms, especially in the absence of high quality trial evidence that conservative management is ineffective.[42, 43]

Future research in this field could describe the characteristics of patients presenting with CTS in greater detail, and observe course and prognosis of CTS in primary care. It may then be possible to identify predictors of the outcome of primary care management, and potentially identify patients requiring surgery.

CONCLUSION

For peer review only - http://bmjopen.bmj.com/site/about/guidelines.xhtml

An increase in the incidence and prevalence of CTS is likely to lead to an increased demand on services and cost to the healthcare economy.[25] This study has demonstrated an increase in the prevalence and incidence of carpal tunnel syndrome over the study period between 1993 and 2013. Rates of referral for CTS and surgical intervention have also increased over the study period, however in the later years of the study, the percent of patients receiving surgery has begun to decline.

Acknowledgments
The authors wish to thank Dr Dahai Yu for his assistance with the data download.

Declaration of competing interests and funding statement

All authors have completed the ICMJE uniform disclosure form at www.icmje.org/coi_disclosure.pdf and declare:

“CB is funded by the National Institute of Health Research School for Primary Care (NIHR SPCR) GP Career Progression Fellowship. The views expressed are those of the authors and not necessarily those of the NIHR, the NHS or the Department of health; DvdW is a member of PROGRESS Medical Research Council Prognosis Research Strategy (PROGRESS) Partnership (G0902393/99558); no other relationships or activities that could appear to have influenced the submitted work."

Details of contributors

CB, LC, YC and DwdW all contributed to the initial draft and subsequent revisions. CB is the guarantor of the paper. All authors had full access to all of the data and can take responsibility for the integrity of the data and the accuracy of the data analysis. CB affirms that the manuscript is an honest, accurate, and transparent account of the study being reported; that no important aspects of the study have been omitted and that any discrepancies from the study as planned have been explained.

Data sharing

To ensure patient privacy and confidentiality, data from the CPRD cannot be shared. Therefore, no additional data are available.

Copyright, open access and permission to reuse

The Corresponding Author has the right to grant on behalf of all authors and does grant on behalf of all authors, *a worldwide licence* to the Publishers and its licensees in perpetuity, in all forms, formats and media (whether known now or created in the future), to i) publish, reproduce, distribute, display and store the Contribution, ii) translate the Contribution into other languages, create adaptations, reprints, include within collections and create summaries, extracts and/or, abstracts of the Contribution, iii) create any other derivative work(s) based on the Contribution, iv) to exploit all subsidiary rights in the Contribution, v) the inclusion of electronic links from the Contribution to third party material where-ever it may be located; and, vi) licence any third party to do any or all of the above.

References

1 Alfonso C, Jann S, Massa R, et al. Diagnosis, treatment and follow-up of the carpal tunnel syndrome: A review. *Neurological Sciences* 2010;31:243-52.

2 Aroori S, Spence RA. Carpal tunnel syndrome. *Ulster Med J* 2008;77:6-17.

For peer review only - http://bmjopen.bmj.com/site/about/guidelines.xhtml

3 Padua L, Padua R, Lo Monaco M, et al. Multiperspective assessment of carpal tunnel syndrome: A multicenter study. *Neurology* 1999;53:1654-9.

4 Foley M, Silverstein B, Polissar N. The economic burden of carpal tunnel syndrome: long-term earnings of CTS claimants in Washington State. *Am J Ind Med* 2007;50:155-72.

5 Atroshi I, Gummesson C, Johnsson R, et al. Symptoms, disability, and quality of life in patients with carpal tunnel syndrome. *Journal of Hand Surgery - American Volume* 1999;24:398-404.

6 Padua L, Coraci D, Erra C, et al. Carpal tunnel syndrome: clinical features, diagnosis, and management. *Lancet Neurol* 2016;15:1273-84 doi:S1474-4422(16)30231-9 [pii].

7 American Academy of Orthopaedic Surgeons. Clinical practice guideline on the diagnosis of carpal tunnel syndrome 2007.

8 Carpal Tunnel Syndrome. Available at: http://cks.nice.org.uk/carpal-tunnel-syndrome#!scenariorecommendation:1. Accessed 05/14, 2013.

9 Carpal Tunnel Syndrome (CTS) - The Map of Medicine. Available at: http://app.mapofmedicine.com/mom/127/page.html?department-id=8&specialty-id=1037&pathway-id=3411&page-id=8741&history=clear. Accessed 25/10/2012, 2012.

10 Royal College of Surgeons. Commissioning Guide: Treatment of Carpal Tunnel Syndrome 2016.

11 Middleton SD, Anakwe RE. *BMJ* 2014;349 doi:10.1136/bmj.g6437.

12 Ferry S, Pritchard T, Keenan J, et al. Estimating the prevalence of delayed median nerve conduction in the general population. *Br J Rheumatol* 1998;37:630-5.

13 de Krom MC, Knipschild PG, Kester AD, et al. Carpal tunnel syndrome: prevalence in the general population. *J Clin Epidemiol* 1992;45:373-6.

14 Atroshi I. Carpal tunnel syndrome--prevalence, electrodiagnosis and outcome instruments. *Acta Orthop Scand* 1999;70:70-1.

15 Papanicolaou GD, McCabe SJ, Firrell J. The prevalence and characteristics of nerve compression symptoms in the general population. *Journal of Hand Surgery - American Volume* 2001;26:460-6.

16 Latinovic R, Gulliford MC, Hughes RA. Incidence of common compressive neuropathies in primary care. *J Neurol Neurosurg Psychiatry* 2006;77:263-5 doi:10.1136/jnnp.2005.066696.

17 Bland JDP, Rudolfer SM. Clinical surveillance of carpal tunnel syndrome in two areas of the United Kingdom, 1991–2001. *Journal of Neurology, Neurosurgery & Psychiatry* 2003;74:1674-9 doi:10.1136/jnnp.74.12.1674.

18 Gelfman R, Melton LJ,3rd, Yawn BP, et al. Long-term trends in carpal tunnel syndrome. *Neurology* 2009;72:33-41 doi:10.1212/01.wnl.0000338533.88960.b9; 10.1212/01.wnl.0000338533.88960.b9.

For peer review only

For peer review only - http://bmjopen.bmj.com/site/about/guidelines.xhtml

19 Bongers FJ, Schellevis FG, van den Bosch WJ, et al. Carpal tunnel syndrome in general practice (1987 and 2001): incidence and the role of occupational and non-occupational factors. *Br J Gen Pract* 2007;57:36-9.

20 Atroshi I, Englund M, Turkiewicz A, et al. Incidence of physician-diagnosed carpal tunnel syndrome in the general population. *Arch Intern Med* 2011;171:943-5.

21 Jenkins PJ, Watts AC, Duckworth AD, et al. Socioeconomic deprivation and the epidemiology of carpal tunnel syndrome. *Journal of Hand Surgery-European Volume* 2012;37E:123-9 doi:10.1177/1753193411419952.

22 Jenkins PJ, Srikantharajah D, Duckworth AD, et al. Carpal tunnel syndrome: the association with occupation at a population level. *Journal of Hand Surgery-European Volume* 2013;38E:67-72 doi:10.1177/1753193412455790.

23 Nordstrom DL, DeStefano F, Vierkant RA, et al. Incidence of diagnosed carpal tunnel syndrome in a general population. *Epidemiology* 1998;9:342-5.

24 Wildin C, Dias JJ, Heras-Palou C, et al. Trends in elective hand surgery referrals from primary care. *Ann R Coll Surg Engl* 2006;88:543-6 doi:10.1308/003588406X117070.

25 Bebbington E, Furniss D. Linear regression analysis of Hospital Episode Statistics predicts a large increase in demand for elective hand surgery in England. *J Plast Reconstr Aesthet Surg* 2015;68:243-51 doi:10.1016/j.bjps.2014.10.011 [doi].

26 Herrett E, Gallagher AM, Bhaskaran K, et al. Data Resource Profile: Clinical Practice Research Datalink (CPRD). *Int J Epidemiol* 2015;44:827-36 doi:10.1093/ije/dyv098 [doi].

27 Chisholm J. The Read clinical classification. *BMJ* 1990;300:1092.

28 Jordan K, Porcheret M, Croft P. Quality of morbidity coding in general practice computerized medical records: a systematic review. *Fam Pract* 2004;21:396-412 doi:10.1093/fampra/cmh409 [doi].

29 Population Estimates. Available at: https://www.ons.gov.uk/peoplepopulationandcommunity/populationandmigration/populationestimates. Accessed 02/16, 2017.

30 National Cancer Institute. Joinpoint Regression Program - Surveillance Research Program 2017.

31 Bedson J, Chen Y, Hayward RA, et al. Trends in long-term opioid prescribing in primary care patients with musculoskeletal conditions: an observational database study. *Pain* 2016;157:1525-31 doi:10.1097/j.pain.0000000000000557.

32 Descatha A, Dale AM, Franzblau A, et al. Comparison of research case definitions for carpal tunnel syndrome. *Scand J Work Environ Health* 2011;37:298-306 doi:10.5271/sjweh.3148.

33 NHS Executive. *Information for Health: An Information Strategy for the Modern NHS 1998–2005. A National Strategy for Local Implementation* 1998.

For peer review only - http://bmjopen.bmj.com/site/about/guidelines.xhtml

34 Porcheret M, Hughes R, Evans D, et al. Data quality of general practice electronic health records: the impact of a program of assessments, feedback, and training. *Journal of the American Medical Informatics Association* 2004;11:78-86 doi:http://dx.doi.org/10.1197/jamia.M1362.

35 Lewis R, Appleby J. Can the English NHS meet the 18-week waiting list target?. *J R Soc Med* 2006;99:10-3.

36 Pourmemari MH, Shiri R. Diabetes as a risk factor for carpal tunnel syndrome: a systematic review and meta-analysis. *Diabetic Med* 2016;33:10-6 doi:10.1111/dme.12855.

37 Shiri R, Pourmemari MH, Falah-Hassani K, et al. The effect of excess body mass on the risk of carpal tunnel syndrome: a meta-analysis of 58 studies. *Obesity Reviews* 2015;16:1094-104 doi:10.1111/obr.12324.

38 Herrett E, Thomas SL, Schoonen WM, et al. Validation and validity of diagnoses in the General Practice Research Database: a systematic review. *Br J Clin Pharmacol* 2009;69:4-14 doi:10.1111/j.1365-2125.2009.03537.x.

39 Jordan K, Clarke AM, Symmons DP, et al. Measuring disease prevalence: a comparison of musculoskeletal disease using four general practice consultation databases. *Br J Gen Pract* 2007;57:7-14.

40 Turner A, Kimble F, Gulyás K, et al. Can the outcome of open carpal tunnel release be predicted?: a review of the literature. *ANZ J Surg* 2010;80:50-4 doi:10.1111/j.1445-2197.2009.05175.x.

41 Louie DL, Earp BE, Collins JE, et al. *The Journal of Bone & Joint Surgery* 2013;95:1067-73 doi:10.2106/JBJS.L.00903.

42 Marshall S, Tardif G, Ashworth N. Local corticosteroid injection for carpal tunnel syndrome. *Cochrane database of systematic reviews (Online)* 2007:001554.

43 Page MJ, Massy-Westropp N, O'Connor D, et al. Splinting for carpal tunnel syndrome. *Cochrane Database Syst Rev* 2012;7:CD010003 doi:10.1002/14651858.CD010003.

44 Mondelli M, Giannini F, Giacchi M. Carpal tunnel syndrome incidence in a general population. *Neurology* 2002;58:289-94.

45 Dieleman JP, Kerklaan J, Huygen FJ, et al. Incidence rates and treatment of neuropathic pain conditions in the general population. *Pain* 2008;137:681-8 doi:10.1016/j.pain.2008.03.002 [doi].

46 Gelfman R, Melton LJ,3rd, Yawn BP, et al. Long-term trends in carpal tunnel syndrome. *Neurology* 2009;72:33-41 doi:10.1212/01.wnl.0000338533.88960.b9; 10.1212/01.wnl.0000338533.88960.b9.

47 Dale AM, Harris-Adamson C, Rempel D, et al. Prevalence and incidence of carpal tunnel syndrome in US working populations: Pooled analysis of six prospective studies. *Scandinavian Journal of Work, Environment and Health* 2013;39:495-505.

Supplementary tables

For peer review only - http://bmjopen.bmj.com/site/about/guidelines.xhtml

Suppl. Table 1. Demographics of the crude prevalent population presenting with CTS in each calendar year

Suppl. Table 2. Demographics of the crude incident population presenting with CTS in each calendar year

Suppl. Table 3 The age and sex standardised estimates of the annual prevalence and incidence of CTS

Suppl. Table 4 Comparison of population studies reporting the prevalence and / or incidence of carpal tunnel syndrome

Suppl. Table 5 summary of reported prevalence and incidence by gender

For peer review only

Table 1 Readcodes used to define a prevalent or incident episode of carpal tunnel syndrome

**Term**	**Read code**
Carpal tunnel syndrome	F340
Injection of carpal tunnel	85BE.00
Carpal tunnel release	70560
Endoscopic carpal tunnel release	7056011
Carpal tunnel decompression	70564

For peer review only

Table 2 Read codes used to define a surgical episode

Term	Read code
Carpal tunnel release	817
Re-release of carpal tunnel	16896
Endoscopic carpal tunnel release	39335
Revision of carpal tunnel release	97195
Carpal tunnel decompression	19249

For peer review only

Year	Number of person years	Prevalent individuals	Total crude prevalence per 10,000 person years, (95% confidence interval)	Female prevalence per 10,000 person years, (95% confidence interval)	Male prevalence per 10,000 person years, (95% confidence interval)	Female: male
1993	1117443	2909	26.03 (25.10 – 27.00)	37.52 (35.96 – 39.13)	13.69 (12.72 – 14.71)	2.74
1994	1198256	3188	26.61 (25.69 – 27.55)	37.23 (35.73 – 38.79)	15.21 (14.23 – 16.25)	2.45
1995	1286800	3343	25.98 (25.11 – 26.88)	36.64 (35.20 – 38.12)	14.58 (13.65 – 15.56)	2.51
1996	1437567	3706	25.78 (24.96 – 26.62)	36.75 (35.38 – 38.16)	14.09 (13.23 – 15.00)	2.61
1997	1681756	4190	24.91 (24.17 – 25.68)	34.87 (33.64 – 36.14)	14.34 (13.53 – 15.18)	2.43
1998	1899393	4884	25.71 (25.00 – 26.45)	36.57 (35.38 -37.79)	14.22 (13.46 – 15.01)	2.57
1999	2289158	5696	24.88 (24.24 – 25.54)	35.21 (34.14 – 36.30)	14.01 (13.32 – 14.72)	2.52
2000	2787457	6998	25.11 (24.52 – 25.70)	34.82 (33.86 – 35.81)	14.90 (14.26 – 15.57)	2.34
2001	3057458	8137	26.61 (26.04 – 27.20)	36.46 (35.52 – 37.42)	16.31 (15.67 – 16.98)	2.23
2002	3385511	9722	28.72 (28.15 – 29.29)	39.33 (38.40 – 40.28)	17.64 (17.00 – 18.29)	2.23
2003	3552908	11124	31.31 (30.73 – 31.90)	43.61 (42.66 – 44.59)	18.53 (17.90 – 19.18)	2.35
2004	3712172	12622	34.00 (33.41 – 34.60)	47.20 (46.23 – 48.19)	20.33 (19.68 – 20.99)	2.32
2005	3808183	12741	33.46 (32.88 – 34.04)	46.37 (45.42 – 47.34)	20.09 (19.45 – 20.74)	2.31
2006	3857487	12718	32.97 (32.40 – 33.55)	45.82 (44.88 – 46.78)	19.69 (19.07 – 20.33)	2.33
2007	3904068	13222	33.87 (33.29 – 34.45)	46.35 (45.41 – 47.31)	20.99 (20.35 – 21.65)	2.21
2008	3897624	14030	36.00 (35.40 – 36.60)	49.12 (48.15 – 50.11)	22.46 (21.79 – 23.14)	2.19
2009	3894989	14500	37.23 (36.60 – 37.81)	50.68 (49.69 – 51.68)	23.35 (22.68 – 24.05)	2.17
2010	3842773	14166	36.86 (36.26 – 37.48)	49.75 (48.76 – 50.75)	23.57 (22.88 – 24.27)	2.11
2011	3769676	13529	35.89 (35.29 – 36.50)	47.98 (47.00 – 48.97)	23.36 (22.67 – 24.07)	2.05
2012	3714877	13388	36.04 (35.43 – 36.66)	47.57 (46.59 – 48.56)	24.05 (23.35 – 24.78)	1.98
2013	3473094	12532	36.08 (35.45 – 36.72)	47.19 (46.18 – 48.21)	24.49 (23.75 – 25.25)	1.93

Table 3 Crude prevalence of carpal tunnel syndrome (n/10,000 person years) per calendar year, as presented in UK primary care (CPRD)

For peer review only

For peer review only

Table 4 Crude incidence of carpal tunnel syndrome (n/10,000 person years) per calendar year, as presented in UK primary care (CPRD)

Year	Number of person years	Incident individuals	Total crude incidence per 10,000 person years, (95% confidence interval)	Female incidence per 10,000 person years, (95% confidence interval)	Male incidence per 10,000 person years, (95% confidence interval)	Female: male
1993	783330	1584	20.22 (19.24 – 21.24))	28.72 (27.09 – 30.42)	11.17 (10.14 – 12.29)	2.57
1994	868616	1797	20.69 (19.74 – 21.67)	28.52 (26.97 – 30.13)	12.38 (11.34 – 13.69)	2.30
1995	1003593	1963	19.56 (18.70 – 20.45)	27.53 (26.12 – 29.00)	11.12 (10.20 – 12.10)	2.48
1996	1065068	2142	20.11 (19.27 – 20.98)	28.39 (27.00 – 29.84)	11.37 (10.47 – 12.33)	2.50
1997	1150299	2306	20.05 (19.24 – 20.88)	28.39 (27.05 – 29.79)	11.25 (10.39 – 12.16)	2.52
1998	1300074	2696	20.74 (19.95 – 21.52)	29.65 (28.57 – 31.22)	11.37 (10.56 – 12.23)	2.61
1999	1497673	3030	20.23 (19.52 – 20.10)	28.53 (27.35 – 29.75)	11.54 (10.77 – 12.34)	2.47
2000	1682027	3462	20.58 (19.90 – 21.28)	28.66 (27.54 – 29.81)	12.15 (11.41 – 12.93)	2.36
2001	2019596	4391	21.74 (21.10 – 22.40)	29.72 (28.68 – 30.79)	13.46 (12.74 – 14.20)	2.21
2002	2456761	5718	23.27 (22.68 – 31.78)	31.78 (30.78 – 32.79)	14.47 (13.80 – 15.17)	2.20
2003	2669111	6772	25.37 (24.77 – 25.98)	35.13 (34.14 – 36.14)	15.33 (14.67 – 16.02)	2.29
2004	2779821	7868	28.30 (27.68 – 28.94)	39.22 (38.19 – 40.27)	17.10 (16.42 – 17.81)	2.29
2005	3164506	8113	25.64 (25.08 – 26.20)	35.55 (34.63 – 36.48)	15.49 (14.88 – 16.12)	2.30
2006	3307051	8337	25.21 (24.67 – 25.76)	34.91 (34.02 – 35.82)	15.27 (14.68 – 15.89)	2.29
2007	3343009	8865	26.52 (25.97 – 27.08)	35.76 (34.86 – 36.67)	17.07 (16.45 – 17.71)	2.09
2008	3341299	9437	28.24 (27.68 – 28.82)	38.23 (37.30 – 39.17)	18.06 (17.42 – 18.72)	2.12
2009	3383196	9918	29.32 (28.74 – 29.90)	39.73 (38.79 – 50.68)	18.69 (18.04 – 19.36)	2.13
2010	3357338	9634	28.70 (28.13 – 29.27)	38.70 (37.77 – 39.64)	18.46 (17.82 – 19.13)	2.10
2011	3269296	9083	27.78 (27.21 – 28.36)	37.11 (36.19 – 38.05)	18.20 (17.54 – 18.87)	2.04
2012	3222880	9011	27.96 (27.39 – 28.54)	36.44 (35.52 – 37.88)	19.23 (18.56 – 19.93)	1.89
2013	3015670	8346	27.68 (27.09 – 28.28)	35.95 (35.01 – 36.92)	19.12 (18.43 – 19.84)	1.88

For peer review only

Table 5 Percentage of patients with carpal tunnel syndrome with a recorded episode of carpal tunnel release surgery per calendar year, as presented in UK primary care (CPRD)

Year	Episodes per 10,000 person years	% prevalent individuals having surgery	% prevalent females having surgery	% prevalent males having surgery	Female median age (25% - 75% Interquartile range)	Male median age (25% - 75% Interquartile range)
1993	5.04	19.35	18.78	21.03	53 (43 – 64)	55 (44 – 69)
1994	5.70	21.42	20.62	23.52	53 (43 – 68)	58 (45 – 70)
1995	6.19	23.81	23.40	24.92	53 (42 – 67)	55 (44 – 70)
1996	5.41	20.99	20.48	22.43	53 (44 – 65)	52 (40 – 65)
1997	5.70	22.89	22.14	24.81	53 (45 – 67)	56 (42 – 69)
1998	5.73	22.28	21.28	25.00	53 (44 – 65)	53 (44 – 65)
1999	6.24	25.09	24.60	26.38	54 (44 – 67)	56 (46 – 70)
2000	6.41	25.54	24.84	27.23	54 (44 – 68)	56 (45 – 69)
2001	6.88	25.87	25.95	25.68	55 (45 – 68)	58 (46 – 71)
2002	7.02	24.46	24.19	25.09	57 (46 – 71)	55 (45 – 68)
2003	8.26	26.39	25.88	27.66	56 (45 – 67)	57 (46 – 71)
2004	9.34	27.48	27.38	27.74	56 (46 – 67)	57 (47 – 68)
2005	9.70	29.00	28.31	30.65	57 (47 – 68)	58 (46 – 71)
2006	9.36	28.40	28.31	28.61	57 (47 – 68)	60 (48 – 72)
2007	9.71	28.66	28.26	29.59	56 (46 – 69)	59 (48 – 71)
2008	10.53	29.25	29.00	29.82	56 (46 – 68)	60 (49 – 72)
2009	10.92	29.32	28.73	30.66	56 (46 – 70)	61 (49 – 72)
2010	10.40	28.22	27.57	29.62	57 (47 – 71)	61 (48 – 73)
2011	9.47	26.37	26.11	26.93	57 (47 – 70)	61 (49 – 73)
2012	9.48	26.31	25.89	27.19	57 (47 – 71)	60 (49 – 73)
2013	9.89	27.41	26.47	29.30	57 (48 – 70)	62 (51 – 74)

For peer review only

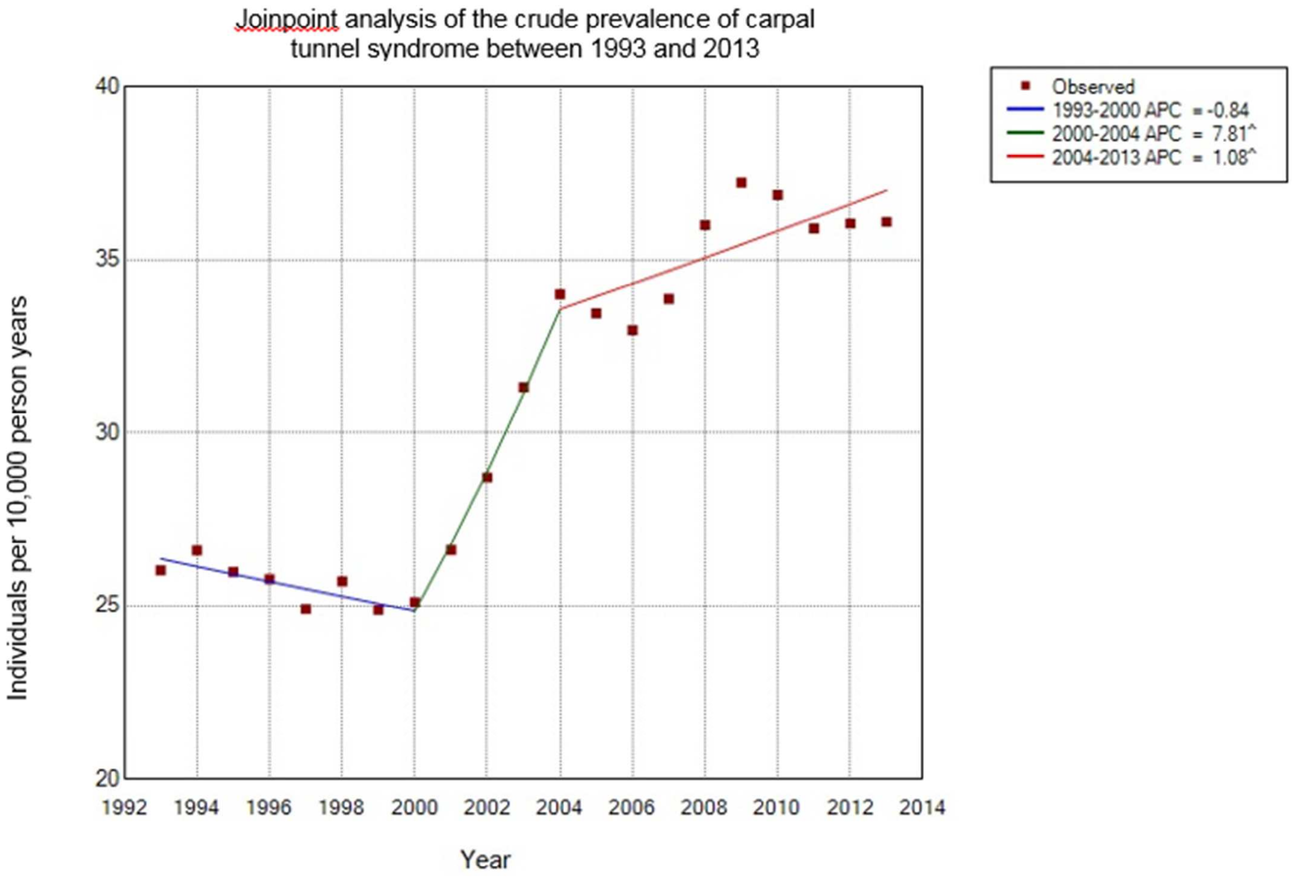

Figure 1 Joinpoint analysis of the crude prevalence of carpal tunnel syndrome between 1993 and 2013

APC = annual percentage change, which is significantly different from zero, significance level $p < 0.05$

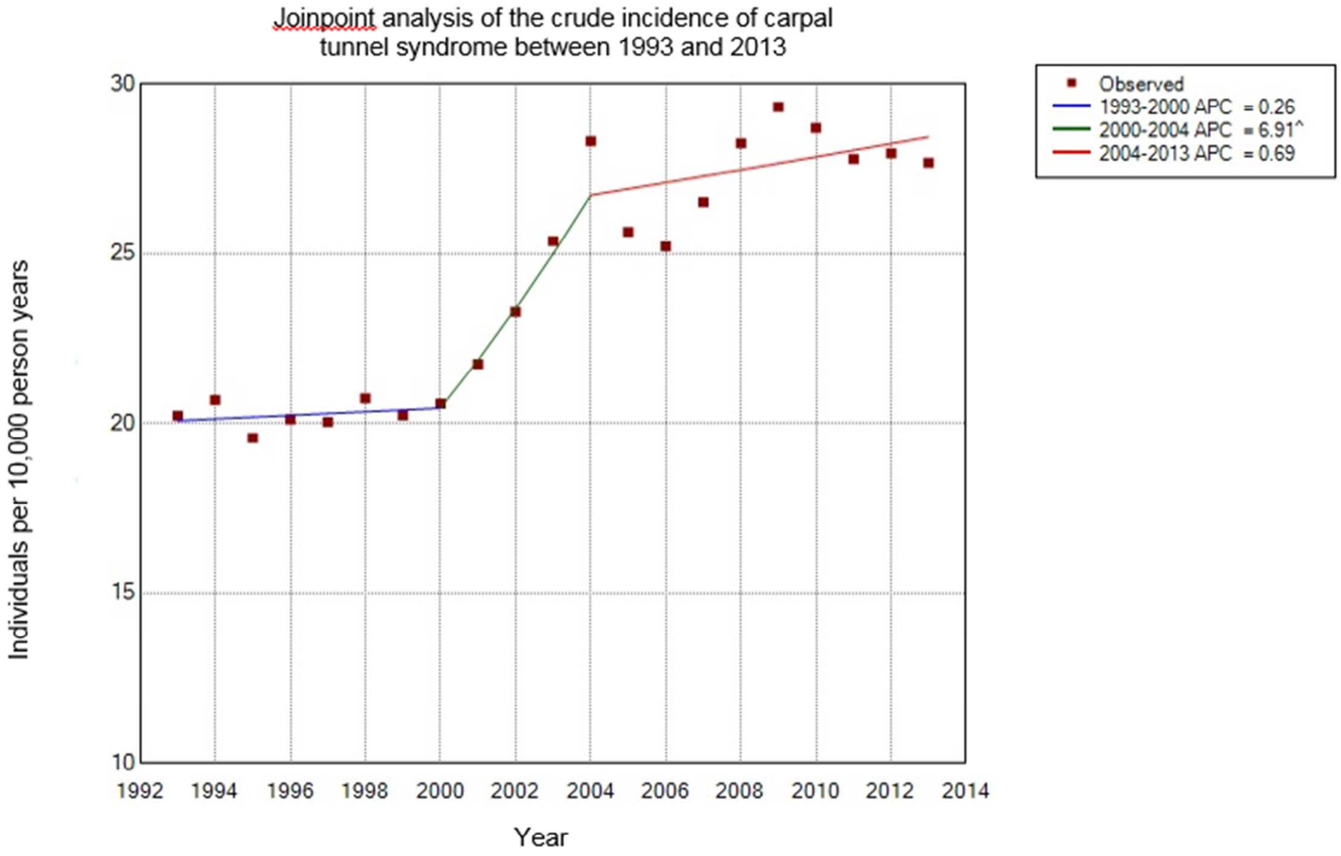

Figure 2 Joinpoint analysis of crude incidence

APC = annual percentage change, which is significantly different from zero, significance level $p < 0.05$

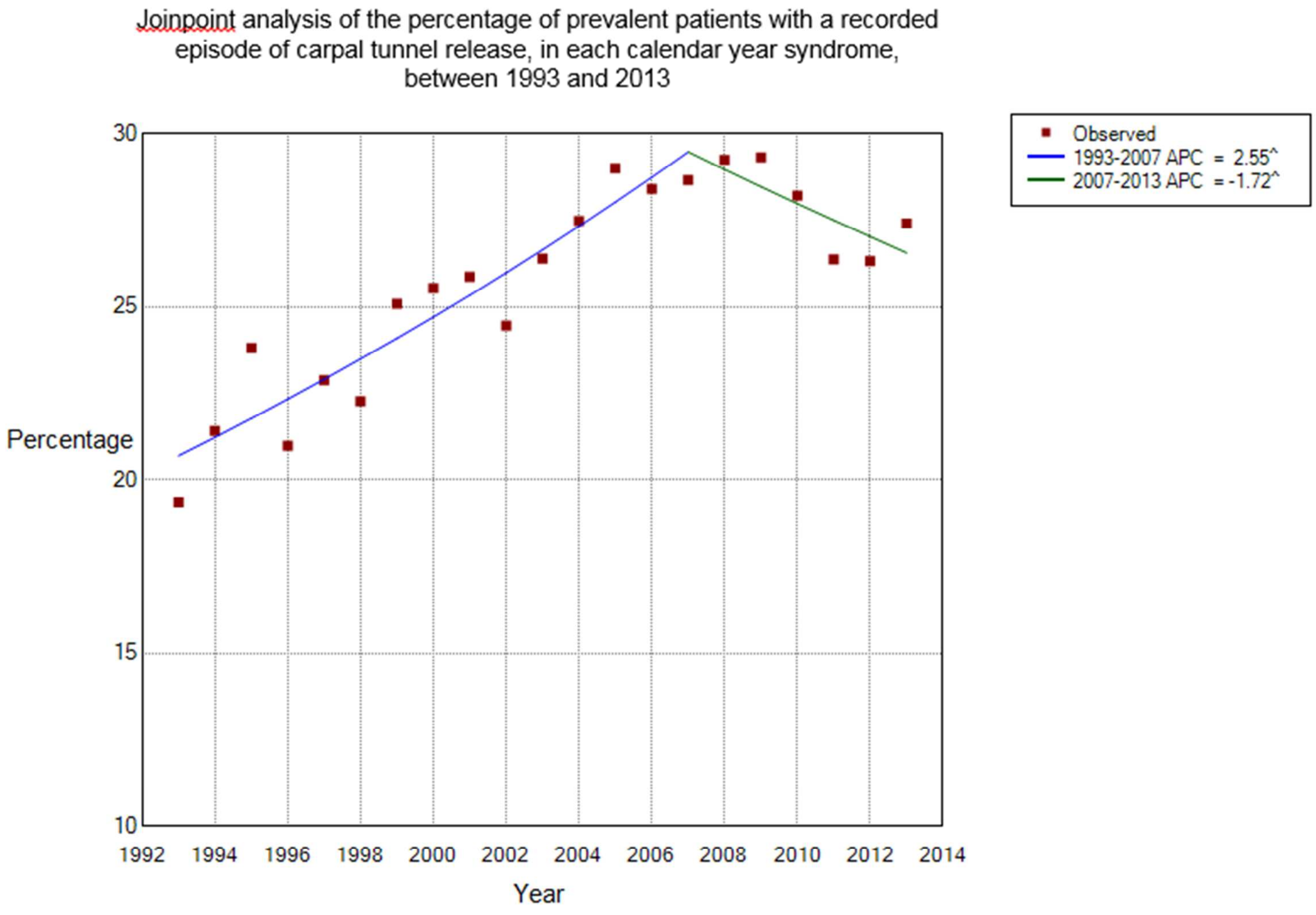

Figure 3 Joinpoint analysis of percentage of prevalent patients having carpal tunnel surgery

APC = annual percentage change, which is significantly different from zero, significance level $p < 0.05$

Suppl. Table 1. Demographics of the crude prevalent population presenting with CTS in each calendar year

Year	Female median age (25% - 75% Interquartile range)	Male median age (25% - 75% Interquartile range)
1993	49 (38 – 62)	53 (42 – 66)
1994	49 (39 – 62)	53 (42 – 66)
1995	50 (39 – 62)	52 (41 – 64)
1996	50 (40 – 62)	53 (41 – 66)
1997	51 (40 – 62)	53 (42 – 67)
1998	51 (40 – 62)	54 (43 – 67)
1999	51 (40 – 62)	54 (44 – 66)
2000	52 (41 – 64)	55 (44 – 67)
2001	53 (42 – 65)	55 (44 – 68)
2002	53 (41 – 64)	55 (44 – 67)
2003	54 (42 – 65)	55 (44 – 68)
2004	55 (43 – 65)	56 (45 – 68)
2005	54 (43 – 65)	58 (45 – 70)
2006	54 (43 – 66)	58 (45 – 70)
2007	54 (42 – 66)	54 (42 – 66)
2008	54 (43 – 66)	58 (46 – 70)
2009	54 (43 – 67)	58 (47 – 70)
2010	54 (43 – 67)	57 (46 – 71)
2011	54 (43 – 67)	58 (47 – 71)
2012	54 (43 – 67)	59 (48 – 71)
2013	54 (44 – 67)	59 (48 – 72)

For peer review only

Suppl. Table 2. Demographics of the crude incident population presenting with CTS in each calendar year

Year	Female median age (25% - 75% Interquartile range)	Male median age (25% - 75% Interquartile range)
1993	50 (39 – 63)	51 (42 – 65)
1994	50 (40 – 63)	53 (43 – 66)
1995	51 (40 – 63)	53 (42 – 64)
1996	51 (40 – 64)	52 (41 – 65)
1997	51 (40 – 64)	55 (45 – 67)
1998	51 (40 – 63)	54 (44 – 68)
1999	52 (41 – 64)	55 (45 – 67)
2000	53 (42 – 65)	55 (44 – 68)
2001	53 (42 – 66)	55 (45 – 68)
2002	54 (42 – 66)	55 (44 – 67)
2003	55 (43 – 66)	56 (45 – 68)
2004	55 (44 – 66)	57 (45 – 68)
2005	55 (43 – 66)	58 (46 – 70)
2006	55 (44 – 67)	58 (46 – 70)
2007	54 (43 – 66)	58 (47 – 70)
2008	55 (44 – 67)	58 (47 – 70)
2009	55 (44 – 67)	59 (47 – 71)
2010	55 (44 – 68)	57 (47 – 70)
2011	55 (44 – 68)	59 (48 – 71)
2012	54 (44 – 67)	59 (48 – 71)
2013	55 (45 – 69)	59 (48 – 71)

For peer review only

Suppl. Table 3 The age and sex standardised estimates of the annual prevalence and incidence of CTS

Year	Age sex standardised prevalence (per 10,000 person years, 95% CI)	Age sex standardised incidence (per 10,000 person years, 95% CI)
1993	26.27 (26.13 – 26.42)	19.95 (19.83 – 20.07)
1994	26.83 (26.69 – 26.98)	20.46 (20.34 – 20.59)
1995	25.90 (25.77 – 26.05)	19.20 (19.08 – 19.33)
1996	25.64 (25.50 – 25.78)	19.61 (19.49 – 19.74)
1997	24.64 (24.20 – 25.07)	19.42 (19.30 – 19.55)
1998	25.42 (25.88 – 25.56)	20.05 (19.93 – 20.18)
1999	24.57 (24.44 – 24.71)	19.51 (19.39 – 19.64)
2000	24.77 (24.63 – 24.91)	19.73 (19.61 – 19.86)
2001	26.22 (26.08 – 26.36)	20.75 (20.63 – 20.88)
2002	28.22 (28.07 – 28.37)	22.22 (22.10 – 22.36)
2003	30.81 (30.65 – 30.96)	24.28 (24.15 – 24.42)
2004	33.51 (33.35 – 33.67)	27.00 (26.86 – 27.14)
2005	32.98 (32.82 – 33.14)	24.56 (24.42 – 24.70)
2006	32.55 (32.39 – 32.70)	24.14 (24.00 – 24.27)
2007	33.48 (33.32 – 33.64)	25.52 (25.38 – 25.66)
2008	35.59 (35.43 – 25.76)	27.07 (26.92 – 27.21)
2009	36.81 (36.64 – 36.98)	28.19 (28.05 – 28.34)
2010	36.40 (36.24 – 36.66)	27.53 (27.39 – 27.68)
2011	35.28 (35.12 – 35.44)	26.59 (26.45 – 26.74)
2012	35.50 (35.34 – 35.67)	26.75 (26.61 – 26.89)
2013	35.45 (35.29 – 35.61)	26.34 (26.01 – 26.49)

For peer review only

Suppl. Table 4 Comparison of population studies reporting the prevalence and / or incidence of carpal tunnel syndrome

**Study Identifier**	**Study method**	**Definition of CTS**	**Comments**
De Krom et al. 1992	Survey of a random age sex stratified sample of the general population taken from the population register of Maastricht between 1983 and 1985	Questionnaire based on symptoms and signs	[13]
Ferry et al. 1998	i) Cross sectional survey to estimate the point prevalence of hand symptoms (from a random sample of 1000 individuals from the UK general population, aged 18 to 75 years) and ii) nerve conduction testing of a weighted sample<br>- Circa. 1998 (not stated)<br>- point prevalence determined	Based on nerve conduction studies using defined cut offs	Subjects over 54yrs had a higher prevalence than younger participants. No difference between genders was noted.[12]
Nordstrom et al. 1998	Prospective study conducted in the general population of the Marshfield Epidemiologic Study Area, Wisconsin, between 1991 and 1993	1. any diagnosis of possible, probable or definite CTS;<br>2. any diagnosis of probable or definite CTS; and<br>3. any diagnosis of possible , probable or definite CTS plus at least one of six clinical signs	A 3.5 fold increase in CTS incidence was noted compared with data from 20 years previously in the same study population[23]
Atroshi et al. 2000	Survey of a random sample of the age sex stratified general population of Southern Sweden, in 1997	Diagnosis based on clinical examination and positive electrophysiological findings	The population prevalence of symptoms was 14.4%; the prevalence of clinically and electrophysiologically confirmed CTS was 2.7% [14]
Papanicolaou, McCable & Firrell 2001	Cross-sectional study to evaluate prevalence of carpal tunnel syndrome in the General population of the United States	Katz hand diagram	After correcting for nonresponders the lowest possible estimate of CTS was 3.72% [15]

For peer review only

Mondelli, Giannini & Giacchi 2002	Prospective study of patients referred to four electrodiagnostic laboratories in the Siena area, Italy. Mean annual incidence calculated from time period 1991 to 1998	Diagnosis based on clinical history and electrodiagnostic evidence of a reduced distal conduction velocity of the median nerve (American Academy of Neurology standards)	Of the patients presenting 79.7% were women. The mean age at diagnosis was 55.0 +/- 14.4 years (range 16 to 97) [44]
Bland, Rudolfer 2003	Prospective collection of neurophysiological and clinical data of patients referred to two electromyography clinics in the UK between 1991 to 1993 and 1992 to 2001	Based on nerve conduction studies using defined cut offs	An increase in diagnosed cases was observed between the two data collection periods; attributed to referral of milder cases. Median nerve impairment was more severe in the elderly and men at all ages. [17]
Latinovic, Gulliford & Hughes 2006	Population study based in a general practice database of consulting primary care patients from 253 practices between January 1992 and 31 December 2000.	Read and Oxmis codes for carpal tunnel syndrome	Most frequent in women aged 45-54. In 2000 operative treatment was undertaken for 31% of incident CTS presentations [16]
Bonger et al. 2007	Analysis of the first and second Dutch National Survey of General Practice, conducted in 1987 and 2001	(International Classification of Primary Care) ICPC coded diagnosis	A crude increase in incidence over time was not statistically significant after subdividing by age and sex. Incidence rates were related to the job level in women, but not men [19]
Dieleman et al. 2008	Population study based in a general practice database (Integrated Primary Care Information (IPCI) database): data of consulting primary care patients in the Netherlands between 1996 and 2003	ICPC coded diagnosis	Neuropathic pain was noted to affect almost 1% of the population. Mononeuropathies and carpal tunnel syndrome were the most common causes [45]
Gelfman et al. 2009	Analysis of medical records linkage system 1981-1985 to 2000-2005 of residents of Olmsted County, Minnesota (Rochester Epidemiology Project)	Clinical coding with a sample verified by full record review	An increase in incidence was observed over the study period. An increase in young individuals seeking care for less severe CTS in the mid-1980's was followed in the 1990's by an increasing incidence in older people [46]
Atroshi et al. 2011	Analysis of the Skane Health Care	Physician diagnosed	[20]

For peer review only

	Register (SCHR) (inhabitants presenting to public health providers), incident cases identified between 2006 - 2008		
Jenkins et al. 2012b	Prospective audit of patients referred to a regional hand service based in secondary care in Scotland between November 2004 and May 2010	Symptoms of pain or paraesthesia in the median nerve distribution and one or more of: nerve conduction deficit, thenar muscle wasting or positive Tinel or Phalen signs	Mean age of presentation 55.1years (range 22 to 96, SD 13.5 years).<br>Mean body mass index at presentation 29.5 kg/m2<br>CTS more common in: females (OR 1.9, 95% CI 1.5 to 2.5)<br>Incidence varied significantly between deprivation groups: most deprived 81/100,000 and least deprived 62/100,000 (OR 1.3, 95% CI 1.1 to 1.6) [21]
Jenkins et al. 2013	Prospective audit of patients referred to a regional hand service based in secondary care in Scotland between November 2004 and May 2010, who were employed	Clinical diagnosis based on history and examination, in most cases substantiated by nerve conduction studies	The greatest incidence as in caring and leisure occupations (197 per 100,000) and the lowest incidence was in the associate professional group (37 per 100,000) [22]
Dale 2013	Pooled analysis of six prospective studies collecting data from >50 workplaces, over variable time frames	A pooled case definition was derived to include clinical and elctrodiagnostic criteria	7.8% of 4321 subjects studied had prevalent CTS, with an additional 204 subjects meeting the CTS criteria, leading to an incidence of 2.3 cases per 100 person years [47]

For peer review only

Suppl. Table 5 summary of reported prevalence and incidence by gender

Study Identifier	Country of Origin Data collection (Prevalence or Incidence)	Prevalence or Incidence per 100,000, per annum			Female / male ratio
		All	Female	Male	
De Krom et al. 1992	The Netherlands 1983 - July 1985 (Prevalence)	5700	5800	600	9.66
Atroshi et al. 2000	Sweden 1997 (Prevalence)	3800	4600	2800	1.64
Papanicolaou, McCable & Firrell 2001	United States 2001 (Prevalence)	3720			4.8
Ferry et al. 1998	United Kingdom Not stated (Incidence)	8200	6400	8200	0.78
Nordstrom et al. 1998	United States 1991 - 1993 (Incidence)	346	373	318	1.17
Mondelli, Giannini & Giacchi 2002	Italy 1991 – 1998 (mean) (Incidence)	276	506	139	3.64
Bland, Rudolfer 2003	Kent, UK 1991 - 2001 (Incidence)	105	120.5	60	2
	Huddersfield, UK		61.5	30	2

For peer review only

Latinovic, Gulliford & Hughes 2006	United Kingdom (Incidence)		192.8	87.8	2.23
Bongers et al. 2007	The Netherlands (Incidence) 1987	130	190	60	3.17
	2001	180	280	90	3.11
Dieleman et al. 2008	The Netherlands 1996 - 2003 (Incidence)	233.1			
Gelfman et al. 2009	United States (Incidence) 1981-1985	258	337	177	1.90
	2001-2005	424	542	303	1.79
Atroshi et al. 2011	Sweden 2006 - 2008 (Incidence)		428	182	2.35
Jenkins et al. 2012b	Scotland 2004 - 2010 (Incidence)	72	98	43	2.28
Jenkins et al. 2013	Scotland 2004 - 2010 (Incidence)	103			
Dale 2013	United States (Incidence)	2300			

For peer review only

STROBE Statement—checklist of items that should be included in reports of observational studies

	Item No	Recommendation	Page
**Title and abstract**	1	(*a*) Indicate the study's design with a commonly used term in the title or the abstract	1-2
		(*b*) Provide in the abstract an informative and balanced summary of what was done and what was found	1-2
**Introduction**			
Background/rationale	2	Explain the scientific background and rationale for the investigation being reported	4
Objectives	3	State specific objectives, including any prespecified hypotheses	4-5
**Methods**			
Study design	4	Present key elements of study design early in the paper	5
Setting	5	Describe the setting, locations, and relevant dates, including periods of recruitment, exposure, follow-up, and data collection	5-6
Participants	6	(*a*) *Cohort study*—Give the eligibility criteria, and the sources and methods of selection of participants. Describe methods of follow-up<br>*Case-control study*—Give the eligibility criteria, and the sources and methods of case ascertainment and control selection. Give the rationale for the choice of cases and controls<br>*Cross-sectional study*—Give the eligibility criteria, and the sources and methods of selection of participants	5-6
		(*b*) *Cohort study*—For matched studies, give matching criteria and number of exposed and unexposed<br>*Case-control study*—For matched studies, give matching criteria and the number of controls per case	
Variables	7	Clearly define all outcomes, exposures, predictors, potential confounders, and effect modifiers. Give diagnostic criteria, if applicable	5-6
Data sources/ measurement	8*	For each variable of interest, give sources of data and details of methods of assessment (measurement). Describe comparability of assessment methods if there is more than one group	na
Bias	9	Describe any efforts to address potential sources of bias	na
Study size	10	Explain how the study size was arrived at	na
Quantitative variables	11	Explain how quantitative variables were handled in the analyses. If applicable, describe which groupings were chosen and why	6
Statistical methods	12	(*a*) Describe all statistical methods, including those used to control for confounding	6
		(*b*) Describe any methods used to examine subgroups and interactions	na
		(*c*) Explain how missing data were addressed	na
		(*d*) *Cohort study*—If applicable, explain how loss to follow-up was addressed<br>*Case-control study*—If applicable, explain how matching of cases and controls was addressed<br>*Cross-sectional study*—If applicable, describe analytical methods taking account of sampling strategy	na
		(*e*) Describe any sensitivity analyses	

For peer review only

Continued on next page.

**Results**			
Participants	13*	(a) Report numbers of individuals at each stage of study—eg numbers potentially eligible, examined for eligibility, confirmed eligible, included in the study, completing follow-up, and analysed	Na
		(b) Give reasons for non-participation at each stage	Na
		(c) Consider use of a flow diagram	Na
-Descriptive data	14*	(a) Give characteristics of study participants (eg demographic, clinical, social) and information on exposures and potential confounders	6-7
		(b) Indicate number of participants with missing data for each variable of interest	na
		(c) *Cohort study*—Summarise follow-up time (eg, average and total amount)	na
Outcome data	15*	*Cohort study*—Report numbers of outcome events or summary measures over time	
		Case-control study—Report numbers in each exposure category, or summary measures of exposure	
		Cross-sectional study—Report numbers of outcome events or summary measures	6-7
Main results	16	(*a*) Give unadjusted estimates and, if applicable, confounder-adjusted estimates and their precision (eg, 95% confidence interval). Make clear which confounders were adjusted for and why they were included	6-7 + tables
		(*b*) Report category boundaries when continuous variables were categorized	na
		(*c*) If relevant, consider translating estimates of relative risk into absolute risk for a meaningful time period	na
Other analyses	17	Report other analyses done—eg analyses of subgroups and interactions, and sensitivity analyses	na
**Discussion**			
Key results	18	Summarise key results with reference to study objectives	8
Limitations	19	Discuss limitations of the study, taking into account sources of potential bias or imprecision. Discuss both direction and magnitude of any potential bias	10
Interpretation	20	Give a cautious overall interpretation of results considering objectives, limitations, multiplicity of analyses, results from similar studies, and other relevant evidence	8-11
Generalisability	21	Discuss the generalisability (external validity) of the study results	10
**Other information**			
Funding	22	Give the source of funding and the role of the funders for the present study and, if applicable, for the original study on which the present article is based	11

*Give information separately for cases and controls in case-control studies and, if applicable, for exposed and unexposed groups in cohort and cross-sectional studies.

Note: An Explanation and Elaboration article discusses each checklist item and gives methodological background and published examples of transparent reporting. The STROBE checklist is best used in conjunction with this article (freely available on the Web sites of PLoS Medicine at http://www.plosmedicine.org/, Annals of Internal Medicine at http://www.annals.org/, and Epidemiology at http://www.epidem.com/). Information on the STROBE Initiative is available at www.strobe-statement.org.

For peer review only - http://bmjopen.bmj.com/site/about/guidelines.xhtml

BMJ Open

Trends in the prevalence, incidence and surgical management of carpal tunnel syndrome between 1993 and 2013: an observational analysis of UK primary care records

Journal:	*BMJ Open*
Manuscript ID	bmjopen-2017-020166.R1
Article Type:	Research
Date Submitted by the Author:	02-Feb-2018
Complete List of Authors:	Burton, Claire; Keele University, Arthritis Research UK Primary Care Centre, Research Institute for Primary Care & Health Sciences<br>Chen, Ying; Keele University, Arthritis Research UK Primary Care Centre, Research Institute for Primary Care & Health Sciences<br>Chesterton, Linda; Keele University, Arthritis Research UK Primary Care Centre, Research Institute for Primary Care & Health Sciences<br>van der Windt, Danielle; Keele University, Arthritis Research UK Primary Care Centre, Research Institute for Primary Care & Health Sciences
**Primary Subject Heading**:	Epidemiology
Secondary Subject Heading:	Rheumatology, General practice / Family practice
Keywords:	PRIMARY CARE, SURGERY, Musculoskeletal disorders < ORTHOPAEDIC & TRAUMA SURGERY, Hand & wrist < ORTHOPAEDIC & TRAUMA SURGERY

SCHOLARONE™
Manuscripts

For peer review only

Trends in the prevalence, incidence and surgical management of carpal tunnel syndrome between 1993 and 2013: an observational analysis of UK primary care records

Authors

Claire L. Burton[a] c.burton@keele.ac.uk GP Research Fellow

Ying Chen[a] Research[a] Research Associate in Biostatistics

Linda S. Chesterton[a] Senior Lecturer in Physiotherapy

Danielle A. van der Windt[a] Professor of Primary Care Epidemiology

[a] Arthritis Research UK Primary Care Centre, Research Institute for Primary Care & Health Sciences, Keele University, Staffordshire, England. ST5 5BG

Word count (excluding title page, abstract, references, figures and tables) 4266

For peer review only - http://bmjopen.bmj.com/site/about/guidelines.xhtml

Abstract

Objectives

To describe the prevalence, incidence and surgical management of carpal tunnel syndrome (CTS), between 1993 and 2013, as recorded in the Clinical Practice Research Datalink (CPRD)

Design

We completed a series of cross-sectional epidemiological analyses to observe trends over time.

Setting

Primary care data collected between 1993 and 2013, stored in the Clinical Practice Research Datalink

Population

Individuals ≥ 18 years were selected. Prevalent and incident episodes of Carpal Tunnel syndrome (CTS) and episodes surgical intervention were identified using a list of pre-identified Read codes.

Analysis

We defined incident episodes as those with no preceding diagnostic code for CTS in the past 2 years of data. Episodes of surgery were expressed as a percentage of the prevalent population during the same calendar year. Joinpoint regression was used to determine significant changes in the underlying trend.

Results

The prevalence of CTS increased over the study period, with a particular incline between 2000 and 2004 (annual percentage change 7.81). The female to male prevalence ratio reduced over time from 2.74 in 1993 to 1.93 in 2013. The median age of females and males with CTS were noted to increase from 49 and 53 years respectively in 1993 to 54 and 59 years respectively in 2013. Incidence was also noted to increase over time. After an initial increase between 1993 and 2007, the percentage of prevalent patients with a coded surgical episode began to decrease after 2007 to 27.41% in 2013 (annual percentage change -1.7)

Conclusion

This study has demonstrated that the prevalence and incidence of carpal tunnel syndrome increased over the study period between 1993 and 2013. Rates of surgery for CTS also increased over the study period, however after 2007, the percent of patients receiving surgery showed a statistically significant decline back to the rate seen in 2004.

Key words

Carpal tunnel syndrome; primary care; epidemiology; incidence; prevalence; surgery

For peer review only - http://bmjopen.bmj.com/site/about/guidelines.xhtml

Strengths and Limitations

- Provides updated epidemiological data about a common and bothersome condition
- Set in primary care, where most cases of carpal tunnel syndrome present
- Utilises a large primary care database, generalizable to the UK population
- Relies on the correct coding and capture of episodes of carpal tunnel syndrome and carpal tunnel release surgery

For peer review only

For peer review only - http://bmjopen.bmj.com/site/about/guidelines.xhtml

INTRODUCTION

Carpal tunnel syndrome (CTS) is a chronic focal compressive neuropathy caused by the entrapment of the median nerve at the level of the carpal tunnel in the wrist.[1] CTS is the most common presentation of the entrapment neuropathies [2] and is characterised by symptoms including paraesthesia, dysesthesia, sensory loss and eventually weakness and atrophy of the thenar muscle. Symptoms are usually localised to the hand but can spread proximally to the forearm, upper arm and even shoulder.[3] Despite causing relatively localised symptoms, CTS can have substantial physical, psychological and economic consequences.[4, 5] In some cases, there may be associations with certain occupations (such as the care and leisure industry)[6] which involve the overuse of the hand and wrist as well as other physical comorbidities including: pregnancy; diabetes; hypothyroidism and obesity.[7]

The diagnosis of CTS is generally accepted to be a clinical one (based on history and examination findings) [8], although electrodiagnostic tests are commonly requested to confirm the diagnosis or differentiate among diagnoses, especially in the presence of thenar atrophy and / or persistent numbness or if surgical management is being considered.[9] The treatment of CTS is usually defined as either surgical or conservative (non-surgical). Local steroid injections and night splinting form the mainstay of primary care interventions in carpal tunnel syndrome, as indicated by national care pathways.[10, 11] Patients with moderate, severe or deteriorating symptoms following conservative treatment or sudden and severe symptoms are recommended to be referred for consideration of surgery.[12] Carpal tunnel release surgery (CTR) is routinely carried out under local anaesthetic as day surgery. Open and endoscopic approaches are used to release the flexor retinaculum.[13] Previous studies have sought to estimate the prevalence and / or incidence of CTS. Such epidemiological studies have been diverse in their approach to the populations studied and case definitions applied.[14] The reported estimates for annual prevalence range from 3720-5700 per 100,000 per year [15-17] and the reported incidence from 72 – 8200 per 100,000 per year.[6, 14, 18-23] CTS is generally accepted to be more common in women; the female to male ratio ranges between 0.78 and 9.66 [14, 15] A number of previous studies have observed the trends of prevalence or incidence over time and identified an increase [19, 20, 24], with 2005 being the latest data collection point. The most recent primary care based study in the UK utilised data from between 1992 and 2000.[18]

Episodes of CTR have also been shown to have increased, with audit data from one major tertiary UK Hand Centre suggesting that referral for CTR increased over a 10 year period from 59.7 to 112 per 100,000 population per year between 1989-9 and 2000-1.[25] Using Hospital Episode Statistics (HES) between 1998 and 2011, Bebbington and Furniss also observed an increase in the absolute number of patients with CTS and episodes of CTR, however they also noted a decrease in the use of surgery post 2008.[26]

Previous studies have used a range of methods to classify episodes of CTS and have been conducted in a number of population settings. CTS is essentially a clinical diagnosis, and in the UK, the majority of patients will first present to and be managed within primary care. Only a proportion of these patients will be referred into more specialised services and since not all surgical episodes will take place in secondary care (hospitals), as community clinics are now receiving referrals, primary care records should capture the majority of episodes. Data from a high quality source, representative of the UK population is necessary to support the planning and commissioning of services.

For peer review only - http://bmjopen.bmj.com/site/about/guidelines.xhtml

The aim of this study is therefore to provide updated estimates of the prevalence, incidence and surgical management of carpal tunnel syndrome and describe trends over a 20 year period, using data from a large national primary care database (Clinical Practice Research Datalink (CPRD).

METHODS

This was an observational study using the Clinical Practice Research Datalink (CPRD) to estimate the prevalence, incidence and surgical management of CTS from 1993 to 2013. CPRD is a live, primary care database of anonymised medical records from general practices. It holds information of over 11.3 million patients from 674 practices in the UK since 1987. 4.4 million active (alive and currently registered) patients are currently contributing information to the datalink, which equates to 6.9% of the UK population. [27] CPRD is broadly representative of the UK general population in terms of age, gender and ethnicity. [27] The CPRD has National Research Ethics Committee (NRES) approval for observational research using primary care data and as such no further permissions were required. The Independent Scientific Advisory Committee (ISAC) study protocol 14_167 was approved in September 2014. Patients were not directly involved in the design of this study, however the results will be used to inform discussions regarding further research in this field with our local Research User Group.

During clinical interactions, Read Codes are used to record signs and symptoms, treatments and therapies, investigations, occupations, diagnoses and appliances. Read codes make up a hierarchical 'thesaurus' stored by the computer. Clinical information is hence stored in a retrievable and analysable format.[28]

The study population consisted of men and women over 18 years of age. Patients were required to have 'up to standard' (which is measured at the level of the general practice) and 'acceptable patient' (which is measured at the level of the patient) data in CPRD, for two years prior to an incident episode and at the point of diagnosis for a prevalent episode. These terms are defined by CPRD. The 'up to standard' metric is based on the continuity of recorded data, including the recording of deaths, and is set at the most recent date at which practices met the quality criteria. The 'acceptable patient' metric is based on the presence of a registration status, the patient record itself and there being a valid age and gender.[27]

Prevalent and incident patients were identified by a consultation recorded using one of the Read codes listed in Table 1. Some treatment codes and in the case of in injections, linked prescription data, were included as evidence of diagnosis as per previous studies.[18] Pilot work using a local primary care database (Consultations in Primary Care Archive, CiPCA [29]) had noted that 30% of CTS cases with a treatment code (i.e. CTR or a coded carpal tunnel injection) had not initially received a diagnosis code. This means that at presentation, patients may have been attributed a more generic term such as 'hand pain' and later gone on to receive condition specific treatment. Hence, treatment codes were used to capture such cases, which would be missed when using diagnostic codes only.

Table 1 Readcodes used to define a prevalent or incident episode of carpal tunnel syndrome

Term	Read code
Carpal tunnel syndrome	F340
Injection of carpal tunnel	85BE.00
Carpal tunnel release	70560
Endoscopic carpal tunnel release	7056011
Carpal tunnel decompression	70564

For peer review only - http://bmjopen.bmj.com/site/about/guidelines.xhtml

The prevalence of individuals consulting with CTS was calculated per annum. The numerator for prevalence was the number of patients with a record of a CTS diagnosis or evidence of an episode of CTR or a carpal tunnel injection (CTI), in each calendar year. In order to determine annual incidence, the numerator was the number of patients with a record of CTS or evidence of CTR or CTI, without a prior record of these codes during a run-in-period of two years. This two year run-in period was based on expert consensus with the aim of estimating the period of time during which a new episode of CTS may develop. It was felt unlikely that a patient with ongoing bothersome symptoms would not have presented in primary care within this 2 year period. This however is an assumption made in order to define incident cases in this data set. It remains possible that patients had CTS in the community and did not present, presented in an alternative setting or indeed had a misdiagnosis / uncoded diagnosis made. CTS could present as a new episode in the contralateral wrist sometime after the initial presentation, hence it was not felt possible to define this criterion as 'no previous recorded episode'. All incidence patients were therefore required to have complete registration for this 2 calendar years prior to the event date. Pilot work in CiPCA had shown that over 9 years observed, 4% of potential incident cases were lost due to the lack of 2 years registration data required to define an incident episode.

The denominator population for calculation of prevalence was the total up-to-standard person-years contributed to CPRD by patients over the age of 18 years, for each annual period between 1993 and 2013. In order to apply the same criteria to both the numerator and denominator populations, the denominator populations for calculating incidence were also required to have registration at the mid-point of the year, two calendar years before the index year.

Episodes of carpal tunnel release (CTR) were identified using Read codes as shown in Table 2. In addition, codes used to define 're-release of carpal tunnel' and 'revision of carpal tunnel release' were included as a surgical episode (if first recorded). These terms were not included in the definition of CTS for the estimation of prevalence and incidence as they may not have indicated an episode of 'idiopathic' CTS but rather iatrogenic symptoms following previous (unsuccessful) surgery. Of note revision codes contributed 1.00% of the total surgical codes used. Results were expressed as the percentage of patients with a prevalent episode of CTS having a code of CTR in the same calendar year. Percentages were calculated based on the number of prevalent cases as opposed to incident cases as it was felt likely that patients would receive surgery in the annual period following their index consultation.

Table 2 Read codes used to define a surgical episode

Term	Read code
Carpal tunnel release	817
Re-release of carpal tunnel	16896
Endoscopic carpal tunnel release	39335
Revision of carpal tunnel release	97195
Carpal tunnel decompression	19249

Statistical methods

Age and sex specific annual prevalence and incidence were determined for each calendar year, between 1993 and 2013 and presented as n / 10,000 person years. For confidence interval calculation a Poisson distribution was used. As a sensitivity analysis, age and sex standardised annual figures of CTS prevalence and incidence for each year were also calculated, using population estimates provided by the website of the Office of National

For peer review only - http://bmjopen.bmj.com/site/about/guidelines.xhtml

Statistics.[30] Un-standardised and standardised rates were very similar, hence we report un-standardised rates as the primary outcome. The age and sex standardised estimates of the annual prevalence and incidence of CTS are shown in Supplementary Table 1.

Episodes of CTR were identified and the frequency in each calendar year expressed as a percentage of the prevalent population for the same time period. Emerging trends were described. Joinpoint regression was used to determine mean Annual Percentage Change (APC) and assess when significant changes ('joinpoints') occurred in the underlying trend for incidence, prevalence, and surgery. This method assists the exploration of the potential influence of changes in practice, although such potential associations cannot be proven.[31, 32] Models were fitted using the JOINPOINT REGRESSION PROGRAM (version 4.3.1.0) and the best fitting model chosen (up to 5 joinpoints).

RESULTS

Trends in prevalence

Table 3 presents the prevalence (crude estimates) of patients presenting in primary care with carpal tunnel syndrome between 1993 and 2013 and the demographics of the population. The denominator population for prevalence increased from 1,117,433 person years in 1993 to 3,473,094 person years in 2013. The total prevalence in 1993 was 26.03 per 10,000 person years (95% CI 25.10 – 27.00), and for 2013, 36.08 per 10,000 person years (95% CI 35.45 – 36.72). As shown in Figure 1 and corresponding Table 4, prevalence appeared to decrease between 1993 and 2000 (annual percentage change APC = -0.8%, 95% confidence interval -2.6 to 1.0). It then increased between 2000 and 2004 (APC = 7.8%, 95% CI 3.1 – 12.7) and then increased at a slower rate between 2004 and 2013 (APC = 1.1%, 95% CI 0.4 – 1.8). The female to male ratio reduced over time from 2.74 in 1993 to 1.93 in 2013. The median age of female and male patients with CTS increased from 49 and 53 years respectively in 1993 to 54 and 59 years respectively in 2013 (see Supplementary Table 2). Supplementary Table 3 and supplementary Figures 1 and 2 further illustrate the crude prevalence of CTS over time by age and gender. The prevalence of CTS appears to increase with age in the male population, whereas the prevalence in women peaks in the 50 – 59 age group.

For peer review only - http://bmjopen.bmj.com/site/about/guidelines.xhtml

Table 3 Crude prevalence of carpal tunnel syndrome (n/10,000 person years) per calendar year, as presented in UK primary care (CPRD)

Year	Number of person years	Prevalent individuals	Total crude prevalence per 10,000 person years, (95% confidence interval)	Female prevalence per 10,000 person years, (95% confidence interval)	Male prevalence per 10,000 person years, (95% confidence interval)	Female: male
1993	1117443	2909	26.03 (25.10 – 27.00)	37.52 (35.96 – 39.13)	13.69 (12.72 – 14.71)	2.74
1994	1198256	3188	26.61 (25.69 – 27.55)	37.23 (35.73 – 38.79)	15.21 (14.23 – 16.25)	2.45
1995	1286800	3343	25.98 (25.11 – 26.88)	36.64 (35.20 – 38.12)	14.58 (13.65 – 15.56)	2.51
1996	1437567	3706	25.78 (24.96 – 26.62)	36.75 (35.38 – 38.16)	14.09 (13.23 – 15.00)	2.61
1997	1681756	4190	24.91 (24.17 – 25.68)	34.87 (33.64 – 36.14)	14.34 (13.53 – 15.18)	2.43
1998	1899393	4884	25.71 (25.00 – 26.45)	36.57 (35.38 -37.79)	14.22 (13.46 – 15.01)	2.57
1999	2289158	5696	24.88 (24.24 – 25.54)	35.21 (34.14 – 36.30)	14.01 (13.32 – 14.72)	2.52
2000	2787457	6998	25.11 (24.52 – 25.70)	34.82 (33.86 – 35.81)	14.90 (14.26 – 15.57)	2.34
2001	3057458	8137	26.61 (26.04 – 27.20)	36.46 (35.52 – 37.42)	16.31 (15.67 – 16.98)	2.23
2002	3385511	9722	28.72 (28.15 – 29.29)	39.33 (38.40 – 40.28)	17.64 (17.00 – 18.29)	2.23
2003	3552908	11124	31.31 (30.73 – 31.90)	43.61 (42.66 – 44.59)	18.53 (17.90 – 19.18)	2.35
2004	3712172	12622	34.00 (33.41 – 34.60)	47.20 (46.23 – 48.19)	20.33 (19.68 – 20.99)	2.32
2005	3808183	12741	33.46 (32.88 – 34.04)	46.37 (45.42 – 47.34)	20.09 (19.45 – 20.74)	2.31
2006	3857487	12718	32.97 (32.40 – 33.55)	45.82 (44.88 – 46.78)	19.69 (19.07 – 20.33)	2.33
2007	3904068	13222	33.87 (33.29 – 34.45)	46.35 (45.41 – 47.31)	20.99 (20.35 – 21.65)	2.21
2008	3897624	14030	36.00 (35.40 – 36.60)	49.12 (48.15 – 50.11)	22.46 (21.79 – 23.14)	2.19
2009	3894989	14500	37.23 (36.60 – 37.81)	50.68 (49.69 – 51.68)	23.35 (22.68 – 24.05)	2.17
2010	3842773	14166	36.86 (36.26 – 37.48)	49.75 (48.76 – 50.75)	23.57 (22.88 – 24.27)	2.11
2011	3769676	13529	35.89 (35.29 – 36.50)	47.98 (47.00 – 48.97)	23.36 (22.67 – 24.07)	2.05
2012	3714877	13388	36.04 (35.43 – 36.66)	47.57 (46.59 – 48.56)	24.05 (23.35 – 24.78)	1.98
2013	3473094	12532	36.08 (35.45 – 36.72)	47.19 (46.18 – 48.21)	24.49 (23.75 – 25.25)	1.93

For peer review only - http://bmjopen.bmj.com/site/about/guidelines.xhtml

Table 4 Joinpoint analysis of crude prevalence

Segment	Lower Endpoint	Upper Endpoint	Annual percentage change	Lower 95th confidence interval	Upper 95th confidence interval	Test Statistic (t)	Prob > |t|
**1**	1993	2000	-0.8	-2.6	1.0	-1.0	0.3
**2**	2000	2004	7.8^	3.1	12.7	3.7	0.0
**3**	2004	2013	1.1^	0.4	1.8	3.4	0.0

Trends in incidence

Table 5 presents the annual incidence (crude estimates) for patients presenting in UK primary care with carpal tunnel syndrome between 1993 and 2013 and the demographics of the population. The denominator population for incidence, which is dependent on patients having 2 years up to standard data prior to the midpoint of the year in question, increased from 783,330 person years in 1993 to 3,015,670 person years in 2013. The crude incidence in 1993 was 20.22 per 10,000 person years (95% CI 19.24 - 21.24)), and for 2013, 27.68 per 10,000 person years (95% CI 27.09 – 28.28). As shown in Figure 2 and table 6, the results of the best fitting Joinpoint regression suggest the incidence increased between 1993 and 2000 (APC = 0.3, 95% CI -2.3 – 2.9). It then increased more quickly between 2000 and 2004 (APC = 6.9, 95% CI 0.5 – 13.7), before slowing between 2004 and 2013 (APC = 0.7. 95% CI -0.2 – 1.6). The female to male ratio reduced over time from 2.57 in 1993 to 1.88 in 2013. The median age of female and male patients were noted to increase from 50 and 51 years respectively in 1993 to 55 and 59 years respectively in 2013 (see Supplementary Table 4). Supplementary Table 5 and supplementary Figures 3 and 4 further illustrate the incidence of CTS over time by age and gender.

For peer review only - http://bmjopen.bmj.com/site/about/guidelines.xhtml

Table 5 Crude incidence of carpal tunnel syndrome (n/10,000 person years) per calendar year, as presented in UK primary care (CPRD)

**Year**	**Number of person years**	**Incident individuals**	**Total crude incidence per 10,000 person years, (95% confidence interval)**	**Female incidence per 10,000 person years, (95% confidence interval)**	**Male incidence per 10,000 person years, (95% confidence interval)**	**Female: male**
1993	783330	1584	20.22 (19.24 – 21.24))	28.72 (27.09 – 30.42)	11.17 (10.14 – 12.29)	2.57
1994	868616	1797	20.69 (19.74 – 21.67)	28.52 (26.97 – 30.13)	12.38 (11.34 – 13.69)	2.30
1995	1003593	1963	19.56 (18.70 – 20.45)	27.53 (26.12 – 29.00)	11.12 (10.20 – 12.10)	2.48
1996	1065068	2142	20.11 (19.27 – 20.98)	28.39 (27.00 – 29.84)	11.37 (10.47 – 12.33)	2.50
1997	1150299	2306	20.05 (19.24 – 20.88)	28.39 (27.05 – 29.79)	11.25 (10.39 – 12.16)	2.52
1998	1300074	2696	20.74 (19.95 – 21.52)	29.65 (28.57 – 31.22)	11.37 (10.56 – 12.23)	2.61
1999	1497673	3030	20.23 (19.52 – 20.10)	28.53 (27.35 – 29.75)	11.54 (10.77 – 12.34)	2.47
2000	1682027	3462	20.58 (19.90 – 21.28)	28.66 (27.54 – 29.81)	12.15 (11.41 – 12.93)	2.36
2001	2019596	4391	21.74 (21.10 – 22.40)	29.72 (28.68 – 30.79)	13.46 (12.74 – 14.20)	2.21
2002	2456761	5718	23.27 (22.68 – 31.78)	31.78 (30.78 – 32.79)	14.47 (13.80 – 15.17)	2.20
2003	2669111	6772	25.37 (24.77 – 25.98)	35.13 (34.14 – 36.14)	15.33 (14.67 – 16.02)	2.29
2004	2779821	7868	28.30 (27.68 – 28.94)	39.22 (38.19 – 40.27)	17.10 (16.42 – 17.81)	2.29
2005	3164506	8113	25.64 (25.08 – 26.20)	35.55 (34.63 – 36.48)	15.49 (14.88 – 16.12)	2.30
2006	3307051	8337	25.21 (24.67 – 25.76)	34.91 (34.02 – 35.82)	15.27 (14.68 – 15.89)	2.29
2007	3343009	8865	26.52 (25.97 – 27.08)	35.76 (34.86 – 36.67)	17.07 (16.45 – 17.71)	2.09
2008	3341299	9437	28.24 (27.68 – 28.82)	38.23 (37.30 – 39.17)	18.06 (17.42 – 18.72)	2.12
2009	3383196	9918	29.32 (28.74 – 29.90)	39.73 (38.79 – 50.68)	18.69 (18.04 – 19.36)	2.13
2010	3357338	9634	28.70 (28.13 – 29.27)	38.70 (37.77 – 39.64)	18.46 (17.82 – 19.13)	2.10
2011	3269296	9083	27.78 (27.21 – 28.36)	37.11 (36.19 – 38.05)	18.20 (17.54 – 18.87)	2.04
2012	3222880	9011	27.96 (27.39 – 28.54)	36.44 (35.52 – 37.88)	19.23 (18.56 – 19.93)	1.89
2013	3015670	8346	27.68 (27.09 – 28.28)	35.95 (35.01 – 36.92)	19.12 (18.43 – 19.84)	1.88

For peer review only

Table 6 Joinpoint analysis of crude incidence

Segment	Lower Endpoint	Upper Endpoint	Annual percentage change	Lower 95th confidence interval	Upper 95th confidence interval	Test Statistic (t)	Prob > |t|
1	1993	2000	0.3	-2.3	2.9	0.2	0.8
2	2000	2004	6.9^	0.5	13.7	2.3	0.0
3	2004	2013	0.7	-0.2	1.6	1.7	0.1

Trends in the percentage of patients with carpal tunnel syndrome referred and receiving surgical management

Table 7 presents the percentage of prevalent patients with a recorded episode of CTR in each calendar year between 1993 and 2013 and the demographics of this sample. The percentage of all patients with a recorded episode of CTR in 1993 was 19.35%, and for 2013, 27.41%. As shown in Figure 3 and corresponding Table 8 the percentage of patients with a coded episode of CTR increased between 1993 - 2007 (annual percentage change APC = 2.6, 95% CI 1.9 – 3.2). It then appeared to decrease between 2007 and 2013 (APC = -1.7, 95% CI -3.3 - -0.3). The median age of females and males receiving CTR were noted to increase from 53 and 55 years respectively in 1993 to 57 and 62 years respectively in 2013.

Table 7 Percentage of patients with carpal tunnel syndrome with a recorded episode of carpal tunnel release surgery per calendar year, as presented in UK primary care (CPRD)

Year	Episodes per 10,000 person years	% prevalent individuals having surgery	% prevalent females having surgery	% prevalent males having surgery	Female median age (25% - 75% Interquartile range)	Male median age (25% - 75% Interquartile range)
1993	5.04	19.35	18.78	21.03	53 (43 – 64)	55 (44 – 69)
1994	5.70	21.42	20.62	23.52	53 (43 – 68)	58 (45 – 70)
1995	6.19	23.81	23.40	24.92	53 (42 – 67)	55 (44 – 70)
1996	5.41	20.99	20.48	22.43	53 (44 – 65)	52 (40 – 65)
1997	5.70	22.89	22.14	24.81	53 (45 – 67)	56 (42 – 69)
1998	5.73	22.28	21.28	25.00	53 (44 – 65)	53 (44 – 65)
1999	6.24	25.09	24.60	26.38	54 (44 – 67)	56 (46 – 70)
2000	6.41	25.54	24.84	27.23	54 (44 – 68)	56 (45 – 69)
2001	6.88	25.87	25.95	25.68	55 (45 – 68)	58 (46 – 71)
2002	7.02	24.46	24.19	25.09	57 (46 – 71)	55 (45 – 68)
2003	8.26	26.39	25.88	27.66	56 (45 – 67)	57 (46 – 71)
2004	9.34	27.48	27.38	27.74	56 (46 – 67)	57 (47 – 68)
2005	9.70	29.00	28.31	30.65	57 (47 – 68)	58 (46 – 71)
2006	9.36	28.40	28.31	28.61	57 (47 – 68)	60 (48 – 72)
2007	9.71	28.66	28.26	29.59	56 (46 – 69)	59 (48 – 71)
2008	10.53	29.25	29.00	29.82	56 (46 – 68)	60 (49 – 72)
2009	10.92	29.32	28.73	30.66	56 (46 – 70)	61 (49 – 72)
2010	10.40	28.22	27.57	29.62	57 (47 – 71)	61 (48 – 73)
2011	9.47	26.37	26.11	26.93	57 (47 – 70)	61 (49 – 73)
2012	9.48	26.31	25.89	27.19	57 (47 – 71)	60 (49 – 73)
2013	9.89	27.41	26.47	29.30	57 (48 – 70)	62 (51 – 74)

For peer review only - http://bmjopen.bmj.com/site/about/guidelines.xhtml

Table 8 Joinpoint

Segment	Lower Endpoint	Upper Endpoint	Annual percentage change	Lower 95th confidence interval	Upper 95th confidence interval	Test Statistic (t)	Prob > |t|
**1**	1993	2007	2.6^	1.9	3.2	8.2	0.0
**2**	2007	2013	-1.7^	-3.1	-0.3	-2.6	0.0

DISCUSSION

Whilst the prevalence and incidence of CTS have increased over the study period 1993-2013, results show that episodes of surgery, increased until 2007 and declined thereafter.

Supplementary Tables 6 and 7 summarise estimates of the prevalence, incidence and sex ratios of CTS from a previous scoping review of literature pertaining to the general population, demonstrating the substantial variation in results between studies, which may partly be the results of differences in definition of CTS applied and population observed. Studies which also utilised primary care data showed a similar estimate of the incidence of CTS in a UK primary care population [18] and similarly reported an increase in incidence over time, albeit in a Dutch primary care population.[21] As described in previous studies, CTS shows a peak in prevalence and incidence in women of middle age (50-59 group, likely due to hormonal changes around the time of the menopuse)[18], whilst in the male population, the prevalence and incidence of CTS increased with age. Gelfman et al also commented that an increasing number of older people presenting with CTS had been noted over the course of their study.[20] The increase in the prevalence and incidence of CTS in the older aged male groups, may partially account for the observed decrease in the female to male ratio, over time.

The variability in the case definition of carpal tunnel syndrome was highlighted by Descatha et al 2011 who identified seven case definitions of CTS proposed for use in population based studies. Definitions included variations of: symptoms only; symptoms and examination findings; symptoms and either physical examination or electrodiagnostic results and symptoms and electrodiagnostic restults. This study showed a range in the population prevalence of CTS from 2.5% to 11%, with studies using less specific case definitions yielding higher prevalence rates.[33] Misclassification ranged between 1 and 10%.The prevalence of CTS in any given population is likely therefore to depend on the definition of CTS applied. The case definition in our study is derived from GP recorded diagnosis and treatment codes, which may have been based on clinical findings alone; those who have had further investigations and those who have received definitive condition specific treatment. Hence it utilises a pragmatic approach, across a large population that will include all patients presenting to their GP with symptoms. Our study methods do however assume that patients with symptoms will be presenting in primary care or be receiving definitive coded treatment. The study will not capture patients with chronic symptoms who are not presenting in primary care or who had a coded episode of surgery or injection.

Although Joinpoint analysis does not provide evidence for the cause of a change in observed outcomes, it highlights when a significant change in trend has taken place. Our results suggest that the annual percentage change in prevalence and incidence was highest between 2000 and 2004. A possible reason for this may be the publication of the UK Government's information technology strategy for the NHS in 1998,[34] which proposed that by 2005, the person-based electronic health record (HER), would have been fully implemented.[35] Although no direct evidence for this was found, it may be possible that with the increasing use of IT systems in primary care and attention to providing Read codes for

For peer review only - http://bmjopen.bmj.com/site/about/guidelines.xhtml

each consultation, episodes of CTS were more frequently and accurately recorded. This would not however explain the continuing increase of the incidence in CTS post 2005.

Between 2000 and 2004, the Government implemented the second phase of its 'War on Waiting,' i.e. the reduction of waiting times. For example, the maximum wait for a day-case procedure (e.g. a CTR) was reduced from 18 months to 6 months. [36] The peak in prevalence of CTS (with our definition partly based also on treatment codes, which in 2013 constituted 29.36% of prevalent patients) observed in 2004 may therefore be partly explained by the fact that patients requiring surgery were 'accumulating' between 2000 and 2004 and subsequently received definitive treatment. This effect would however not be expected to impact so heavily upon the incidence, which disregards repeat patient presentations in subsequent annual periods, unless patients with a less specific code received treatment and appeared as an incident case. The introduction of the 18 week target of time from referral to treatment in 2008 did not seem to have a similar impact on estimates of prevalence or incidence of CTS, which makes it less certain to what extent these policy changes may have influenced our results. There are likely to be further reasons behind the observed changes.

The change in trends of 2004 may also represent a change in service. The introduction of the Quality and Outcome Framework (QOF) occurred with the advent of the General Medical Services (GMS) contract in 2004. Although there has never been a musculoskeletal health domain, the importance of coding to maintain registers and evidence of outcomes in line with QOF may have influenced coding behaviour.

At the same time as QOF, Primary Care Trusts (PCT's) were given a role in commissioning services. The ability of PCT's to commission new services heralded the development of the Musculoskeletal Interface Clinics (MIC), which act as a 'one stop shop' for patients with musculoskeletal problems. A referral to this clinic from primary care may also be a reason prevalent patients with persisting symptoms stopped presenting in primary care.

These three factors (improved coding, service redevelopment and a reduction in waiting times) may all partly explain the change in incidence and prevalence of CTS between 2000 and 2004 but are unlikely to fully explain the observed trends. Further factors of potential influence may include the increasing rates of risk factors of CTS such as diabetes and obesity.[37, 38] Whilst standardising the prevalence and incidence by age and gender did not change the overall picture of the changing trends, supplementary Figure 1 suggests that the prevalence of CTS increased most obviously in the male and female over 70 year groups.

The Joinpoint analysis suggested an increase in surgical management of CTS between 1993 and 2007 (APC = 2.55), followed by a reducing trend between 2007 (95% CI 2004-2009) and the end of the study in 2013 (APC = -1.72).

Previous studies have described the epidemiology and the rates of CTR in the UK. This study provides updated data observing the presenting primary care population. Using data from the General Practice Research Database (GPRD) (forerunner to CPRD) Latinovic et al reported that 31% of patients with CTS had surgery in 2000 [18], which is similar to the 25.5% found in our study at the same time point. The small difference between the estimates may be the result of a difference in the calculation used to derive the denominator population. Audit data from one tertiary hand centre, Wild et al also showed that the rate of referrals for CTR surgery had increased over the 10 years between 1989-9 and 2000-1.[25] Furthermore, Bebbington and Furniss observed demographic population shifts in hand conditions including CTS within Hospital Episode Statistics, which record diagnoses and

For peer review only - http://bmjopen.bmj.com/site/about/guidelines.xhtml

procedures performed within NHS Hospitals in England. They used linear regression to predict future trends in hand surgery, showing that whilst absolute numbers of CTS diagnoses and CTR procedures increased between 1998 and 2011, the pre-2008 increase in CTR was significantly steeper than the post-2008 slope ($p < 0.001$).[26] This is suggestive of a decrease in the surgical management of CTS in terms of the proportion of patients with CTS having an operation, but not necessarily in the numbers of surgical episodes in absolute terms, which Bebbington and Furniss predict will have increased by 99% (95% CI 65 – 132) in 2030 compared to 2011.[26] The data from CPRD however, suggested a reduction in both real term episodes of CTR as well as the proportion of the (increasing) prevalent population receiving surgical treatment.

We may speculate regarding potential reasons for the initial increase in surgical management of CTS, for example, increased access to specialist services (e.g. community based Musculoskeletal Interface Clinics); increased litigation leading to more definitive treatments being sought, and increased patient expectations and demand, but we have no evidence for such explanations.

The decreasing trend in the use of the use of CTR post 2007 is likely to be multifactorial, however the changing structure of the NHS and its funding streams may have influenced the observed trend. Around 2007 – 2008, practice-based commissioning (PBC) was being introduced. This gave primary care notional budgets with which to purchase care for their patients with the aim of aligning clinical and financial responsibility. Restricting access to certain procedures including CTR, by implementing pre-specified criteria, was one way to help achieve this, which may have resulted in a reduction in the use of CTR.

There are a number of limitations associated with the data in this study. The accuracy of consultation data is dependent on the validity of the computerised information it uses. In a review of 212 publications which aimed to validate diagnoses recorded in GPRD data, Herrett et reported that the median proportion of cases with a confirmed diagnosis was 89% (range 24 – 100%), but the majority of publications did not present the sensitivity of a coded diagnosis, which means that information regarding the proportion of missed cases is lacking. Potential misclassification; non-attendance in primary care; variation in between GP coding and a lack of coding may all lead to an unmeasured shortfall in observed cases.[27, 39]. This study relies on the diagnosis of CTS to be correct and the subsequent coding to be precise. Whilst CTS diagnoses have not been validated, in a study comparing musculoskeletal diagnoses in four different databases, Jordan et al suggested that musculoskeletal coding in GPRD was less reliable than in its other healthcare datasets including CiPCA.[40] We took measures to reduce the effect of miscoding (e.g. including surgery and injection codes in prevalence measures, if diagnostic codes had not been used), but it is possible that results will not be entirely representative of the true prevalence and incidence of CTS.

Given the lack of clarity in the accuracy of coding and the likelihood that associated clinical encounters following a CTR were coded using a surgical code, only the first surgical code could reliably be used to indicate an episode of surgery. This is likely to have led to an underestimation of surgical episodes being identified as episodes on the contralateral hand will have been automatically discounted as they were undistinguishable. Furthermore, prevalence and incidence were similarly likely to have been underestimated as repeat presentations for the ipsilateral hand are indistinguishable from presentations in the contralateral hand.

Whilst CPRD provides a large generalizable sample, which has substantial benefits when estimating epidemiological trends, it cannot directly measure patient reported outcomes. Furthermore, surgery can be seen as a ‘gold standard’ treatment, but it does not necessarily

For peer review only - http://bmjopen.bmj.com/site/about/guidelines.xhtml

signify cure. A review of the surgical treatment of CTS reported that 70% - 90% of patients undergoing a CTR have a good outcome (definitions varied).[41] In a retrospective cohort study over a mean follow up of 13 years post-surgery, 88% of patients were either completely satisfied or very satisfied with surgery. 74% reported their symptoms had completely resolved. 1.8% (113 patients) had undergone repeat surgery. [42] There is little evidence however that CTR is an appropriate initial management option for patients presenting to primary care with mild to moderate symptoms, especially in the absence of high quality trial evidence that conservative management is ineffective.[43, 44]

Future research in this field could describe the characteristics of patients presenting with CTS in greater detail, and observe course and prognosis of CTS in primary care. It may then be possible to identify predictors of the outcome of primary care management, and potentially identify patients requiring surgery.

CONCLUSION

An increase in the incidence and prevalence of CTS is likely to lead to an increased demand on services and cost to the healthcare economy.[26] This study has demonstrated an increase in the prevalence and incidence of physician diagnosed carpal tunnel syndrome over the study period between 1993 and 2013. Rates of referral for CTS and surgical intervention have also increased over the study period, however in the later years of the study, the percent of patients receiving surgery has begun to decline.

Figures

Figure 1 Joinpoint analysis of the crude prevalence of carpal tunnel syndrome between 1993 and 2013

Figure 2 Joinpoint analysis of the crude incidence of carpal tunnel syndrome between 1993 and 2013

Figure 3 Joinpoint analysis of percentage of prevalent patients having carpal tunnel surgery

Acknowledgments
The authors wish to thank Dr Dahai Yu for his assistance with the CPRD data download.

Declaration of competing interests

"All authors have completed the ICMJE uniform disclosure form at www.icmje.org/coi_disclosure.pdf and declare: no support from any organisation for the submitted work; CB is funded by the National Institute of Health Research School for Primary Care (NIHR SPCR). The views expressed are those of the authors and not necessarily those of the NIHR, the NHS or the Department of health; DvdW is a member of PROGRESS Medical Research Council Prognosis Research Strategy (PROGRESS) Partnership (G0902393/99558); no other relationships or activities that could appear to have influenced the submitted work."

Details of contributors

CB, LC, YC and DvdW all contributed to the initial draft and subsequent revisions. CB is the guarantor of the paper. All authors had full access to all of the data and can take responsibility for the integrity of the data and the accuracy of the data analysis. CB affirms that the manuscript is an honest, accurate, and transparent account of the study being reported; that no important aspects of the study have been omitted and that any discrepancies from the study as planned have been explained.

For peer review only - http://bmjopen.bmj.com/site/about/guidelines.xhtml

Data sharing

To ensure patient privacy and confidentiality, data from the CPRD cannot be shared. Therefore, no additional data are available.

Copyright, open access and permission to reuse

The Corresponding Author has the right to grant on behalf of all authors and does grant on behalf of all authors, a worldwide licence to the Publishers and its licensees in perpetuity, in all forms, formats and media (whether known now or created in the future), to i) publish, reproduce, distribute, display and store the Contribution, ii) translate the Contribution into other languages, create adaptations, reprints, include within collections and create summaries, extracts and/or, abstracts of the Contribution, iii) create any other derivative work(s) based on the Contribution, iv) to exploit all subsidiary rights in the Contribution, v) the inclusion of electronic links from the Contribution to third party material where-ever it may be located; and, vi) licence any third party to do any or all of the above.

References

1 Alfonso C, Jann S, Massa R, et al. Diagnosis, treatment and follow-up of the carpal tunnel syndrome: A review. *Neurological Sciences* 2010;31:243-52.

2 Aroori S, Spence RA. Carpal tunnel syndrome. *Ulster Med J* 2008;77:6-17.

3 Padua L, Padua R, Lo Monaco M, et al. Multiperspective assessment of carpal tunnel syndrome: A multicenter study. *Neurology* 1999;53:1654-9.

4 Foley M, Silverstein B, Polissar N. The economic burden of carpal tunnel syndrome: long-term earnings of CTS claimants in Washington State. *Am J Ind Med* 2007;50:155-72.

5 Atroshi I, Gummesson C, Johnsson R, et al. Symptoms, disability, and quality of life in patients with carpal tunnel syndrome. *Journal of Hand Surgery - American Volume* 1999;24:398-404.

6 Jenkins PJ, Srikantharajah D, Duckworth AD, et al. Carpal tunnel syndrome: the association with occupation at a population level. *Journal of Hand Surgery-European Volume* 2013;38E:67-72 doi:10.1177/1753193412455790.

7 Middleton SD, Anakwe RE. Carpal tunnel syndrome. *BMJ* 2014;349 doi:10.1136/bmj.g6437.

8 Padua L, Coraci D, Erra C, et al. Carpal tunnel syndrome: clinical features, diagnosis, and management. *Lancet Neurol* 2016;15:1273-84 doi:S1474-4422(16)30231-9 [pii].

9 American Academy of Orthopaedic Surgeons. Clinical practice guideline on the diagnosis of carpal tunnel syndrome 2007.

10 Carpal Tunnel Syndrome. Available at: http://cks.nice.org.uk/carpal-tunnel-syndrome#!scenariorecommendation:1. Accessed 05/14, 2013.

11 Carpal Tunnel Syndrome (CTS) - The Map of Medicine. Available at: http://app.mapofmedicine.com/mom/127/page.html?department-id=8&specialty-id=1037&pathway-id=3411&page-id=8741&history=clear. Accessed 25/10/2012, 2012.

For peer review only - http://bmjopen.bmj.com/site/about/guidelines.xhtml

12 Royal College of Surgeons. Commissioning Guide: Treatment of Carpal Tunnel Syndrome 2016.

13 Middleton SD, Anakwe RE. *BMJ* 2014;349 doi:10.1136/bmj.g6437.

14 Ferry S, Pritchard T, Keenan J, et al. Estimating the prevalence of delayed median nerve conduction in the general population. *Br J Rheumatol* 1998;37:630-5.

15 de Krom MC, Knipschild PG, Kester AD, et al. Carpal tunnel syndrome: prevalence in the general population. *J Clin Epidemiol* 1992;45:373-6.

16 Atroshi I. Carpal tunnel syndrome--prevalence, electrodiagnosis and outcome instruments. *Acta Orthop Scand* 1999;70:70-1.

17 Papanicolaou GD, McCabe SJ, Firrell J. The prevalence and characteristics of nerve compression symptoms in the general population. *Journal of Hand Surgery - American Volume* 2001;26:460-6.

18 Latinovic R, Gulliford MC, Hughes RA. Incidence of common compressive neuropathies in primary care. *J Neurol Neurosurg Psychiatry* 2006;77:263-5 doi:10.1136/jnnp.2005.066696.

19 Bland JDP, Rudolfer SM. Clinical surveillance of carpal tunnel syndrome in two areas of the United Kingdom, 1991–2001. *Journal of Neurology, Neurosurgery & Psychiatry* 2003;74:1674-9 doi:10.1136/jnnp.74.12.1674.

20 Gelfman R, Melton LJ,3rd, Yawn BP, et al. Long-term trends in carpal tunnel syndrome. *Neurology* 2009;72:33-41 doi:10.1212/01.wnl.0000338533.88960.b9; 10.1212/01.wnl.0000338533.88960.b9.

21 Bongers FJ, Schellevis FG, van den Bosch WJ, et al. Carpal tunnel syndrome in general practice (1987 and 2001): incidence and the role of occupational and non-occupational factors. *Br J Gen Pract* 2007;57:36-9.

22 Atroshi I, Englund M, Turkiewicz A, et al. Incidence of physician-diagnosed carpal tunnel syndrome in the general population. *Arch Intern Med* 2011;171:943-5.

23 Jenkins PJ, Watts AC, Duckworth AD, et al. Socioeconomic deprivation and the epidemiology of carpal tunnel syndrome. *Journal of Hand Surgery-European Volume* 2012;37E:123-9 doi:10.1177/1753193411419952.

24 Nordstrom DL, DeStefano F, Vierkant RA, et al. Incidence of diagnosed carpal tunnel syndrome in a general population. *Epidemiology* 1998;9:342-5.

25 Wildin C, Dias JJ, Heras-Palou C, et al. Trends in elective hand surgery referrals from primary care. *Ann R Coll Surg Engl* 2006;88:543-6 doi:10.1308/003588406X117070.

26 Bebbington E, Furniss D. Linear regression analysis of Hospital Episode Statistics predicts a large increase in demand for elective hand surgery in England. *J Plast Reconstr Aesthet Surg* 2015;68:243-51 doi:10.1016/j.bjps.2014.10.011 [doi].

27 Herrett E, Gallagher AM, Bhaskaran K, et al. Data Resource Profile: Clinical Practice Research Datalink (CPRD). *Int J Epidemiol* 2015;44:827-36 doi:10.1093/ije/dyv098 [doi].

For peer review only

For peer review only - http://bmjopen.bmj.com/site/about/guidelines.xhtml

28 Chisholm J. The Read clinical classification. *BMJ* 1990;300:1092.

29 Jordan K, Porcheret M, Croft P. Quality of morbidity coding in general practice computerized medical records: a systematic review. *Fam Pract* 2004;21:396-412 doi:10.1093/fampra/cmh409 [doi].

30 Population Estimates. Available at: https://www.ons.gov.uk/peoplepopulationandcommunity/populationandmigration/populationestimates. Accessed 02/16, 2017.

31 National Cancer Institute. Joinpoint Regression Program - Surveillance Research Program 2017.

32 Bedson J, Chen Y, Hayward RA, et al. Trends in long-term opioid prescribing in primary care patients with musculoskeletal conditions: an observational database study. *Pain* 2016;157:1525-31 doi:10.1097/j.pain.0000000000000557.

33 Descatha A, Dale AM, Franzblau A, et al. Comparison of research case definitions for carpal tunnel syndrome. *Scand J Work Environ Health* 2011;37:298-306 doi:10.5271/sjweh.3148.

34 NHS Executive. *Information for Health: An Information Strategy for the Modern NHS 1998–2005. A National Strategy for Local Implementation* 1998.

35 Porcheret M, Hughes R, Evans D, et al. Data quality of general practice electronic health records: the impact of a program of assessments, feedback, and training. *Journal of the American Medical Informatics Association* 2004;11:78-86 doi:http://dx.doi.org/10.1197/jamia.M1362.

36 Lewis R, Appleby J. Can the English NHS meet the 18-week waiting list target?. *J R Soc Med* 2006;99:10-3.

37 Pourmemari MH, Shiri R. Diabetes as a risk factor for carpal tunnel syndrome: a systematic review and meta-analysis. *Diabetic Med* 2016;33:10-6 doi:10.1111/dme.12855.

38 Shiri R, Pourmemari MH, Falah-Hassani K, et al. The effect of excess body mass on the risk of carpal tunnel syndrome: a meta-analysis of 58 studies. *Obesity Reviews* 2015;16:1094-104 doi:10.1111/obr.12324.

39 Herrett E, Thomas SL, Schoonen WM, et al. Validation and validity of diagnoses in the General Practice Research Database: a systematic review. *Br J Clin Pharmacol* 2009;69:4-14 doi:10.1111/j.1365-2125.2009.03537.x.

40 Jordan K, Clarke AM, Symmons DP, et al. Measuring disease prevalence: a comparison of musculoskeletal disease using four general practice consultation databases. *Br J Gen Pract* 2007;57:7-14.

41 Turner A, Kimble F, Gulyás K, et al. Can the outcome of open carpal tunnel release be predicted?: a review of the literature. *ANZ J Surg* 2010;80:50-4 doi:10.1111/j.1445-2197.2009.05175.x.

42 Louie DL, Earp BE, Collins JE, et al. *The Journal of Bone & Joint Surgery* 2013;95:1067-73 doi:10.2106/JBJS.L.00903.

For peer review only - http://bmjopen.bmj.com/site/about/guidelines.xhtml

43 Marshall S, Tardif G, Ashworth N. Local corticosteroid injection for carpal tunnel syndrome. *Cochrane database of systematic reviews (Online)* 2007:001554.

44 Page MJ, Massy-Westropp N, O'Connor D, et al. Splinting for carpal tunnel syndrome. *Cochrane Database Syst Rev* 2012;7:CD010003 doi:10.1002/14651858.CD010003.

For peer review only

For peer review only - http://bmjopen.bmj.com/site/about/guidelines.xhtml

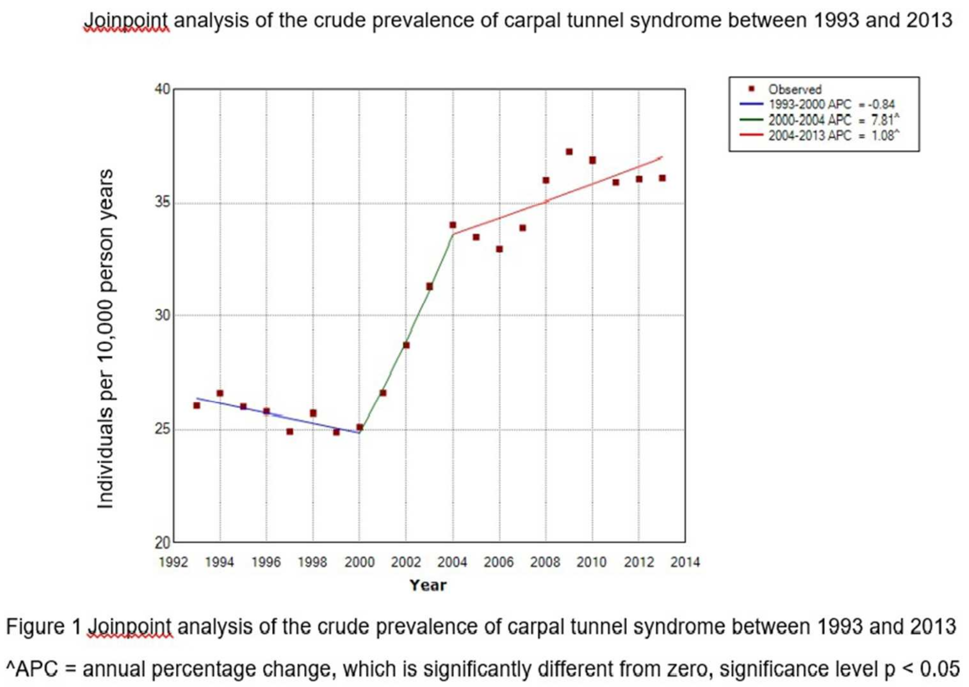

Figure 1 Joinpoint analysis of the crude prevalence of carpal tunnel syndrome between 1993 and 2013

73x50mm (300 x 300 DPI)

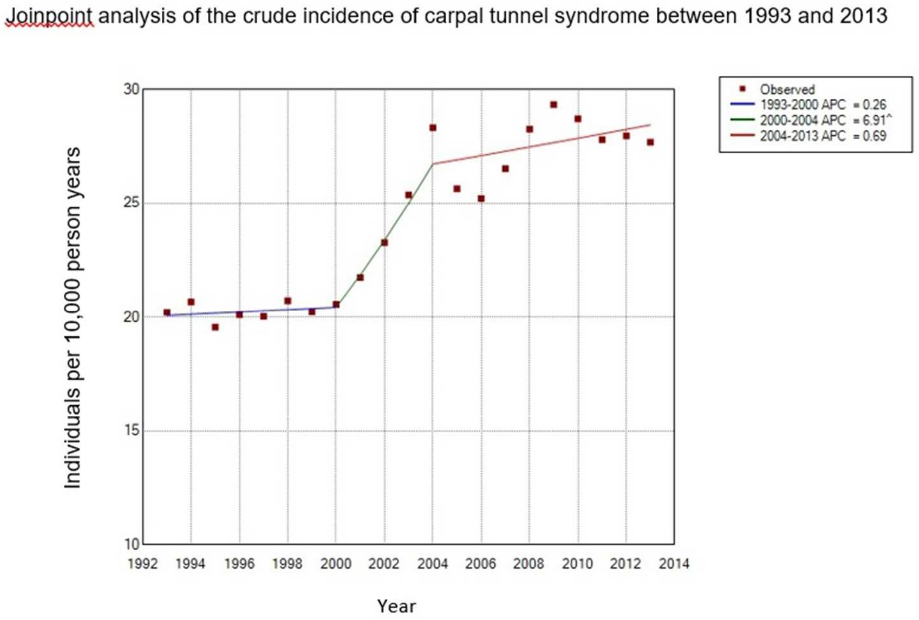

Figure 2 Joinpoint analysis of the crude incidence of carpal tunnel syndrome between 1993 and 2013

^APC = annual percentage change, which is significantly different from zero, significance level $p < 0.05$

Figure 2 Joinpoint analysis of the crude incidence of carpal tunnel syndrome between 1993 and 2013

71x52mm (300 x 300 DPI)

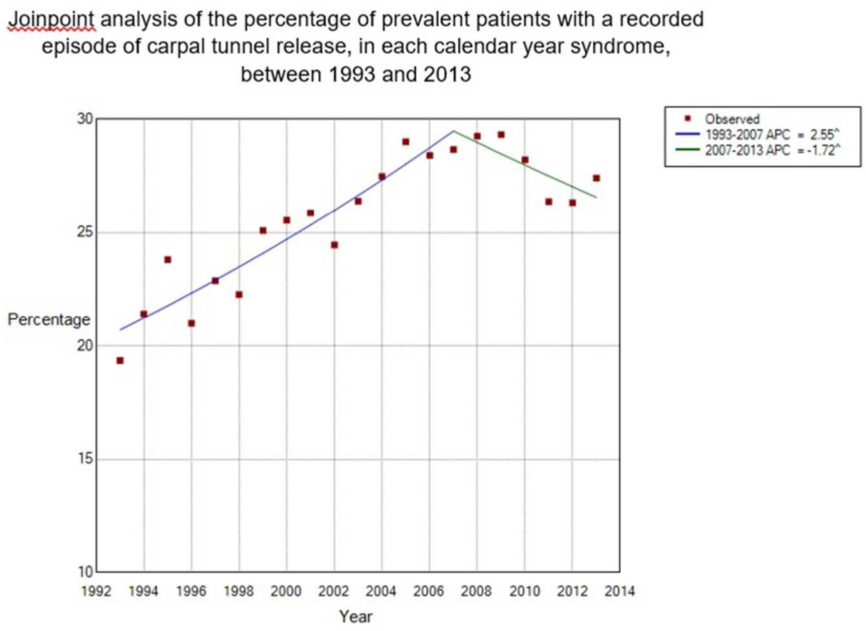

Figure 3 Joinpoint analysis of percentage of prevalent patients having carpal tunnel surgery

^APC = annual percentage change, which is significantly different from zero, significance level $p < 0.05$

Figure 3 Joinpoint analysis of the percentage of prevalent patients having carpal tunnel surgery

72x53mm (300 x 300 DPI)

ew only

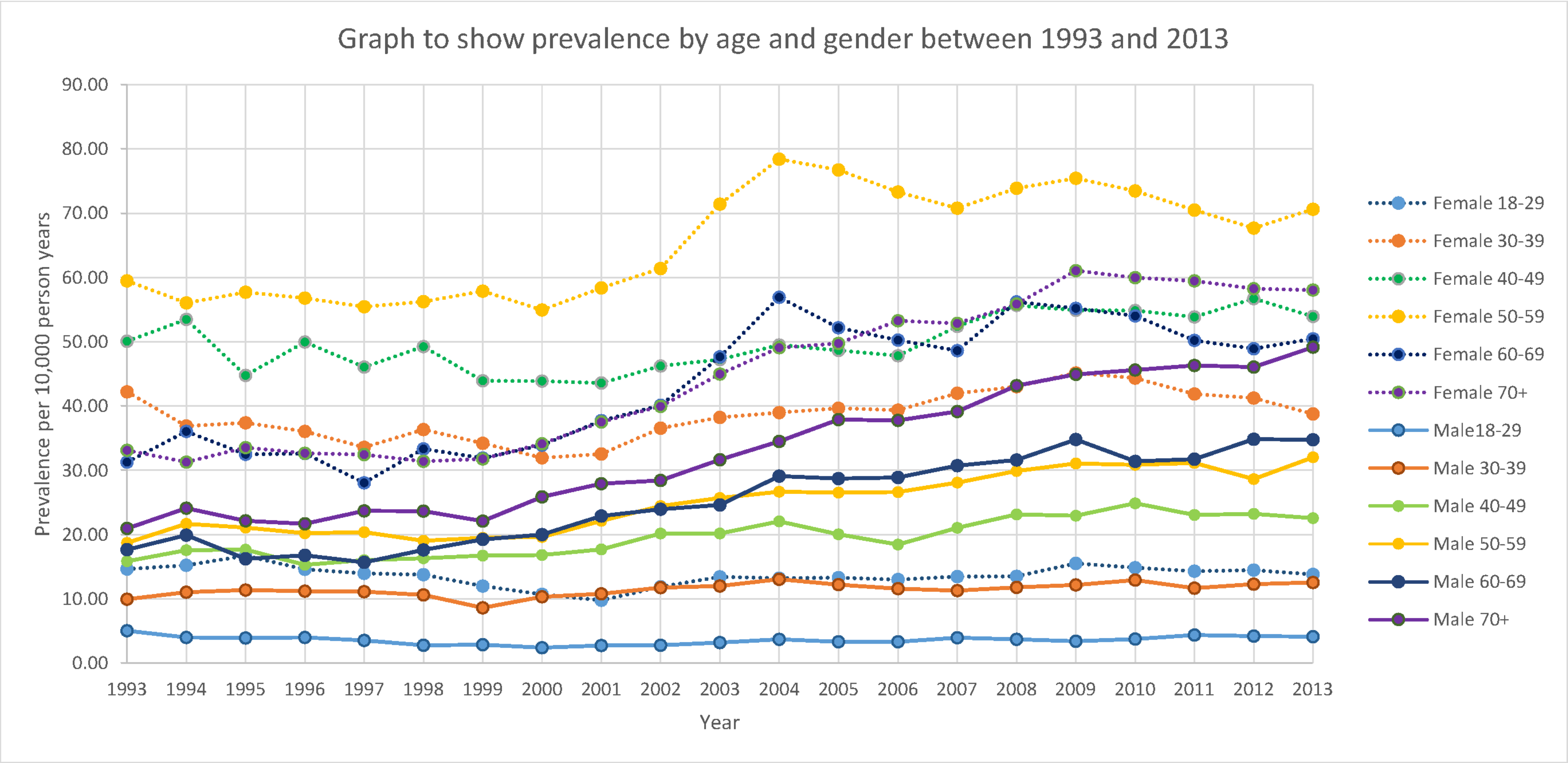
Graph to show prevalence by age and gender between 1993 and 2013
Prevalence per 10,000 person years
90.00
80.00
70.00
60.00
50.00
40.00
30.00
20.00
10.00
0.00
1993 1994 1995 1996 1997 1998 1999 2000 2001 2002 2003 2004 2005 2006 2007 2008 2009 2010 2011 2012 2013
Year
Female 18-29
Female 30-39
Female 40-49
Female 50-59
Female 60-69
Female 70+
Male18-29
Male 30-39
Male 40-49
Male 50-59
Male 60-69
Male 70+

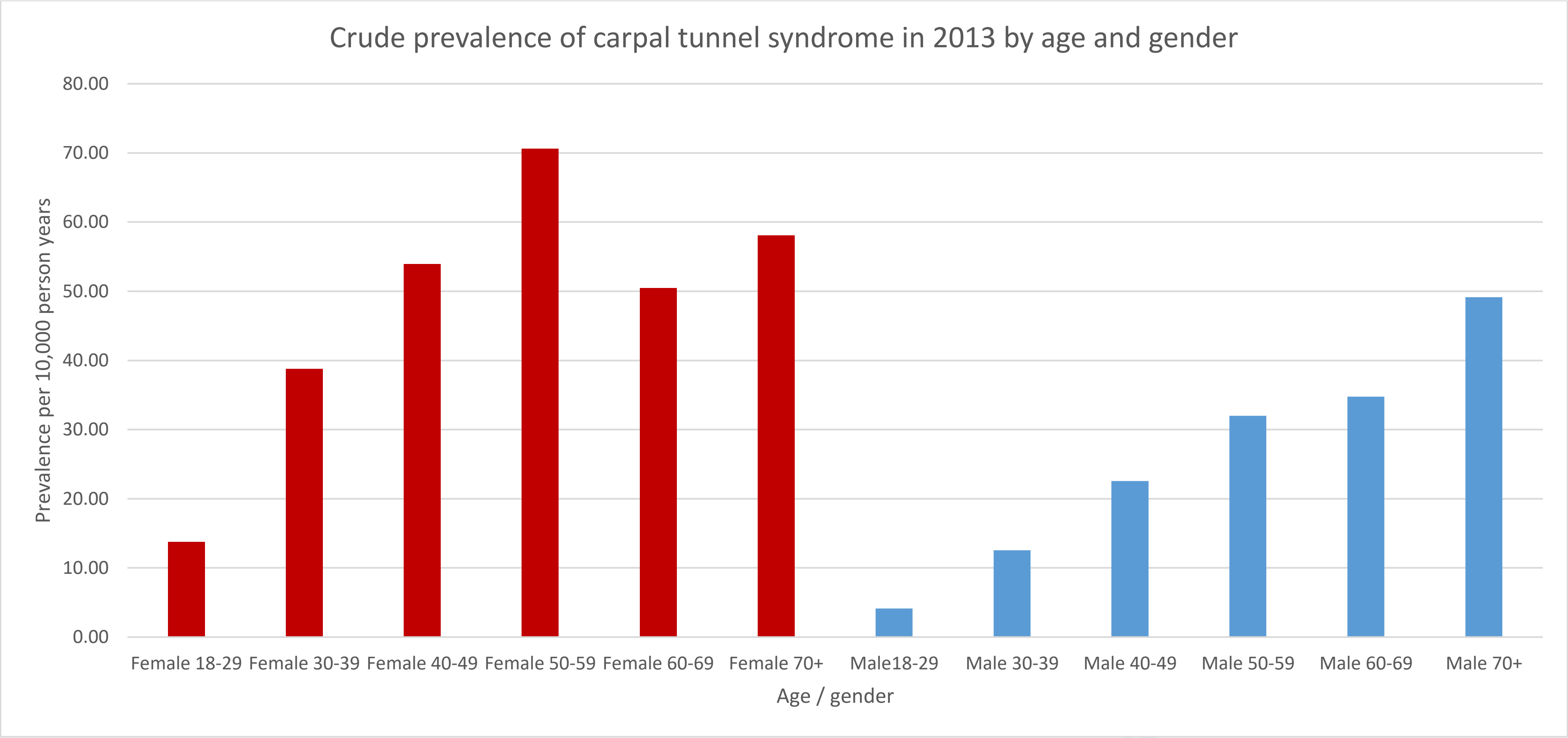

Suppl. Fig 2 Crude prevalence of carpal tunnel syndrome in 2013 by age and gender

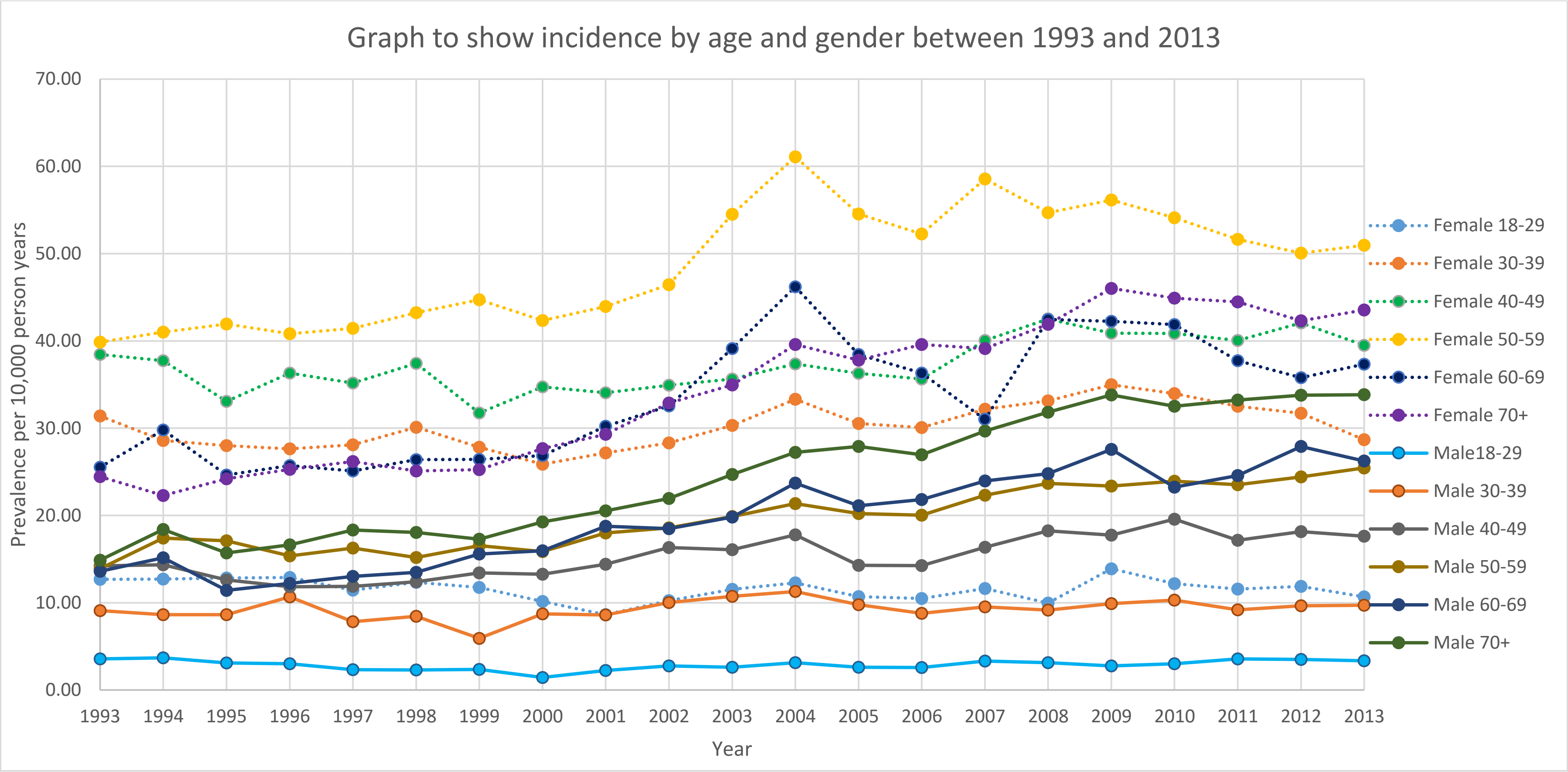

Suppl. Fig 3 Graph to show incidence by age and gender between 1993 and 2013

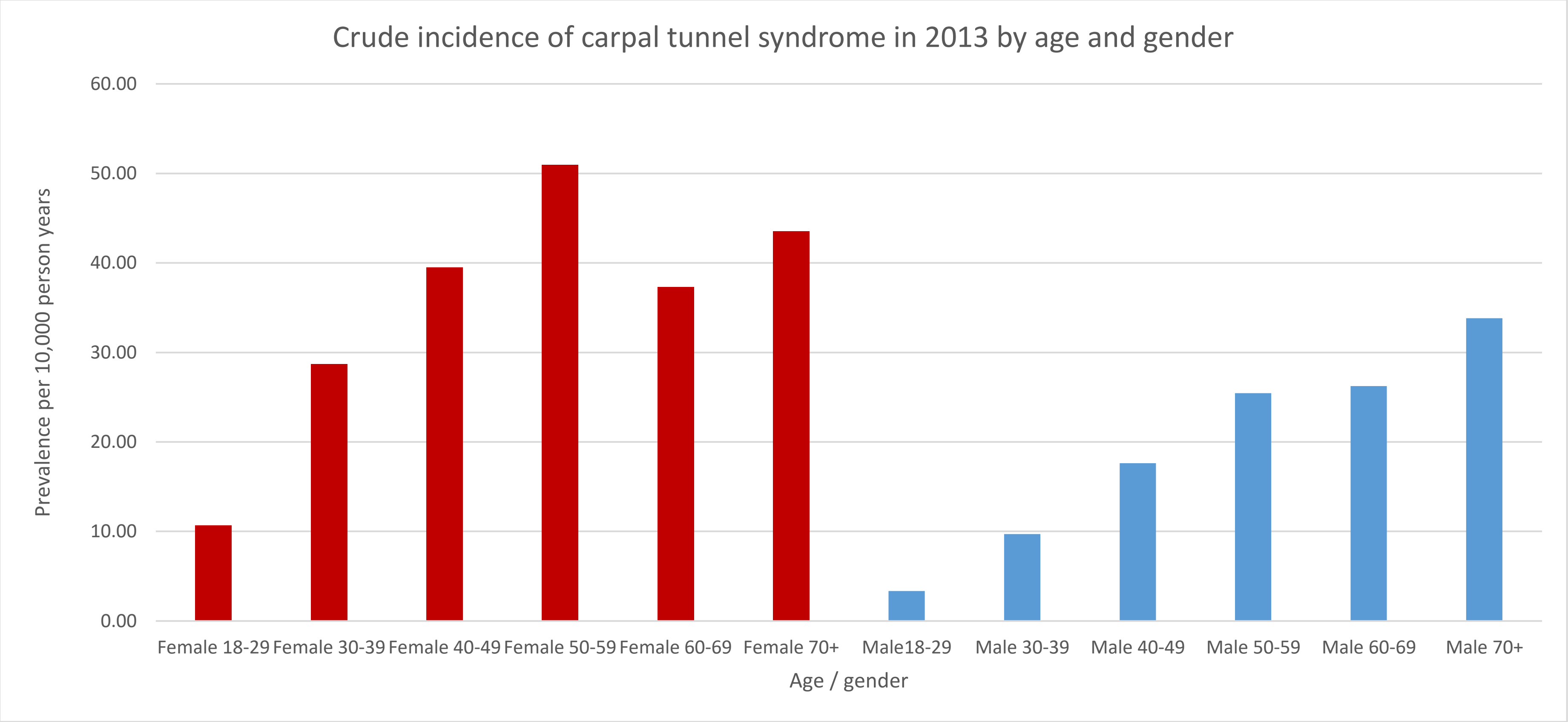

Suppl. Fig 4 Crude incidence of carpal tunnel syndrome in 2013 by age and gender

Suppl. Table 1 The age and sex standardised estimates of the annual prevalence and incidence of CTS

Year	Age sex standardised prevalence (per 10,000 person years, 95% CI)	Age sex standardised incidence (per 10,000 person years, 95% CI)
1993	26.27 (26.13 – 26.42)	19.95 (19.83 – 20.07)
1994	26.83 (26.69 – 26.98)	20.46 (20.34 – 20.59)
1995	25.90 (25.77 – 26.05)	19.20 (19.08 – 19.33)
1996	25.64 (25.50 – 25.78)	19.61 (19.49 – 19.74)
1997	24.64 (24.20 – 25.07)	19.42 (19.30 – 19.55)
1998	25.42 (25.88 – 25.56)	20.05 (19.93 – 20.18)
1999	24.57 (24.44 – 24.71)	19.51 (19.39 – 19.64)
2000	24.77 (24.63 – 24.91)	19.73 (19.61 – 19.86)
2001	26.22 (26.08 – 26.36)	20.75 (20.63 – 20.88)
2002	28.22 (28.07 – 28.37)	22.22 (22.10 – 22.36)
2003	30.81 (30.65 – 30.96)	24.28 (24.15 – 24.42)
2004	33.51 (33.35 – 33.67)	27.00 (26.86 – 27.14)
2005	32.98 (32.82 – 33.14)	24.56 (24.42 – 24.70)
2006	32.55 (32.39 – 32.70)	24.14 (24.00 – 24.27)
2007	33.48 (33.32 – 33.64)	25.52 (25.38 – 25.66)
2008	35.59 (35.43 – 25.76)	27.07 (26.92 – 27.21)
2009	36.81 (36.64 – 36.98)	28.19 (28.05 – 28.34)
2010	36.40 (36.24 – 36.66)	27.53 (27.39 – 27.68)
2011	35.28 (35.12 – 35.44)	26.59 (26.45 – 26.74)
2012	35.50 (35.34 – 35.67)	26.75 (26.61 – 26.89)
2013	35.45 (35.29 – 35.61)	26.34 (26.01 – 26.49)

For peer review only

Suppl. Table 2. Demographics of the crude prevalent population presenting with CTS in each calendar year

Year	Female median age (25% - 75% Interquartile range)	Male median age (25% - 75% Interquartile range)
**1993**	49 (38 – 62)	53 (42 – 66)
**1994**	49 (39 – 62)	53 (42 – 66)
**1995**	50 (39 – 62)	52 (41 – 64)
**1996**	50 (40 – 62)	53 (41 – 66)
**1997**	51 (40 – 62)	53 (42 – 67)
**1998**	51 (40 – 62)	54 (43 – 67)
**1999**	51 (40 – 62)	54 (44 – 66)
**2000**	52 (41 – 64)	55 (44 – 67)
**2001**	53 (42 – 65)	55 (44 – 68)
**2002**	53 (41 – 64)	55 (44 – 67)
**2003**	54 (42 – 65)	55 (44 – 68)
**2004**	55 (43 – 65)	56 (45 – 68)
**2005**	54 (43 – 65)	58 (45 – 70)
**2006**	54 (43 – 66)	58 (45 – 70)
**2007**	54 (42 – 66)	54 (42 – 66)
**2008**	54 (43 – 66)	58 (46 – 70)
**2009**	54 (43 – 67)	58 (47 – 70)
**2010**	54 (43 – 67)	57 (46 – 71)
**2011**	54 (43 – 67)	58 (47 – 71)
**2012**	54 (43 – 67)	59 (48 – 71)

For peer review only

Suppl. Table 3. Demographics of the crude incident population presenting with CTS in each calendar year

Year	Female median age (25% - 75% Interquartile range)	Male median age (25% - 75% Interquartile range)
1993	50 (39 – 63)	51 (42 – 65)
1994	50 (40 – 63)	53 (43 – 66)
1995	51 (40 – 63)	53 (42 – 64)
1996	51 (40 – 64)	52 (41 – 65)
1997	51 (40 – 64)	55 (45 – 67)
1998	51 (40 – 63)	54 (44 – 68)
1999	52 (41 – 64)	55 (45 – 67)
2000	53 (42 – 65)	55 (44 – 68)
2001	53 (42 – 66)	55 (45 – 68)
2002	54 (42 – 66)	55 (44 – 67)
2003	55 (43 – 66)	56 (45 – 68)
2004	55 (44 – 66)	57 (45 – 68)
2005	55 (43 – 66)	58 (46 – 70)
2006	55 (44 – 67)	58 (46 – 70)
2007	54 (43 – 66)	58 (47 – 70)
2008	55 (44 – 67)	58 (47 – 70)
2009	55 (44 – 67)	59 (47 – 71)

For peer review only

Suppl. Table 4 The crude prevalence of CTS by age and gender

Prevalence by age and gender	1993	1994	1995	1996	1997	1998	1999	2000	2001	2002	2003	2004	2005	2006	2007	2008	2009	2010	2011	2012	2013
Female 18-29	14.66	15.22	16.72	14.60	13.97	13.74	11.98	10.71	9.78	11.87	13.44	13.23	13.28	13.02	13.46	13.49	15.54	14.84	14.30	14.48	13.79
Female 30-39	42.21	36.87	37.37	36.04	33.57	36.34	34.20	31.94	32.55	36.57	38.21	38.97	39.67	39.34	41.98	42.99	45.15	44.34	41.87	41.24	38.78
Female 40-49	50.08	53.46	44.75	49.95	46.04	49.25	43.91	43.88	43.57	46.22	47.23	49.51	48.66	47.80	52.37	55.67	54.90	54.84	53.87	56.71	53.94
Female 50-59	59.46	56.02	57.71	56.78	55.46	56.23	57.87	54.94	58.40	61.41	71.39	78.41	76.71	73.29	70.77	73.87	75.44	73.46	70.48	67.67	70.60
Female 60-69	31.26	36.03	32.47	32.60	28.06	33.34	31.91	33.92	37.71	40.10	47.64	56.92	52.16	50.23	48.61	56.21	55.19	54.01	50.21	48.92	50.48
Female 70+	33.13	31.28	33.53	32.65	32.44	31.40	31.76	34.13	37.52	39.92	44.97	49.08	49.73	53.28	52.85	55.85	61.06	59.96	59.47	58.25	58.05
Male18-29	5.04	4.00	3.93	4.00	3.55	2.78	2.88	2.42	2.74	2.80	3.22	3.69	3.34	3.31	3.95	3.70	3.41	3.76	4.36	4.21	4.12
Male 30-39	9.95	11.02	11.36	11.19	11.11	10.60	8.61	10.32	10.78	11.75	12.00	13.06	12.23	11.57	11.30	11.78	12.17	12.94	11.67	12.29	12.55
Male 40-49	15.85	17.59	17.65	15.30	16.00	16.33	16.73	16.81	17.71	20.15	20.18	22.04	20.02	18.45	21.06	23.14	22.93	24.87	23.06	23.21	22.58
Male 50-59	18.72	21.66	21.08	20.23	20.36	19.03	19.51	19.64	22.12	24.46	25.71	26.68	26.55	26.62	28.09	29.90	31.06	30.86	31.16	28.64	32.01
Male 60-69	17.64	19.92	16.22	16.78	15.68	17.62	19.27	19.99	22.91	23.93	24.61	29.08	28.71	28.87	30.73	31.59	34.81	31.41	31.71	34.84	34.75
Male 70+	20.95	24.12	22.16	21.69	23.71	23.65	22.11	25.86	27.93	28.43	31.65	34.51	37.91	37.76	39.16	43.19	44.93	45.60	46.33	46.05	49.14

For peer review only - http://bmjopen.bmj.com/site/about/guidelines.xhtml

Suppl. Table 5 The crude incidence of CTS by age and gender

Incidence by age and gender	1993	1994	1995	1996	1997	1998	1999	2000	2001	2002	2003	2004	2005	2006	2007	2008	2009	2010	2011	2012	2013
Female 18-29	12.69	12.69	12.84	12.88	11.45	12.32	11.77	10.14	8.63	10.23	11.54	12.26	10.70	10.48	11.61	9.96	13.90	12.19	11.55	11.89	10.68
Female 30-39	31.40	28.58	28.00	27.62	28.09	30.09	27.83	25.88	27.18	28.31	30.31	33.31	30.53	30.06	32.17	33.13	34.97	33.96	32.51	31.72	28.69
Female 40-49	38.43	37.75	33.08	36.31	35.17	37.42	31.74	34.72	34.05	34.94	35.63	37.37	36.27	35.62	40.06	42.51	40.89	40.86	40.05	42.06	39.50
Female 50-59	39.86	41.02	41.93	40.82	41.44	43.24	44.70	42.33	43.94	46.44	54.52	61.11	54.56	52.25	58.55	54.70	56.14	54.10	51.62	50.07	50.97
Female 60-69	25.54	29.79	24.64	25.70	25.08	26.41	26.43	26.86	30.23	32.54	39.12	46.20	38.44	36.30	31.00	42.47	42.24	41.87	37.74	35.80	37.32
Female 70+	24.45	22.29	24.21	25.28	26.17	25.09	25.25	27.67	29.29	32.87	34.96	39.57	37.78	39.60	39.13	41.89	46.03	44.89	44.46	42.29	43.53
Male18-29	3.58	3.69	3.09	3.02	2.32	2.29	2.35	1.44	2.24	2.75	2.60	3.14	2.60	2.58	3.33	3.13	2.77	3.00	3.56	3.51	3.35
Male 30-39	9.09	8.63	8.63	10.67	7.82	8.45	5.92	8.74	8.60	10.01	10.72	11.28	9.76	8.80	9.53	9.16	9.90	10.30	9.19	9.67	9.72
Male 40-49	14.23	14.35	12.63	11.83	11.86	12.41	13.43	13.26	14.39	16.32	16.08	17.78	14.28	14.25	16.36	18.23	17.76	19.58	17.15	18.13	17.63
Male 50-59	13.90	17.40	17.11	15.36	16.26	15.17	16.54	15.87	18.00	18.59	19.87	21.36	20.23	20.02	22.30	23.69	23.38	23.91	23.51	24.40	25.45
Male 60-69	13.62	15.14	11.42	12.22	13.02	13.48	15.58	15.96	18.78	18.49	19.80	23.71	21.12	21.81	23.95	24.79	27.56	23.24	24.59	27.90	26.23

For peer review only

Male 70+	14.88	18.38	15.72	16.64	18.34	18.06	17.29	19.27	20.53	21.95	24.71	27.24	27.92	26.94	29.67	31.84	33.81	32.52	33.23	33.78	33.83

For peer review only

Suppl. Table 6 Comparison of population studies reporting the prevalence and / or incidence of carpal tunnel syndrome

Study Identifier	Study method	Definition of CTS	Comments
De Krom et al. 1992	Survey of a random age sex stratified sample of the general population taken from the population register of Maastricht between 1983 and 1985	Questionnaire based on symptoms and signs	[1]
Ferry et al. 1998	i) Cross sectional survey to estimate the point prevalence of hand symptoms (from a random sample of 1000 individuals from the UK general population, aged 18 to 75 years) and<br>ii) nerve conduction testing of a weighted sample<br>- Circa. 1998 (not stated)<br>- point prevalence determined	Based on nerve conduction studies using defined cut offs	Subjects over 54yrs had a higher prevalence than younger participants. No difference between genders was noted.[2]
Nordstrom et al. 1998	Prospective study conducted in the general population of the Marshfield Epidemiologic Study Area, Wisconsin, between 1991 and 1993	1. any diagnosis of possible, probable or definite CTS;<br>2. any diagnosis of probable or definite CTS; and<br>3. any diagnosis of possible , probable or definite CTS plus at least one of six clinical signs	A 3.5 fold increase in CTS incidence was noted compared with data from 20 years previously in the same study population[3]
Atroshi et al. 2000	Survey of a random sample of the age sex stratified general population of Southern Sweden, in 1997	Diagnosis based on clinical examination and positive electrophysiological findings	The population prevalence of symptoms was 14.4%; the prevalence of clinically and electrophysiologically confirmed CTS was 2.7% [4]
Papanicolaou, McCable & Firrell 2001	Cross-sectional study to evaluate prevalence of carpal tunnel syndrome in the General population of the United States	Katz hand diagram	After correcting for nonresponders the lowest possible estimate of CTS was 3.72% [5]

For peer review only

Mondelli, Giannini & Giacchi 2002	Prospective study of patients referred to four electrodiagnostic laboratories in the Siena area, Italy. Mean annual incidence calculated from time period 1991 to 1998	Diagnosis based on clinical history and electrodiagnostic evidence of a reduced distal conduction velocity of the median nerve (American Academy of Neurology standards)	Of the patients presenting 79.7% were women. The mean age at diagnosis was 55.0 +/- 14.4 years (range 16 to 97) [6]
Bland, Rudolfer 2003	Prospective collection of neurophysiological and clinical data of patients referred to two electromyography clinics in the UK between 1991 to 1993 and 1992 to 2001	Based on nerve conduction studies using defined cut offs	An increase in diagnosed cases was observed between the two data collection periods; attributed to referral of milder cases. Median nerve impairment was more severe in the elderly and men at all ages. [7]
Latinovic, Gulliford & Hughes 2006	Population study based in a general practice database of consulting primary care patients from 253 practices between January 1992 and 31 December 2000.	Read and Oxmis codes for carpal tunnel syndrome	Most frequent in women aged 45-54. In 2000 operative treatment was undertaken for 31% of incident CTS presentations [8]
Bonger et al. 2007	Analysis of the first and second Dutch National Survey of General Practice, conducted in 1987 and 2001	(International Classification of Primary Care) ICPC coded diagnosis	A crude increase in incidence over time was not statistically significant after subdividing by age and sex. Incidence rates were related to the job level in women, but not men [9]
Dieleman et al. 2008	Population study based in a general practice database (Integrated Primary Care Information (IPCI) database): data of consulting primary care patients in the Netherlands between 1996 and 2003	ICPC coded diagnosis	Neuropathic pain was noted to affect almost 1% of the population. Mononeuropathies and carpal tunnel syndrome were the most common causes [10]
Gelfman et al. 2009	Analysis of medical records linkage system 1981-1985 to 2000-2005 of residents of Olmsted County, Minnesota (Rochester Epidemiology Project)	Clinical coding with a sample verified by full record review	An increase in incidence was observed over the study period. An increase in young individuals seeking care for less severe CTS in the mid-1980's was followed in the 1990's by an increasing incidence in older people [11]

For peer review only

Atroshi et al. 2011	Analysis of the Skane Health Care Register (SCHR) (inhabitants presenting to public health providers), incident cases identified between 2006 - 2008	Physician diagnosed	[12]
Jenkins et al. 2012b	Prospective audit of patients referred to a regional hand service based in secondary care in Scotland between November 2004 and May 2010	Symptoms of pain or paraesthesia in the median nerve distribution and one or more of: nerve conduction deficit, thenar muscle wasting or positive Tinel or Phalen signs	Mean age of presentation 55.1years (range 22 to 96, SD 13.5 years). Mean body mass index at presentation 29.5 kg/m2 CTS more common in: females (OR 1.9, 95% CI 1.5 to 2.5) Incidence varied significantly between deprivation groups: most deprived 81/100,000 and least deprived 62/100,000 (OR 1.3, 95% CI 1.1 to 1.6) [13]
Jenkins et al. 2013	Prospective audit of patients referred to a regional hand service based in secondary care in Scotland between November 2004 and May 2010, who were employed	Clinical diagnosis based on history and examination, in most cases substantiated by nerve conduction studies	The greatest incidence as in caring and leisure occupations (197 per 100,000) and the lowest incidence was in the associate professional group (37 per 100,000) [14]
Dale 2013	Pooled analysis of six prospective studies collecting data from >50 workplaces, over variable time frames	A pooled case definition was derived to include clinical and elctrodiagnostic criteria	7.8% of 4321 subjects studied had prevalent CTS, with an additional 204 subjects meeting the CTS criteria, leading to an incidence of 2.3 cases per 100 person years [15]

1 de Krom MC, Knipschild PG, Kester AD, et al. Carpal tunnel syndrome: prevalence in the general population. *J Clin Epidemiol* 1992;45:373-6.

2 Ferry S, Pritchard T, Keenan J, et al. Estimating the prevalence of delayed median nerve conduction in the general population. *Br J Rheumatol* 1998;37:630-5.

3 Nordstrom DL, DeStefano F, Vierkant RA, et al. Incidence of diagnosed carpal tunnel syndrome in a general population. *Epidemiology* 1998;9:342-5.

For peer review only

4 Atroshi I. Carpal tunnel syndrome--prevalence, electrodiagnosis and outcome instruments. *Acta Orthop Scand* 1999;70:70-1.

5 Papanicolaou GD, McCabe SJ, Firrell J. The prevalence and characteristics of nerve compression symptoms in the general population. *Journal of Hand Surgery - American Volume* 2001;26:460-6.

6 Mondelli M, Giannini F, Giacchi M. Carpal tunnel syndrome incidence in a general population. *Neurology* 2002;58:289-94.

7 Bland JDP, Rudolfer SM. Clinical surveillance of carpal tunnel syndrome in two areas of the United Kingdom, 1991–2001. *Journal of Neurology, Neurosurgery & Psychiatry* 2003;74:1674-9 doi:10.1136/jnnp.74.12.1674.

8 Latinovic R, Gulliford MC, Hughes RA. Incidence of common compressive neuropathies in primary care. *J Neurol Neurosurg Psychiatry* 2006;77:263-5 doi:10.1136/jnnp.2005.066696.

9 Bongers FJ, Schellevis FG, van den Bosch WJ, et al. Carpal tunnel syndrome in general practice (1987 and 2001): incidence and the role of occupational and non-occupational factors. *Br J Gen Pract* 2007;57:36-9.

10 Dieleman JP, Kerklaan J, Huygen FJ, et al. Incidence rates and treatment of neuropathic pain conditions in the general population. *Pain* 2008;137:681-8 doi:10.1016/j.pain.2008.03.002 [doi].

11 Gelfman R, Melton LJ,3rd, Yawn BP, et al. Long-term trends in carpal tunnel syndrome. *Neurology* 2009;72:33-41 doi:10.1212/01.wnl.0000338533.88960.b9; 10.1212/01.wnl.0000338533.88960.b9.

12 Atroshi I, Englund M, Turkiewicz A, et al. Incidence of physician-diagnosed carpal tunnel syndrome in the general population. *Arch Intern Med* 2011;171:943-5.

13 Jenkins PJ, Watts AC, Duckworth AD, et al. Socioeconomic deprivation and the epidemiology of carpal tunnel syndrome. *Journal of Hand Surgery-European Volume* 2012;37E:123-9 doi:10.1177/1753193411419952.

14 Jenkins PJ, Srikantharajah D, Duckworth AD, et al. Carpal tunnel syndrome: the association with occupation at a population level. *Journal of Hand Surgery-European Volume* 2013;38E:67-72 doi:10.1177/1753193412455790.

15 Dale AM, Harris-Adamson C, Rempel D, et al. Prevalence and incidence of carpal tunnel syndrome in US working populations: Pooled analysis of six prospective studies. *Scandinavian Journal of Work, Environment and Health* 2013;39:495-505.

For peer review only

For peer review only

Suppl. Table 7 summary of reported prevalence and incidence by gender

Study Identifier	Country of Origin Data collection (Prevalence or Incidence)	Prevalence or Incidence per 100,000, per annum			Female / male ratio
		All	Female	Male	
De Krom et al. 1992	The Netherlands 1983 - July 1985 (Prevalence)	5700	5800	600	9.66
Atroshi et al. 2000	Sweden 1997 (Prevalence)	3800	4600	2800	1.64
Papanicolaou, McCable & Firrell 2001	United States 2001 (Prevalence)	3720			4.8
Ferry et al. 1998	United Kingdom Not stated (Incidence)	8200	6400	8200	0.78
Nordstrom et al. 1998	United States 1991 - 1993 (Incidence)	346	373	318	1.17
Mondelli, Giannini & Giacchi 2002	Italy 1991 – 1998 (mean) (Incidence)	276	506	139	3.64
Bland, Rudolfer 2003	Kent, UK 1991 - 2001 (Incidence)	105	120.5	60	2
	Huddersfield, UK		61.5	30	2

For peer review only

Latinovic, Gulliford & Hughes 2006	United Kingdom (Incidence)		192.8	87.8	2.23
Bongers et al. 2007	The Netherlands (Incidence) 1987	130	190	60	3.17
	2001	180	280	90	3.11
Dieleman et al. 2008	The Netherlands 1996 - 2003 (Incidence)	233.1			
Gelfman et al. 2009	United States (Incidence) 1981-1985	258	337	177	1.90
	2001-2005	424	542	303	1.79
Atroshi et al. 2011	Sweden 2006 - 2008 (Incidence)		428	182	2.35
Jenkins et al. 2012b	Scotland 2004 - 2010 (Incidence)	72	98	43	2.28
Jenkins et al. 2013	Scotland 2004 - 2010 (Incidence)	103			
Dale 2013	United States (Incidence)	2300			

For peer review only

STROBE Statement—checklist of items that should be included in reports of observational studies

	Item No	Recommendation	Page
**Title and abstract**	1	(*a*) Indicate the study's design with a commonly used term in the title or the abstract	1-2
		(*b*) Provide in the abstract an informative and balanced summary of what was done and what was found	1-2
**Introduction**			
Background/rationale	2	Explain the scientific background and rationale for the investigation being reported	4
Objectives	3	State specific objectives, including any prespecified hypotheses	4-5
**Methods**			
Study design	4	Present key elements of study design early in the paper	5
Setting	5	Describe the setting, locations, and relevant dates, including periods of recruitment, exposure, follow-up, and data collection	5-6
Participants	6	(*a*) *Cohort study*—Give the eligibility criteria, and the sources and methods of selection of participants. Describe methods of follow-up<br>*Case-control study*—Give the eligibility criteria, and the sources and methods of case ascertainment and control selection. Give the rationale for the choice of cases and controls<br>*Cross-sectional study*—Give the eligibility criteria, and the sources and methods of selection of participants	5-6
		(*b*) *Cohort study*—For matched studies, give matching criteria and number of exposed and unexposed<br>*Case-control study*—For matched studies, give matching criteria and the number of controls per case	
Variables	7	Clearly define all outcomes, exposures, predictors, potential confounders, and effect modifiers. Give diagnostic criteria, if applicable	5-6
Data sources/ measurement	8*	For each variable of interest, give sources of data and details of methods of assessment (measurement). Describe comparability of assessment methods if there is more than one group	na
Bias	9	Describe any efforts to address potential sources of bias	na
Study size	10	Explain how the study size was arrived at	na
Quantitative variables	11	Explain how quantitative variables were handled in the analyses. If applicable, describe which groupings were chosen and why	6-7
Statistical methods	12	(*a*) Describe all statistical methods, including those used to control for confounding	6-7
		(*b*) Describe any methods used to examine subgroups and interactions	na
		(*c*) Explain how missing data were addressed	na
		(*d*) *Cohort study*—If applicable, explain how loss to follow-up was addressed<br>*Case-control study*—If applicable, explain how matching of cases and controls was addressed<br>*Cross-sectional study*—If applicable, describe analytical methods taking account of sampling strategy	na
		(*e*) Describe any sensitivity analyses	6-7

For peer review only

Continued on next page.

**Results**			
Participants	13*	(a) Report numbers of individuals at each stage of study—eg numbers potentially eligible, examined for eligibility, confirmed eligible, included in the study, completing follow-up, and analysed	Na
		(b) Give reasons for non-participation at each stage	Na
		(c) Consider use of a flow diagram	Na
-Descriptive data	14*	(a) Give characteristics of study participants (eg demographic, clinical, social) and information on exposures and potential confounders	7-9
		(b) Indicate number of participants with missing data for each variable of interest	na
		(c) *Cohort study*—Summarise follow-up time (eg, average and total amount)	na
Outcome data	15*	*Cohort study*—Report numbers of outcome events or summary measures over time	
		Case-control study—Report numbers in each exposure category, or summary measures of exposure	
		Cross-sectional study—Report numbers of outcome events or summary measures	7-9
Main results	16	(*a*) Give unadjusted estimates and, if applicable, confounder-adjusted estimates and their precision (eg, 95% confidence interval). Make clear which confounders were adjusted for and why they were included	7-9+ tables
		(*b*) Report category boundaries when continuous variables were categorized	na
		(*c*) If relevant, consider translating estimates of relative risk into absolute risk for a meaningful time period	na
Other analyses	17	Report other analyses done—eg analyses of subgroups and interactions, and sensitivity analyses	na
**Discussion**			
Key results	18	Summarise key results with reference to study objectives	12
Limitations	19	Discuss limitations of the study, taking into account sources of potential bias or imprecision. Discuss both direction and magnitude of any potential bias	14
Interpretation	20	Give a cautious overall interpretation of results considering objectives, limitations, multiplicity of analyses, results from similar studies, and other relevant evidence	12-15
Generalisability	21	Discuss the generalisability (external validity) of the study results	14
**Other information**			
Funding	22	Give the source of funding and the role of the funders for the present study and, if applicable, for the original study on which the present article is based	15

*Give information separately for cases and controls in case-control studies and, if applicable, for exposed and unexposed groups in cohort and cross-sectional studies.

Note: An Explanation and Elaboration article discusses each checklist item and gives methodological background and published examples of transparent reporting. The STROBE checklist is best used in conjunction with this article (freely available on the Web sites of PLoS Medicine at http://www.plosmedicine.org/, Annals of Internal Medicine at http://www.annals.org/, and Epidemiology at http://www.epidem.com/). Information on the STROBE Initiative is available at www.strobe-statement.org.

For peer review only

For peer review only - http://bmjopen.bmj.com/site/about/guidelines.xhtml

BMJ Open

Trends in the prevalence, incidence and surgical management of carpal tunnel syndrome between 1993 and 2013: an observational analysis of UK primary care records

Journal:	*BMJ Open*
Manuscript ID	bmjopen-2017-020166.R2
Article Type:	Research
Date Submitted by the Author:	24-Apr-2018
Complete List of Authors:	Burton, Claire; Keele University, Arthritis Research UK Primary Care Centre, Research Institute for Primary Care & Health Sciences<br>Chen, Ying; Keele University, Arthritis Research UK Primary Care Centre, Research Institute for Primary Care & Health Sciences<br>Chesterton, Linda; Keele University, Arthritis Research UK Primary Care Centre, Research Institute for Primary Care & Health Sciences<br>van der Windt, Danielle; Keele University, Arthritis Research UK Primary Care Centre, Research Institute for Primary Care & Health Sciences
**Primary Subject Heading**:	Epidemiology
Secondary Subject Heading:	Rheumatology, General practice / Family practice
Keywords:	PRIMARY CARE, SURGERY, Musculoskeletal disorders < ORTHOPAEDIC & TRAUMA SURGERY, Hand & wrist < ORTHOPAEDIC & TRAUMA SURGERY

SCHOLARONE™
Manuscripts

only

Trends in the prevalence, incidence and surgical management of carpal tunnel syndrome between 1993 and 2013: an observational analysis of UK primary care records

Authors

Claire L. Burton[a] c.burton@keele.ac.uk GP Research Fellow

Ying Chen[a] Research[a] Research Associate in Biostatistics

Linda S. Chesterton[a] Senior Lecturer in Physiotherapy

Danielle A. van der Windt[a] Professor of Primary Care Epidemiology

[a] Arthritis Research UK Primary Care Centre, Research Institute for Primary Care & Health Sciences, Keele University, Staffordshire, England. ST5 5BG

Word count (excluding title page, abstract, references, figures and tables) 4460

Correspondence to: Dr Claire L Burton c.burton@keele.ac.uk

For peer review only - http://bmjopen.bmj.com/site/about/guidelines.xhtml

Abstract

Objectives

To describe the prevalence, incidence and surgical management of carpal tunnel syndrome (CTS), between 1993 and 2013, as recorded in the Clinical Practice Research Datalink (CPRD)

Design

We completed a series of cross-sectional epidemiological analyses to observe trends over time.

Setting

Primary care data collected between 1993 and 2013, stored in the Clinical Practice Research Datalink

Population

Individuals ≥ 18 years were selected. Prevalent and incident episodes of Carpal Tunnel syndrome (CTS) and episodes surgical intervention were identified using a list of pre-identified Read codes.

Analysis

We defined incident episodes as those with no preceding diagnostic code for CTS in the past 2 years of data. Episodes of surgery were expressed as a percentage of the prevalent population during the same calendar year. Joinpoint regression was used to determine significant changes in the underlying trend.

Results

The prevalence of CTS increased over the study period, with a particular incline between 2000 and 2004 (annual percentage change 7.81). The female to male prevalence ratio reduced over time from 2.74 in 1993 to 1.93 in 2013. The median age of females and males with CTS were noted to increase from 49 and 53 years respectively in 1993 to 54 and 59 years respectively in 2013. Incidence was also noted to increase over time. After an initial increase between 1993 and 2007, the percentage of prevalent patients with a coded surgical episode began to decrease after 2007 to 27.41% in 2013 (annual percentage change -1.7)

Conclusion

This study has demonstrated that the prevalence and incidence of carpal tunnel syndrome increased over the study period between 1993 and 2013. Rates of surgery for CTS also increased over the study period, however after 2007, the percent of patients receiving surgery showed a statistically significant decline back to the rate seen in 2004.

Key words

Carpal tunnel syndrome; primary care; epidemiology; incidence; prevalence; surgery

For peer review only - http://bmjopen.bmj.com/site/about/guidelines.xhtml

Strengths and Limitations

- Provides updated epidemiological data about a common and bothersome condition
- Set in primary care, where most cases of carpal tunnel syndrome present
- Utilises a large primary care database, generalizable to the UK population
- Relies on the correct coding and capture of episodes of carpal tunnel syndrome and carpal tunnel release surgery

For peer review only

For peer review only - http://bmjopen.bmj.com/site/about/guidelines.xhtml

INTRODUCTION

Carpal tunnel syndrome (CTS) is a chronic focal compressive neuropathy caused by the entrapment of the median nerve at the level of the carpal tunnel in the wrist.[1] CTS is the most common presentation of the entrapment neuropathies [2] and is characterised by symptoms including paraesthesia, dysesthesia, sensory loss and eventually weakness and atrophy of the thenar muscle. Symptoms are usually localised to the hand but can spread proximally to the forearm, upper arm and even shoulder.[3] Despite causing relatively localised symptoms, CTS can have substantial physical, psychological and economic consequences.[4, 5] In some cases, there may be associations with certain occupations (such as the care and leisure industry)[6] which involve the overuse of the hand and wrist as well as other physical comorbidities including: pregnancy; diabetes; hypothyroidism and obesity.[7]

The diagnosis of CTS is generally accepted to be a clinical one (based on history and examination findings) [8], although electrodiagnostic tests are commonly requested to confirm the diagnosis or differentiate among diagnoses, especially in the presence of thenar atrophy and / or persistent numbness or if surgical management is being considered.[9] The treatment of CTS is usually defined as either surgical or conservative (non-surgical). Local steroid injections and night splinting form the mainstay of primary care interventions in carpal tunnel syndrome, as indicated by national care pathways.[10, 11] Patients with moderate, severe or deteriorating symptoms following conservative treatment or sudden and severe symptoms are recommended to be referred for consideration of surgery.[12] Carpal tunnel release surgery (CTR) is routinely carried out under local anaesthetic as day surgery. Open and endoscopic approaches are used to release the flexor retinaculum.[13] Previous studies have sought to estimate the prevalence and / or incidence of CTS. Such epidemiological studies have been diverse in their approach to the populations studied and case definitions applied.[14] The reported estimates for annual prevalence range from 3720-5700 per 100,000 per year [15-17] and the reported incidence from 72 – 8200 per 100,000 per year.[6, 14, 18-23] CTS is generally accepted to be more common in women; the female to male ratio ranges between 0.78 and 9.66 [14, 15] A number of previous studies have observed the trends of prevalence or incidence over time and identified an increase [19, 20, 24], with 2005 being the latest data collection point. The most recent primary care based study in the UK utilised data from between 1992 and 2000.[18]

Episodes of CTR have also been shown to have increased, with audit data from one major tertiary UK Hand Centre suggesting that referral for CTR increased over a 10 year period from 59.7 to 112 per 100,000 population per year between 1989-9 and 2000-1.[25] Using Hospital Episode Statistics (HES) between 1998 and 2011, Bebbington and Furniss also observed an increase in the absolute number of patients with CTS and episodes of CTR, however they also noted a decrease in the use of surgery post 2008.[26]

Previous studies have used a range of methods to classify episodes of CTS and have been conducted in a number of population settings. CTS is essentially a clinical diagnosis, and in the UK, the majority of patients will first present to and be managed within primary care. Only a proportion of these patients will be referred into more specialised services and since not all surgical episodes will take place in secondary care (hospitals), as community clinics are now receiving referrals, primary care records should capture the majority of episodes. Data from a high quality source, representative of the UK population is necessary to support the planning and commissioning of services.

For peer review only - http://bmjopen.bmj.com/site/about/guidelines.xhtml

The aim of this study is therefore to provide updated estimates of the prevalence, incidence and surgical management of carpal tunnel syndrome and describe trends over a 20 year period, using data from a large national primary care database (Clinical Practice Research Datalink (CPRD).

METHODS

This was an observational study using the Clinical Practice Research Datalink (CPRD) to estimate the prevalence, incidence and surgical management of CTS from 1993 to 2013. CPRD is a live, primary care database of anonymised medical records from general practices. It holds information of over 11.3 million patients from 674 practices in the UK since 1987. 4.4 million active (alive and currently registered) patients are currently contributing information to the datalink, which equates to 6.9% of the UK population. [27] CPRD is broadly representative of the UK general population in terms of age, gender and ethnicity. [27] The CPRD has National Research Ethics Committee (NRES) approval for observational research using primary care data and as such no further permissions were required. The Independent Scientific Advisory Committee (ISAC) study protocol 14_167 was approved in September 2014.

During clinical interactions, Read Codes are used to record signs and symptoms, treatments and therapies, investigations, occupations, diagnoses and appliances. Read codes make up a hierarchical ‘thesaurus’ stored by the computer. Clinical information is hence stored in a retrievable and analysable format.[28]

The study population consisted of men and women over 18 years of age. Data was used from practices which met a data quality standard based on continuity of recorded data, and from patients who had a record including at least their registration status, age and gender. These quality standards were required to have been met for at least two years prior to an incident episode and at the point of diagnosis for a prevalent episode.[27]

Prevalent and incident patients were identified by a consultation recorded using one of the Read codes listed in Table 1. Some treatment codes and in the case of in injections, linked prescription data, were included as evidence of diagnosis as per previous studies.[18] Pilot work using a local primary care database (Consultations in Primary Care Archive, CiPCA [29]) had noted that 30% of CTS cases with a treatment code (i.e. CTR or a coded carpal tunnel injection) had not initially received a diagnosis code. This means that at presentation, patients may have been attributed a more generic term such as ‘hand pain’ and later gone on to receive condition specific treatment. Hence, treatment codes were used to capture such cases, which would be missed when using diagnostic codes only.

Table 1 Readcodes used to define a prevalent or incident episode of carpal tunnel syndrome

Term	Read code
Carpal tunnel syndrome	F340
Injection of carpal tunnel	85BE.00
Carpal tunnel release	70560
Endoscopic carpal tunnel release	7056011
Carpal tunnel decompression	70564

The prevalence of individuals consulting with CTS was calculated per annum. The numerator for prevalence was the number of patients with a record of a CTS diagnosis or evidence of an episode of CTR or a carpal tunnel injection (CTI), in each calendar year. In order to determine annual incidence, the numerator was the number of patients with a record

For peer review only - http://bmjopen.bmj.com/site/about/guidelines.xhtml

of CTS or evidence of CTR or CTI, without a prior record of these codes during a run-in-period of two years. This two year run-in period was based on expert consensus with the aim of estimating the period of time during which a new episode of CTS may develop. It was felt unlikely that a patient with ongoing bothersome symptoms would not have presented in primary care within this 2 year period. This however is an assumption made in order to define incident cases in this data set. It remains possible that patients had CTS in the community and did not present, presented in an alternative setting or indeed had a misdiagnosis / uncoded diagnosis made. CTS could present as a new episode in the contralateral wrist sometime after the initial presentation, hence it was not felt possible to define this criterion as 'no previous recorded episode'. All incidence patients were therefore required to have complete registration for this 2 calendar years prior to the event date. Pilot work in CiPCA had shown that over 9 years observed, 4% of potential incident cases were lost due to the lack of 2 years registration data required to define an incident episode.

The denominator population for calculation of prevalence was the total up-to-standard person-years contributed to CPRD by patients over the age of 18 years, for each annual period between 1993 and 2013. In order to apply the same criteria to both the numerator and denominator populations, the denominator populations for calculating incidence were also required to have registration at the mid-point of the year, two calendar years before the index year.

Episodes of carpal tunnel release (CTR) were identified using Read codes as shown in Table 2. In addition, codes used to define 're-release of carpal tunnel' and 'revision of carpal tunnel release' were included as a surgical episode (if first recorded). These terms were not included in the definition of CTS for the estimation of prevalence and incidence as they may not have indicated an episode of 'idiopathic' CTS but rather iatrogenic symptoms following previous (unsuccessful) surgery. Of note revision codes contributed 1.00% of the total surgical codes used. Results were expressed as the percentage of patients with a prevalent episode of CTS having a code of CTR in the same calendar year. Percentages were calculated based on the number of prevalent cases as opposed to incident cases as it was felt likely that patients would receive surgery in the annual period following their index consultation.

Table 2 Read codes used to define a surgical episode

Term	Read code
Carpal tunnel release	817
Re-release of carpal tunnel	16896
Endoscopic carpal tunnel release	39335
Revision of carpal tunnel release	97195
Carpal tunnel decompression	19249

Statistical methods

Age and sex specific annual prevalence and incidence were determined for each calendar year, between 1993 and 2013 and presented as n / 10,000 person years. For confidence interval calculation a Poisson distribution was used. As a sensitivity analysis, age and sex standardised annual figures of CTS prevalence and incidence for each year were also calculated, using population estimates provided by the website of the Office of National Statistics.[30] Un-standardised and standardised rates were very similar, hence we report un-standardised rates as the primary outcome. The age and sex standardised estimates of the annual prevalence and incidence of CTS are shown in Supplementary Table 1.

For peer review only - http://bmjopen.bmj.com/site/about/guidelines.xhtml

Episodes of CTR were identified and the frequency in each calendar year expressed as a percentage of the prevalent population for the same time period. Emerging trends were described. Joinpoint regression was used to determine mean Annual Percentage Change (APC) and assess when significant changes ('joinpoints') occurred in the underlying trend for incidence, prevalence, and surgery. This method assists the exploration of the potential influence of changes in practice, although such potential associations cannot be proven.[31, 32] Models were fitted using the JOINPOINT REGRESSION PROGRAM (version 4.3.1.0) and the best fitting model chosen (up to 5 joinpoints).

Patient and Public Involvement

Patients were not directly involved in the design of this study, however the results will be used to inform discussions regarding further research in this field with our local Research User Group.

RESULTS

Trends in prevalence

Table 3 presents the prevalence (crude estimates) of patients presenting in primary care with carpal tunnel syndrome between 1993 and 2013 and the demographics of the population. The denominator population for prevalence increased from 1,117,433 person years in 1993 to 3,473,094 person years in 2013. The total prevalence in 1993 was 26.03 per 10,000 person years (95% CI 25.10 – 27.00), and for 2013, 36.08 per 10,000 person years (95% CI 35.45 – 36.72). As shown in Figure 1 and corresponding Table 4, prevalence appeared to decrease between 1993 and 2000 (annual percentage change APC = -0.8%, 95% confidence interval -2.6 to 1.0). It then increased between 2000 and 2004 (APC = 7.8%, 95% CI 3.1 – 12.7) and then increased at a slower rate between 2004 and 2013 (APC = 1.1%, 95% CI 0.4 – 1.8). The female to male ratio reduced over time from 2.74 in 1993 to 1.93 in 2013. The median age of female and male patients with CTS increased from 49 and 53 years respectively in 1993 to 54 and 59 years respectively in 2013 (see Supplementary Table 2). Supplementary Table 3 and supplementary Figures 1 and 2 further illustrate the crude prevalence of CTS over time by age and gender. The prevalence of CTS appears to increase with age in the male population, whereas the prevalence in women peaks in the 50 – 59 age group, dips in the 60 – 69 years age group and then peaks once more in the 70+ year age group.

For peer review only

Table 3 Crude prevalence of carpal tunnel syndrome (n/10,000 person years) per calendar year, as presented in UK primary care (CPRD)

Year	Number of person years	Prevalent individuals	Total crude prevalence per 10,000 person years, (95% confidence interval)	Female prevalence per 10,000 person years, (95% confidence interval)	Male prevalence per 10,000 person years, (95% confidence interval)	Female: male
1993	1117443	2909	26.03 (25.10 – 27.00)	37.52 (35.96 – 39.13)	13.69 (12.72 – 14.71)	2.74
1994	1198256	3188	26.61 (25.69 – 27.55)	37.23 (35.73 – 38.79)	15.21 (14.23 – 16.25)	2.45
1995	1286800	3343	25.98 (25.11 – 26.88)	36.64 (35.20 – 38.12)	14.58 (13.65 – 15.56)	2.51
1996	1437567	3706	25.78 (24.96 – 26.62)	36.75 (35.38 – 38.16)	14.09 (13.23 – 15.00)	2.61
1997	1681756	4190	24.91 (24.17 – 25.68)	34.87 (33.64 – 36.14)	14.34 (13.53 – 15.18)	2.43
1998	1899393	4884	25.71 (25.00 – 26.45)	36.57 (35.38 -37.79)	14.22 (13.46 – 15.01)	2.57
1999	2289158	5696	24.88 (24.24 – 25.54)	35.21 (34.14 – 36.30)	14.01 (13.32 – 14.72)	2.52
2000	2787457	6998	25.11 (24.52 – 25.70)	34.82 (33.86 – 35.81)	14.90 (14.26 – 15.57)	2.34
2001	3057458	8137	26.61 (26.04 – 27.20)	36.46 (35.52 – 37.42)	16.31 (15.67 – 16.98)	2.23
2002	3385511	9722	28.72 (28.15 – 29.29)	39.33 (38.40 – 40.28)	17.64 (17.00 – 18.29)	2.23
2003	3552908	11124	31.31 (30.73 – 31.90)	43.61 (42.66 – 44.59)	18.53 (17.90 – 19.18)	2.35
2004	3712172	12622	34.00 (33.41 – 34.60)	47.20 (46.23 – 48.19)	20.33 (19.68 – 20.99)	2.32
2005	3808183	12741	33.46 (32.88 – 34.04)	46.37 (45.42 – 47.34)	20.09 (19.45 – 20.74)	2.31
2006	3857487	12718	32.97 (32.40 – 33.55)	45.82 (44.88 – 46.78)	19.69 (19.07 – 20.33)	2.33
2007	3904068	13222	33.87 (33.29 – 34.45)	46.35 (45.41 – 47.31)	20.99 (20.35 – 21.65)	2.21
2008	3897624	14030	36.00 (35.40 – 36.60)	49.12 (48.15 – 50.11)	22.46 (21.79 – 23.14)	2.19
2009	3894989	14500	37.23 (36.60 – 37.81)	50.68 (49.69 – 51.68)	23.35 (22.68 – 24.05)	2.17
2010	3842773	14166	36.86 (36.26 – 37.48)	49.75 (48.76 – 50.75)	23.57 (22.88 – 24.27)	2.11
2011	3769676	13529	35.89 (35.29 – 36.50)	47.98 (47.00 – 48.97)	23.36 (22.67 – 24.07)	2.05
2012	3714877	13388	36.04 (35.43 – 36.66)	47.57 (46.59 – 48.56)	24.05 (23.35 – 24.78)	1.98
2013	3473094	12532	36.08 (35.45 – 36.72)	47.19 (46.18 – 48.21)	24.49 (23.75 – 25.25)	1.93

For peer review only

For peer review only - http://bmjopen.bmj.com/site/about/guidelines.xhtml

Table 4 Joinpoint analysis of crude prevalence

Segment	Lower Endpoint	Upper Endpoint	Annual percentage change	Lower 95th confidence interval	Upper 95th confidence interval	Test Statistic (t)	Prob > |t|
**1**	1993	2000	-0.8	-2.6	1.0	-1.0	0.3
**2**	2000	2004	7.8^	3.1	12.7	3.7	0.0
**3**	2004	2013	1.1^	0.4	1.8	3.4	0.0

Trends in incidence

Table 5 presents the annual incidence (crude estimates) for patients presenting in UK primary care with carpal tunnel syndrome between 1993 and 2013 and the demographics of the population. The denominator population for incidence, which is dependent on patients having 2 years up to standard data prior to the midpoint of the year in question, increased from 783,330 person years in 1993 to 3,015,670 person years in 2013. The crude incidence in 1993 was 20.22 per 10,000 person years (95% CI 19.24 - 21.24)), and for 2013, 27.68 per 10,000 person years (95% CI 27.09 – 28.28). As shown in Figure 2 and table 6, the results of the best fitting Joinpoint regression suggest the incidence increased between 1993 and 2000 (APC = 0.3, 95% CI -2.3 – 2.9). It then increased more quickly between 2000 and 2004 (APC = 6.9, 95% CI 0.5 – 13.7), before slowing between 2004 and 2013 (APC = 0.7. 95% CI -0.2 – 1.6). The female to male ratio reduced over time from 2.57 in 1993 to 1.88 in 2013. The median age of female and male patients were noted to increase from 50 and 51 years respectively in 1993 to 55 and 59 years respectively in 2013 (see Supplementary Table 4). Supplementary Table 5 and supplementary Figures 3 and 4 further illustrate the incidence of CTS over time by age and gender. As with prevalence, the incidence of CTS appears to increase with age in the male population, whereas the prevalence in women peaks in the 50 – 59 age group, dip in the 60 – 69 years age group and then peak once more in the 70+ year age group.

For peer review only

Table 5 Crude incidence of carpal tunnel syndrome (n/10,000 person years) per calendar year, as presented in UK primary care (CPRD)

Year	Number of person years	Incident individuals	Total crude incidence per 10,000 person years, (95% confidence interval)	Female incidence per 10,000 person years, (95% confidence interval)	Male incidence per 10,000 person years, (95% confidence interval)	Female: male
1993	783330	1584	20.22 (19.24 – 21.24))	28.72 (27.09 – 30.42)	11.17 (10.14 – 12.29)	2.57
1994	868616	1797	20.69 (19.74 – 21.67)	28.52 (26.97 – 30.13)	12.38 (11.34 – 13.69)	2.30
1995	1003593	1963	19.56 (18.70 – 20.45)	27.53 (26.12 – 29.00)	11.12 (10.20 – 12.10)	2.48
1996	1065068	2142	20.11 (19.27 – 20.98)	28.39 (27.00 – 29.84)	11.37 (10.47 – 12.33)	2.50
1997	1150299	2306	20.05 (19.24 – 20.88)	28.39 (27.05 – 29.79)	11.25 (10.39 – 12.16)	2.52
1998	1300074	2696	20.74 (19.95 – 21.52)	29.65 (28.57 – 31.22)	11.37 (10.56 – 12.23)	2.61
1999	1497673	3030	20.23 (19.52 – 20.10)	28.53 (27.35 – 29.75)	11.54 (10.77 – 12.34)	2.47
2000	1682027	3462	20.58 (19.90 – 21.28)	28.66 (27.54 – 29.81)	12.15 (11.41 – 12.93)	2.36
2001	2019596	4391	21.74 (21.10 – 22.40)	29.72 (28.68 – 30.79)	13.46 (12.74 – 14.20)	2.21
2002	2456761	5718	23.27 (22.68 – 31.78)	31.78 (30.78 – 32.79)	14.47 (13.80 – 15.17)	2.20
2003	2669111	6772	25.37 (24.77 – 25.98)	35.13 (34.14 – 36.14)	15.33 (14.67 – 16.02)	2.29
2004	2779821	7868	28.30 (27.68 – 28.94)	39.22 (38.19 – 40.27)	17.10 (16.42 – 17.81)	2.29
2005	3164506	8113	25.64 (25.08 – 26.20)	35.55 (34.63 – 36.48)	15.49 (14.88 – 16.12)	2.30
2006	3307051	8337	25.21 (24.67 – 25.76)	34.91 (34.02 – 35.82)	15.27 (14.68 – 15.89)	2.29
2007	3343009	8865	26.52 (25.97 – 27.08)	35.76 (34.86 – 36.67)	17.07 (16.45 – 17.71)	2.09
2008	3341299	9437	28.24 (27.68 – 28.82)	38.23 (37.30 – 39.17)	18.06 (17.42 – 18.72)	2.12
2009	3383196	9918	29.32 (28.74 – 29.90)	39.73 (38.79 – 50.68)	18.69 (18.04 – 19.36)	2.13
2010	3357338	9634	28.70 (28.13 – 29.27)	38.70 (37.77 – 39.64)	18.46 (17.82 – 19.13)	2.10
2011	3269296	9083	27.78 (27.21 – 28.36)	37.11 (36.19 – 38.05)	18.20 (17.54 – 18.87)	2.04
2012	3222880	9011	27.96 (27.39 – 28.54)	36.44 (35.52 – 37.88)	19.23 (18.56 – 19.93)	1.89
2013	3015670	8346	27.68 (27.09 – 28.28)	35.95 (35.01 – 36.92)	19.12 (18.43 – 19.84)	1.88

For peer review only - http://bmjopen.bmj.com/site/about/guidelines.xhtml

Table 6 Joinpoint analysis of crude incidence

Segment	Lower Endpoint	Upper Endpoint	Annual percentage change	Lower 95th confidence interval	Upper 95th confidence interval	Test Statistic (t)	Prob > |t|
1	1993	2000	0.3	-2.3	2.9	0.2	0.8
2	2000	2004	6.9^	0.5	13.7	2.3	0.0
3	2004	2013	0.7	-0.2	1.6	1.7	0.1

Trends in the percentage of patients with carpal tunnel syndrome referred and receiving surgical management

Table 7 presents the percentage of prevalent patients with a recorded episode of CTR in each calendar year between 1993 and 2013 and the demographics of this sample. The percentage of all patients with a recorded episode of CTR in 1993 was 19.35%, and for 2013, 27.41%. As shown in Figure 3 and corresponding Table 8 the percentage of patients with a coded episode of CTR increased between 1993 - 2007 (annual percentage change APC = 2.6, 95% CI 1.9 – 3.2). It then appeared to decrease between 2007 and 2013 (APC = -1.7, 95% CI -3.3 - -0.3). The median age of females and males receiving CTR were noted to increase from 53 and 55 years respectively in 1993 to 57 and 62 years respectively in 2013.

Table 7 Percentage of patients with carpal tunnel syndrome with a recorded episode of carpal tunnel release surgery per calendar year, as presented in UK primary care (CPRD)

Year	Episodes per 10,000 person years	% prevalent individuals having surgery	% prevalent females having surgery	% prevalent males having surgery	Female median age (25% - 75% Interquartile range)	Male median age (25% - 75% Interquartile range)
1993	5.04	19.35	18.78	21.03	53 (43 – 64)	55 (44 – 69)
1994	5.70	21.42	20.62	23.52	53 (43 – 68)	58 (45 – 70)
1995	6.19	23.81	23.40	24.92	53 (42 – 67)	55 (44 – 70)
1996	5.41	20.99	20.48	22.43	53 (44 – 65)	52 (40 – 65)
1997	5.70	22.89	22.14	24.81	53 (45 – 67)	56 (42 – 69)
1998	5.73	22.28	21.28	25.00	53 (44 – 65)	53 (44 – 65)
1999	6.24	25.09	24.60	26.38	54 (44 – 67)	56 (46 – 70)
2000	6.41	25.54	24.84	27.23	54 (44 – 68)	56 (45 – 69)
2001	6.88	25.87	25.95	25.68	55 (45 – 68)	58 (46 – 71)
2002	7.02	24.46	24.19	25.09	57 (46 – 71)	55 (45 – 68)
2003	8.26	26.39	25.88	27.66	56 (45 – 67)	57 (46 – 71)
2004	9.34	27.48	27.38	27.74	56 (46 – 67)	57 (47 – 68)
2005	9.70	29.00	28.31	30.65	57 (47 – 68)	58 (46 – 71)
2006	9.36	28.40	28.31	28.61	57 (47 – 68)	60 (48 – 72)
2007	9.71	28.66	28.26	29.59	56 (46 – 69)	59 (48 – 71)
2008	10.53	29.25	29.00	29.82	56 (46 – 68)	60 (49 – 72)
2009	10.92	29.32	28.73	30.66	56 (46 – 70)	61 (49 – 72)
2010	10.40	28.22	27.57	29.62	57 (47 – 71)	61 (48 – 73)
2011	9.47	26.37	26.11	26.93	57 (47 – 70)	61 (49 – 73)
2012	9.48	26.31	25.89	27.19	57 (47 – 71)	60 (49 – 73)
2013	9.89	27.41	26.47	29.30	57 (48 – 70)	62 (51 – 74)

For peer review only - http://bmjopen.bmj.com/site/about/guidelines.xhtml

Table 8 Joinpoint

Segment	Lower Endpoint	Upper Endpoint	Annual percentage change	Lower 95th confidence interval	Upper 95th confidence interval	Test Statistic (t)	Prob > |t|
**1**	1993	2007	2.6^	1.9	3.2	8.2	0.0
**2**	2007	2013	-1.7^	-3.1	-0.3	-2.6	0.0

DISCUSSION

Whilst the prevalence and incidence of CTS have increased over the study period 1993-2013, results show that episodes of surgery, increased until 2007 and declined thereafter.

Supplementary Tables 6 and 7 summarise estimates of the prevalence, incidence and sex ratios of CTS from a previous scoping review of literature pertaining to the general population, demonstrating the substantial variation in results between studies, which may partly be the results of differences in definition of CTS applied and population observed. Studies which also utilised primary care data showed a similar estimate of the incidence of CTS in a UK primary care population [18] and similarly reported an increase in incidence over time, albeit in a Dutch primary care population.[21] As described in previous studies, CTS shows a peak in prevalence and incidence in women of middle age (50-59 group, likely due to hormonal changes around the time of the menopuse)[18], whilst in the male population, the prevalence and incidence of CTS increased with age. Gelfman et al also commented that an increasing number of older people presenting with CTS had been noted over the course of their study.[20] The increase in the prevalence and incidence of CTS in the older aged male groups, may partially account for the observed decrease in the female to male ratio, over time.

The variability in the case definition of carpal tunnel syndrome was highlighted by Descatha et al 2011 who identified seven case definitions of CTS proposed for use in population based studies. Definitions included variations of: symptoms only; symptoms and examination findings; symptoms and either physical examination or electrodiagnostic results and symptoms and electrodiagnostic restults. This study showed a range in the population prevalence of CTS from 2.5% to 11%, with studies using less specific case definitions yielding higher prevalence rates.[33] Misclassification ranged between 1 and 10%.The prevalence of CTS in any given population is likely therefore to depend on the definition of CTS applied. The case definition in our study is derived from GP recorded diagnosis and treatment codes, which may have been based on clinical findings alone; those who have had further investigations and those who have received definitive condition specific treatment. Hence it utilises a pragmatic approach, across a large population that will include all patients presenting to their GP with symptoms. Our study methods do however assume that patients with symptoms will be presenting in primary care or be receiving definitive coded treatment. The study will not capture patients with chronic symptoms who are not presenting in primary care or who had a coded episode of surgery or injection.

Although Joinpoint analysis does not provide evidence for the cause of a change in observed outcomes, it highlights when a significant change in trend has taken place. Our results suggest that the annual percentage change in prevalence and incidence was highest between 2000 and 2004. A possible reason for this may be the publication of the UK Government's information technology strategy for the NHS in 1998,[34] which proposed that by 2005, the person-based electronic health record (HER), would have been fully implemented.[35] Although no direct evidence for this was found, it may be possible that with the increasing use of IT systems in primary care and attention to providing Read codes for

For peer review only - http://bmjopen.bmj.com/site/about/guidelines.xhtml

each consultation, episodes of CTS were more frequently and accurately recorded. This would not however explain the continuing increase of the incidence in CTS post 2005.

Between 2000 and 2004, the Government implemented the second phase of its ‘War on Waiting,’ i.e. the reduction of waiting times. For example, the maximum wait for a day-case procedure (e.g. a CTR) was reduced from 18 months to 6 months. [36] The peak in prevalence of CTS (with our definition partly based also on treatment codes, which in 2013 constituted 29.36% of prevalent patients) observed in 2004 may therefore be partly explained by the fact that patients requiring surgery were ‘accumulating’ between 2000 and 2004 and subsequently received definitive treatment. This effect would however not be expected to impact so heavily upon the incidence, which disregards repeat patient presentations in subsequent annual periods, unless patients with a less specific code received treatment and appeared as an incident case. The introduction of the 18 week target of time from referral to treatment in 2008 did not seem to have a similar impact on estimates of prevalence or incidence of CTS, which makes it less certain to what extent these policy changes may have influenced our results. There are likely to be further reasons behind the observed changes.

The change in trends of 2004 may also represent a change in service. The introduction of the Quality and Outcome Framework (QOF) occurred with the advent of the General Medical Services (GMS) contract in 2004. Although there has never been a musculoskeletal health domain, the importance of coding to maintain registers and evidence of outcomes in line with QOF may have influenced coding behaviour.

At the same time as QOF, Primary Care Trusts (PCT’s) were given a role in commissioning services. The ability of PCT’s to commission new services heralded the development of the Musculoskeletal Interface Clinics (MIC), which act as a ‘one stop shop’ for patients with musculoskeletal problems. A referral to this clinic from primary care may also be a reason prevalent patients with persisting symptoms stopped presenting in primary care.

These three factors (improved coding, service redevelopment and a reduction in waiting times) may all partly explain the change in incidence and prevalence of CTS between 2000 and 2004 but are unlikely to fully explain the observed trends. Further factors of potential influence may include the increasing rates of risk factors of CTS such as diabetes and obesity.[37, 38] Whilst standardising the prevalence and incidence by age and gender did not change the overall picture of the changing trends, supplementary Figure 1 suggests that the prevalence of CTS increased most obviously in the male and female over 70 year groups.

The Joinpoint analysis suggested an increase in surgical management of CTS between 1993 and 2007 (APC = 2.55), followed by a reducing trend between 2007 (95% CI 2004-2009) and the end of the study in 2013 (APC = -1.72).

Previous studies have described the epidemiology and the rates of CTR in the UK. This study provides updated data observing the presenting primary care population. Using data from the General Practice Research Database (GPRD) (forerunner to CPRD) Latinovic et al reported that 31% of patients with CTS had surgery in 2000 [18], which is similar to the 25.5% found in our study at the same time point. The small difference between the estimates may be the result of a difference in the calculation used to derive the denominator population. Audit data from one tertiary hand centre, Wild et al also showed that the rate of referrals for CTR surgery had increased over the 10 years between 1989-9 and 2000-1.[25] Furthermore, Bebbington and Furniss observed demographic population shifts in hand conditions including CTS within Hospital Episode Statistics, which record diagnoses and

For peer review only - http://bmjopen.bmj.com/site/about/guidelines.xhtml

procedures performed within NHS Hospitals in England. They used linear regression to predict future trends in hand surgery, showing that whilst absolute numbers of CTS diagnoses and CTR procedures increased between 1998 and 2011, the pre-2008 increase in CTR was significantly steeper than the post-2008 slope ($p < 0.001$).[26] This is suggestive of a decrease in the surgical management of CTS in terms of the proportion of patients with CTS having an operation, but not necessarily in the numbers of surgical episodes in absolute terms, which Bebbington and Furniss predict will have increased by 99% (95% CI 65 – 132) in 2030 compared to 2011.[26] The data from CPRD however, suggested a reduction in both real term episodes of CTR as well as the proportion of the (increasing) prevalent population receiving surgical treatment.

We may speculate regarding potential reasons for the initial increase in surgical management of CTS, for example, increased access to specialist services (e.g. community based Musculoskeletal Interface Clinics); increased litigation leading to more definitive treatments being sought, and increased patient expectations and demand, but we have no evidence for such explanations.

The decreasing trend in the use of the use of CTR post 2007 is likely to be multifactorial, however the changing structure of the NHS and its funding streams may have influenced the observed trend. Around 2007 – 2008, practice-based commissioning (PBC) was being introduced. This gave primary care notional budgets with which to purchase care for their patients with the aim of aligning clinical and financial responsibility. Restricting access to certain procedures including CTR, by implementing pre-specified criteria, was one way to help achieve this, which may have resulted in a reduction in the use of CTR.

There are a number of limitations associated with the data in this study. The accuracy of consultation data is dependent on the validity of the computerised information it uses. In a review of 212 publications which aimed to validate diagnoses recorded in GPRD data, Herrett et reported that the median proportion of cases with a confirmed diagnosis was 89% (range 24 – 100%), but the majority of publications did not present the sensitivity of a coded diagnosis, which means that information regarding the proportion of missed cases is lacking. Potential misclassification; non-attendance in primary care; variation in between GP coding and a lack of coding may all lead to an unmeasured shortfall in observed cases.[27, 39]. This study relies on the diagnosis of CTS to be correct and the subsequent coding to be precise. Whilst CTS diagnoses have not been validated, in a study comparing musculoskeletal diagnoses in four different databases, Jordan et al suggested that musculoskeletal coding in GPRD was less reliable than in its other healthcare datasets including CiPCA.[40] We took measures to reduce the effect of miscoding (e.g. including surgery and injection codes in prevalence measures, if diagnostic codes had not been used), but it is possible that results will not be entirely representative of the true prevalence and incidence of CTS.

Given the lack of clarity in the accuracy of coding and the likelihood that associated clinical encounters following a CTR were coded using a surgical code, only the first surgical code could reliably be used to indicate an episode of surgery. This is likely to have led to an underestimation of surgical episodes being identified as episodes on the contralateral hand will have been automatically discounted as they were undistinguishable. Furthermore, prevalence and incidence were similarly likely to have been underestimated as repeat presentations for the ipsilateral hand are indistinguishable from presentations in the contralateral hand.

Whilst CPRD provides a large generalizable sample, which has substantial benefits when estimating epidemiological trends, it cannot directly measure patient reported outcomes. Furthermore, surgery can be seen as a ‘gold standard’ treatment, but it does not necessarily

For peer review only - http://bmjopen.bmj.com/site/about/guidelines.xhtml

signify cure. A review of the surgical treatment of CTS reported that 70% - 90% of patients undergoing a CTR have a good outcome (definitions varied).[41] In a retrospective cohort study over a mean follow up of 13 years post-surgery, 88% of patients were either completely satisfied or very satisfied with surgery. 74% reported their symptoms had completely resolved. 1.8% (113 patients) had undergone repeat surgery. [42] There is little evidence however that CTR is an appropriate initial management option for patients presenting to primary care with mild to moderate symptoms, especially in the absence of high quality trial evidence that conservative management is ineffective.[43, 44]

Future research in this field could describe the characteristics of patients presenting with CTS in greater detail, and observe course and prognosis of CTS in primary care. It may then be possible to identify predictors of the outcome of primary care management, and potentially identify patients requiring surgery.

CONCLUSION

An increase in the incidence and prevalence of CTS is likely to lead to an increased demand on services and cost to the healthcare economy.[26] This study has demonstrated an increase in the prevalence and incidence of physician diagnosed carpal tunnel syndrome over the study period between 1993 and 2013. Rates of referral for CTS and surgical intervention have also increased over the study period, however in the later years of the study, the percent of patients receiving surgery has begun to decline.

Figures

Figure 1 Joinpoint analysis of the crude prevalence of carpal tunnel syndrome between 1993 and 2013

Figure 2 Joinpoint analysis of the crude incidence of carpal tunnel syndrome between 1993 and 2013

Figure 3 Joinpoint analysis of percentage of prevalent patients having carpal tunnel surgery

Acknowledgments
The authors wish to thank Dr Dahai Yu for his assistance with the CPRD data download.

Declaration of competing interests

"All authors have completed the ICMJE uniform disclosure form at www.icmje.org/coi_disclosure.pdf and declare: no support from any organisation for the submitted work

Funding statement

CB is funded by the National Institute of Health Research School for Primary Care (NIHR SPCR). The views expressed are those of the authors and not necessarily those of the NIHR, the NHS or the Department of health; DvdW is a member of PROGRESS Medical Research Council Prognosis Research Strategy (PROGRESS) Partnership (G0902393/99558); no other relationships or activities that could appear to have influenced the submitted work."

Details of contributors

CB, LC, YC and DvdW all contributed to the initial draft and subsequent revisions. CB is the guarantor of the paper. All authors had full access to all of the data and can take responsibility for the integrity of the data and the accuracy of the data analysis. CB affirms

For peer review only - http://bmjopen.bmj.com/site/about/guidelines.xhtml

that the manuscript is an honest, accurate, and transparent account of the study being reported; that no important aspects of the study have been omitted and that any discrepancies from the study as planned have been explained.

Data sharing

To ensure patient privacy and confidentiality, data from the CPRD cannot be shared. Therefore, no additional data are available.

Copyright, open access and permission to reuse

The Corresponding Author has the right to grant on behalf of all authors and does grant on behalf of all authors, *a worldwide licence* to the Publishers and its licensees in perpetuity, in all forms, formats and media (whether known now or created in the future), to i) publish, reproduce, distribute, display and store the Contribution, ii) translate the Contribution into other languages, create adaptations, reprints, include within collections and create summaries, extracts and/or, abstracts of the Contribution, iii) create any other derivative work(s) based on the Contribution, iv) to exploit all subsidiary rights in the Contribution, v) the inclusion of electronic links from the Contribution to third party material where-ever it may be located; and, vi) licence any third party to do any or all of the above.

References

1 Alfonso C, Jann S, Massa R, et al. Diagnosis, treatment and follow-up of the carpal tunnel syndrome: A review. *Neurological Sciences* 2010;31:243-52.

2 Aroori S, Spence RA. Carpal tunnel syndrome. *Ulster Med J* 2008;77:6-17.

3 Padua L, Padua R, Lo Monaco M, et al. Multiperspective assessment of carpal tunnel syndrome: A multicenter study. *Neurology* 1999;53:1654-9.

4 Foley M, Silverstein B, Polissar N. The economic burden of carpal tunnel syndrome: long-term earnings of CTS claimants in Washington State. *Am J Ind Med* 2007;50:155-72.

5 Atroshi I, Gummesson C, Johnsson R, et al. Symptoms, disability, and quality of life in patients with carpal tunnel syndrome. *Journal of Hand Surgery - American Volume* 1999;24:398-404.

6 Jenkins PJ, Srikantharajah D, Duckworth AD, et al. Carpal tunnel syndrome: the association with occupation at a population level. *Journal of Hand Surgery-European Volume* 2013;38E:67-72 doi:10.1177/1753193412455790.

7 Middleton SD, Anakwe RE. Carpal tunnel syndrome. *BMJ* 2014;349 doi:10.1136/bmj.g6437.

8 Padua L, Coraci D, Erra C, et al. Carpal tunnel syndrome: clinical features, diagnosis, and management. *Lancet Neurol* 2016;15:1273-84 doi:S1474-4422(16)30231-9 [pii].

9 American Academy of Orthopaedic Surgeons. Clinical practice guideline on the diagnosis of carpal tunnel syndrome 2007.

10 Carpal Tunnel Syndrome. Available at: http://cks.nice.org.uk/carpal-tunnel-syndrome#!scenariorecommendation:1. Accessed 05/14, 2013.

For peer review only - http://bmjopen.bmj.com/site/about/guidelines.xhtml

11 Carpal Tunnel Syndrome (CTS) - The Map of Medicine. Available at: http://app.mapofmedicine.com/mom/127/page.html?department-id=8&specialty-id=1037&pathway-id=3411&page-id=8741&history=clear. Accessed 25/10/2012, 2012.

12 Royal College of Surgeons. Commissioning Guide: Treatment of Carpal Tunnel Syndrome 2016.

13 Middleton SD, Anakwe RE. *BMJ* 2014;349 doi:10.1136/bmj.g6437.

14 Ferry S, Pritchard T, Keenan J, et al. Estimating the prevalence of delayed median nerve conduction in the general population. *Br J Rheumatol* 1998;37:630-5.

15 de Krom MC, Knipschild PG, Kester AD, et al. Carpal tunnel syndrome: prevalence in the general population. *J Clin Epidemiol* 1992;45:373-6.

16 Atroshi I. Carpal tunnel syndrome--prevalence, electrodiagnosis and outcome instruments. *Acta Orthop Scand* 1999;70:70-1.

17 Papanicolaou GD, McCabe SJ, Firrell J. The prevalence and characteristics of nerve compression symptoms in the general population. *Journal of Hand Surgery - American Volume* 2001;26:460-6.

18 Latinovic R, Gulliford MC, Hughes RA. Incidence of common compressive neuropathies in primary care. *J Neurol Neurosurg Psychiatry* 2006;77:263-5 doi:10.1136/jnnp.2005.066696.

19 Bland JDP, Rudolfer SM. Clinical surveillance of carpal tunnel syndrome in two areas of the United Kingdom, 1991–2001. *Journal of Neurology, Neurosurgery & Psychiatry* 2003;74:1674-9 doi:10.1136/jnnp.74.12.1674.

20 Gelfman R, Melton LJ,3rd, Yawn BP, et al. Long-term trends in carpal tunnel syndrome. *Neurology* 2009;72:33-41 doi:10.1212/01.wnl.0000338533.88960.b9; 10.1212/01.wnl.0000338533.88960.b9.

21 Bongers FJ, Schellevis FG, van den Bosch WJ, et al. Carpal tunnel syndrome in general practice (1987 and 2001): incidence and the role of occupational and non-occupational factors. *Br J Gen Pract* 2007;57:36-9.

22 Atroshi I, Englund M, Turkiewicz A, et al. Incidence of physician-diagnosed carpal tunnel syndrome in the general population. *Arch Intern Med* 2011;171:943-5.

23 Jenkins PJ, Watts AC, Duckworth AD, et al. Socioeconomic deprivation and the epidemiology of carpal tunnel syndrome. *Journal of Hand Surgery-European Volume* 2012;37E:123-9 doi:10.1177/1753193411419952.

24 Nordstrom DL, DeStefano F, Vierkant RA, et al. Incidence of diagnosed carpal tunnel syndrome in a general population. *Epidemiology* 1998;9:342-5.

25 Wildin C, Dias JJ, Heras-Palou C, et al. Trends in elective hand surgery referrals from primary care. *Ann R Coll Surg Engl* 2006;88:543-6 doi:10.1308/003588406X117070.

For peer review only - http://bmjopen.bmj.com/site/about/guidelines.xhtml

26 Bebbington E, Furniss D. Linear regression analysis of Hospital Episode Statistics predicts a large increase in demand for elective hand surgery in England. *J Plast Reconstr Aesthet Surg* 2015;68:243-51 doi:10.1016/j.bjps.2014.10.011 [doi].

27 Herrett E, Gallagher AM, Bhaskaran K, et al. Data Resource Profile: Clinical Practice Research Datalink (CPRD). *Int J Epidemiol* 2015;44:827-36 doi:10.1093/ije/dyv098 [doi].

28 Chisholm J. The Read clinical classification. *BMJ* 1990;300:1092.

29 Jordan K, Porcheret M, Croft P. Quality of morbidity coding in general practice computerized medical records: a systematic review. *Fam Pract* 2004;21:396-412 doi:10.1093/fampra/cmh409 [doi].

30 Population Estimates. Available at: https://www.ons.gov.uk/peoplepopulationandcommunity/populationandmigration/populationestimates. Accessed 02/16, 2017.

31 National Cancer Institute. Joinpoint Regression Program - Surveillance Research Program 2017.

32 Bedson J, Chen Y, Hayward RA, et al. Trends in long-term opioid prescribing in primary care patients with musculoskeletal conditions: an observational database study. *Pain* 2016;157:1525-31 doi:10.1097/j.pain.0000000000000557.

33 Descatha A, Dale AM, Franzblau A, et al. Comparison of research case definitions for carpal tunnel syndrome. *Scand J Work Environ Health* 2011;37:298-306 doi:10.5271/sjweh.3148.

34 NHS Executive. *Information for Health: An Information Strategy for the Modern NHS 1998–2005. A National Strategy for Local Implementation* 1998.

35 Porcheret M, Hughes R, Evans D, et al. Data quality of general practice electronic health records: the impact of a program of assessments, feedback, and training. *Journal of the American Medical Informatics Association* 2004;11:78-86 doi:http://dx.doi.org/10.1197/jamia.M1362.

36 Lewis R, Appleby J. Can the English NHS meet the 18-week waiting list target?. *J R Soc Med* 2006;99:10-3.

37 Pourmemari MH, Shiri R. Diabetes as a risk factor for carpal tunnel syndrome: a systematic review and meta-analysis. *Diabetic Med* 2016;33:10-6 doi:10.1111/dme.12855.

38 Shiri R, Pourmemari MH, Falah-Hassani K, et al. The effect of excess body mass on the risk of carpal tunnel syndrome: a meta-analysis of 58 studies. *Obesity Reviews* 2015;16:1094-104 doi:10.1111/obr.12324.

39 Herrett E, Thomas SL, Schoonen WM, et al. Validation and validity of diagnoses in the General Practice Research Database: a systematic review. *Br J Clin Pharmacol* 2009;69:4-14 doi:10.1111/j.1365-2125.2009.03537.x.

40 Jordan K, Clarke AM, Symmons DP, et al. Measuring disease prevalence: a comparison of musculoskeletal disease using four general practice consultation databases. *Br J Gen Pract* 2007;57:7-14.

For peer review only - http://bmjopen.bmj.com/site/about/guidelines.xhtml

41 Turner A, Kimble F, Gulyás K, et al. Can the outcome of open carpal tunnel release be predicted?: a review of the literature. *ANZ J Surg* 2010;80:50-4 doi:10.1111/j.1445-2197.2009.05175.x.

42 Louie DL, Earp BE, Collins JE, et al. *The Journal of Bone & Joint Surgery* 2013;95:1067-73 doi:10.2106/JBJS.L.00903.

43 Marshall S, Tardif G, Ashworth N. Local corticosteroid injection for carpal tunnel syndrome. *Cochrane database of systematic reviews (Online)* 2007:001554.

44 Page MJ, Massy-Westropp N, O'Connor D, et al. Splinting for carpal tunnel syndrome. *Cochrane Database Syst Rev* 2012;7:CD010003 doi:10.1002/14651858.CD010003.

For peer review only

For peer review only - http://bmjopen.bmj.com/site/about/guidelines.xhtml

Joinpoint analysis of the crude prevalence of carpal tunnel syndrome between 1993 and 2013

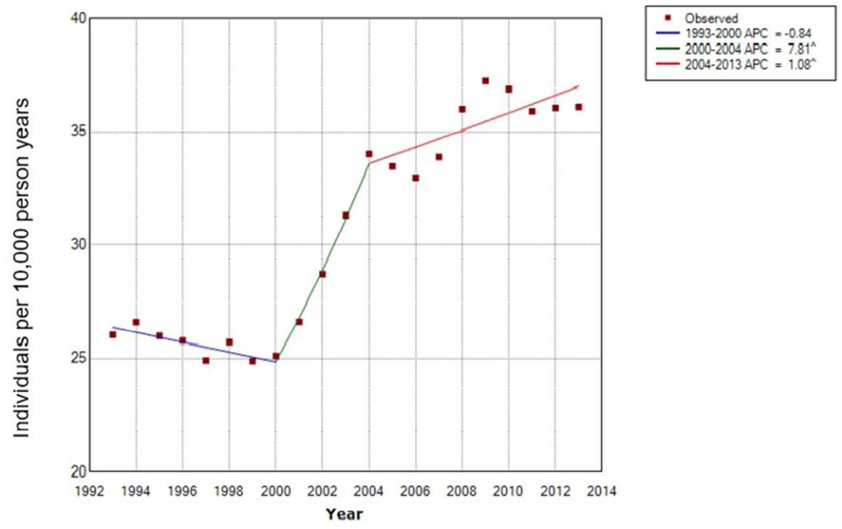

Figure 1 Joinpoint analysis of the crude prevalence of carpal tunnel syndrome between 1993 and 2013

^APC = annual percentage change, which is significantly different from zero, significance level $p < 0.05$

Figure 1 Joinpoint analysis of the crude prevalence of carpal tunnel syndrome between 1993 and 2013

73x50mm (300 x 300 DPI)

ew only

Joinpoint analysis of the crude incidence of carpal tunnel syndrome between 1993 and 2013

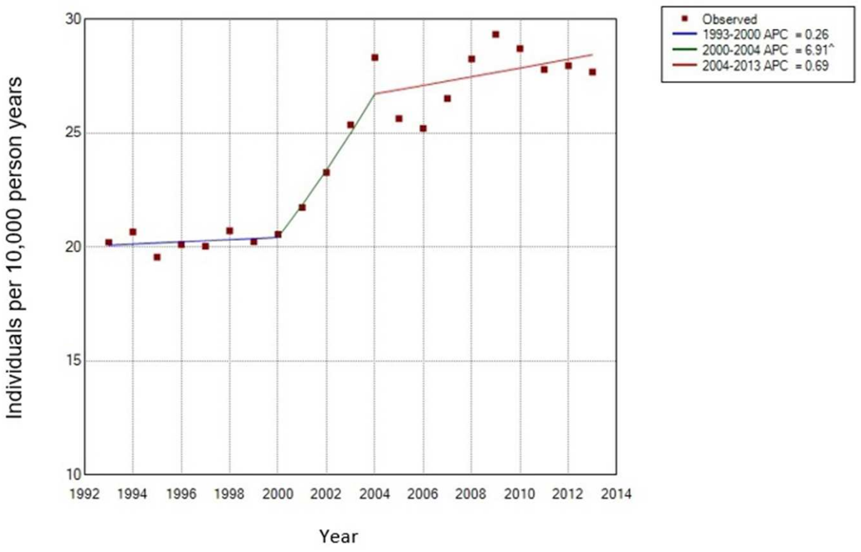

Figure 2 Joinpoint analysis of the crude incidence of carpal tunnel syndrome between 1993 and 2013

^APC = annual percentage change, which is significantly different from zero, significance level $p < 0.05$

Figure 2 Joinpoint analysis of the crude incidence of carpal tunnel syndrome between 1993 and 2013

71x52mm (300 x 300 DPI)

ew only

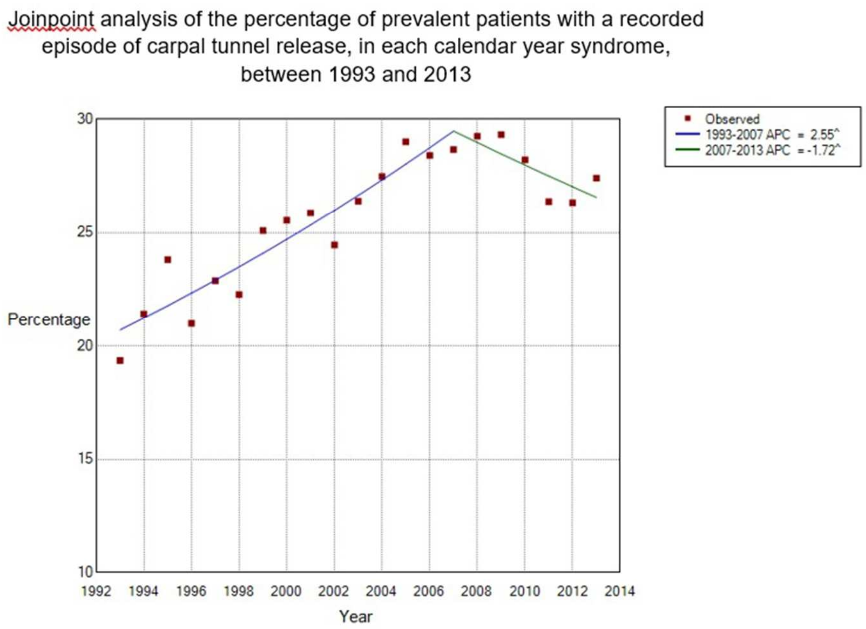

Figure 3 Joinpoint analysis of percentage of prevalent patients having carpal tunnel surgery

^APC = annual percentage change, which is significantly different from zero, significance level $p < 0.05$

Figure 3 Joinpoint analysis of the percentage of prevalent patients having carpal tunnel surgery

72x53mm (300 x 300 DPI)

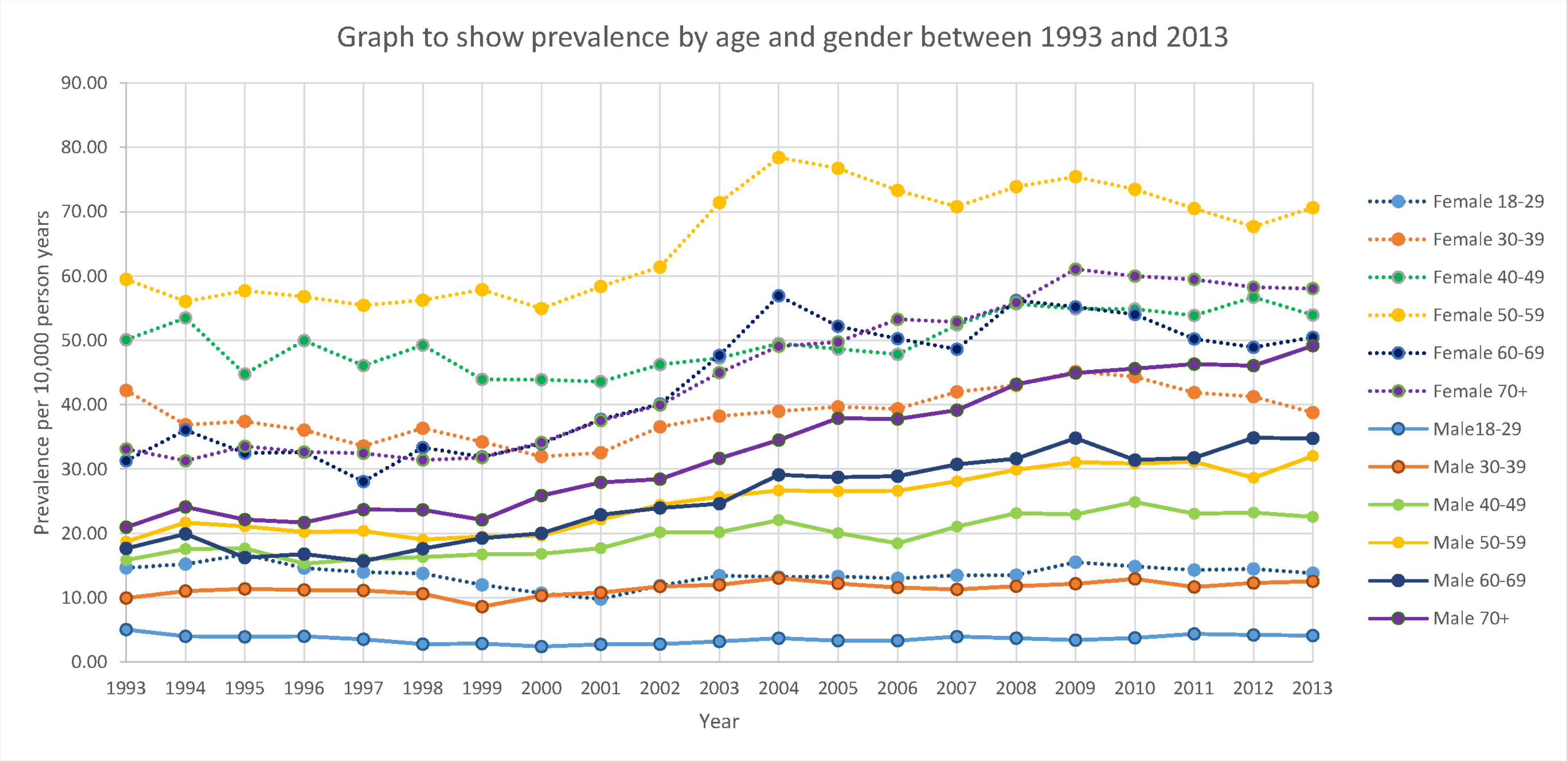
Graph to show prevalence by age and gender between 1993 and 2013
Prevalence per 10,000 person years
0.00
10.00
20.00
30.00
40.00
50.00
60.00
70.00
80.00
90.00
1993 1994 1995 1996 1997 1998 1999 2000 2001 2002 2003 2004 2005 2006 2007 2008 2009 2010 2011 2012 2013
Year
Female 18-29
Female 30-39
Female 40-49
Female 50-59
Female 60-69
Female 70+
Male18-29
Male 30-39
Male 40-49
Male 50-59
Male 60-69
Male 70+

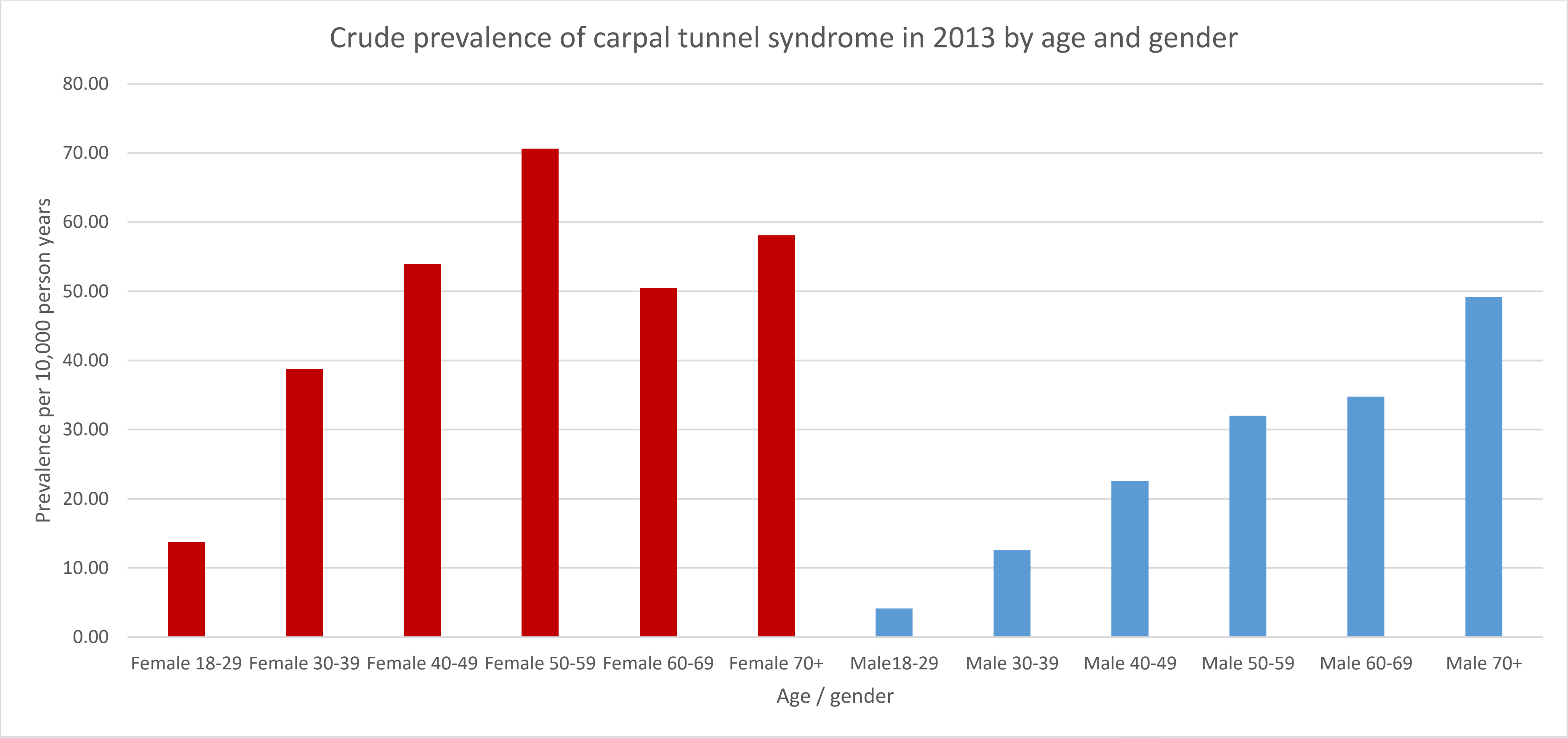

Suppl. Fig 2 Crude prevalence of carpal tunnel syndrome in 2013 by age and gender

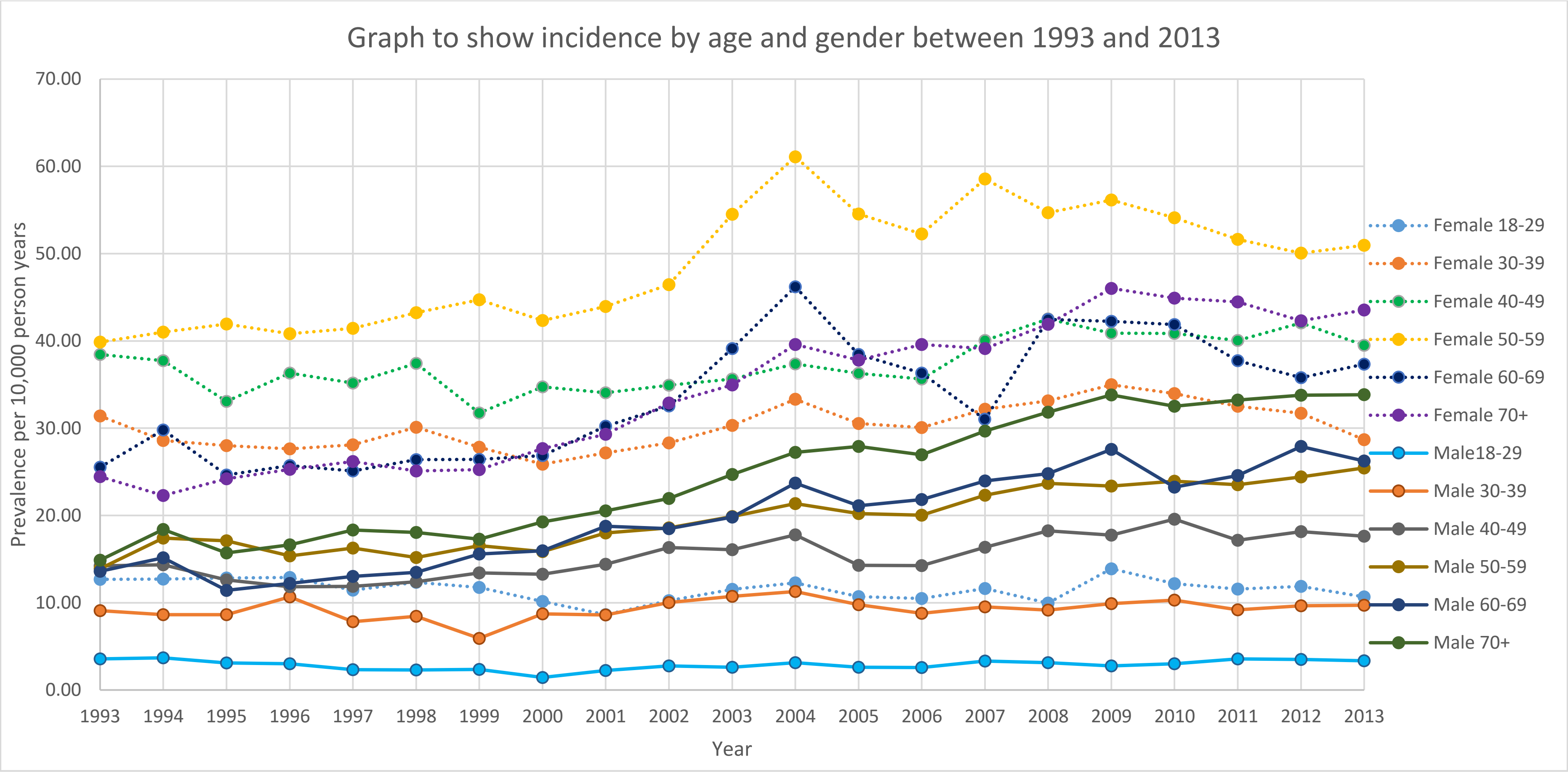

Suppl. Fig 3 Graph to show incidence by age and gender between 1993 and 2013

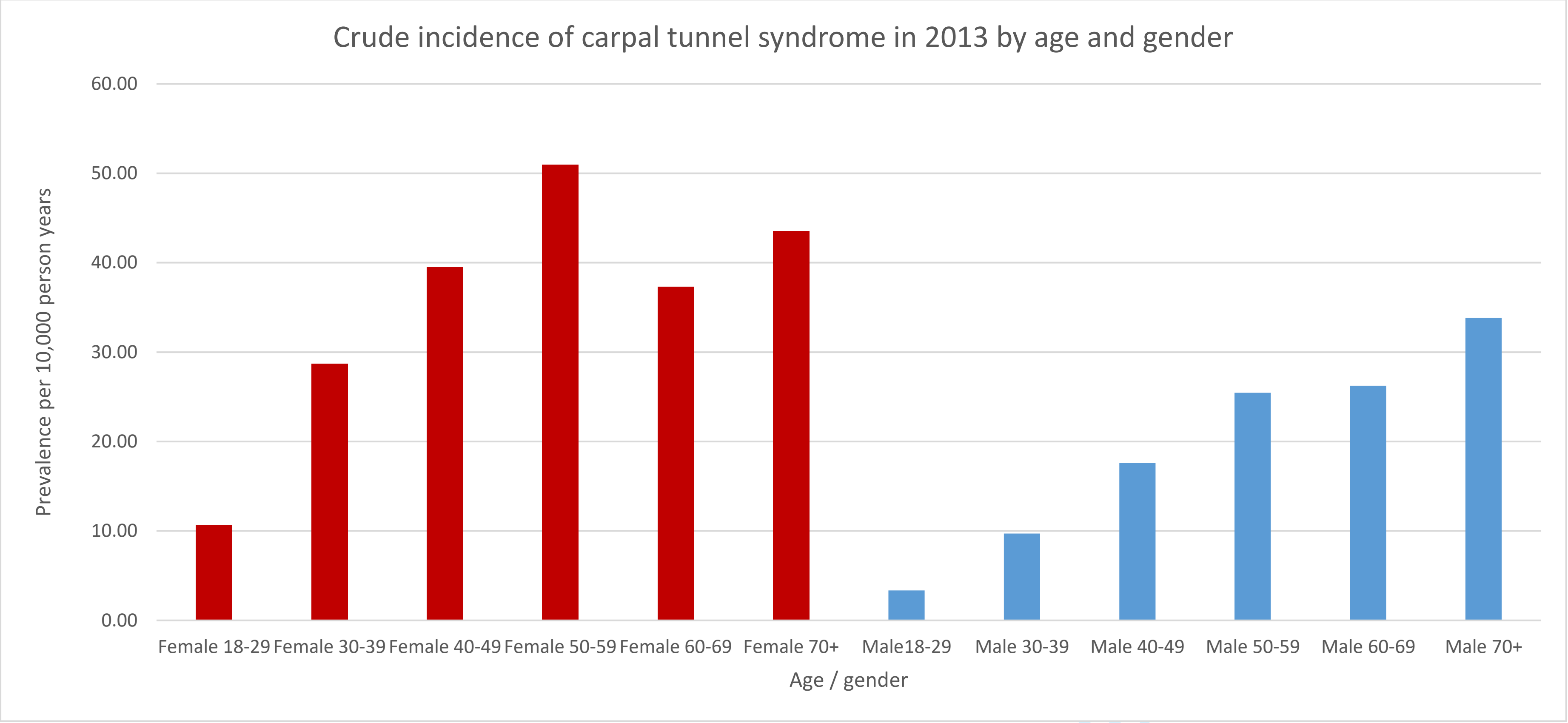

Suppl. Fig 4 Crude incidence of carpal tunnel syndrome in 2013 by age and gender

Suppl. Table 1 The age and sex standardised estimates of the annual prevalence and incidence of CTS

Year	Age sex standardised prevalence (per 10,000 person years, 95% CI)	Age sex standardised incidence (per 10,000 person years, 95% CI)
1993	26.27 (26.13 – 26.42)	19.95 (19.83 – 20.07)
1994	26.83 (26.69 – 26.98)	20.46 (20.34 – 20.59)
1995	25.90 (25.77 – 26.05)	19.20 (19.08 – 19.33)
1996	25.64 (25.50 – 25.78)	19.61 (19.49 – 19.74)
1997	24.64 (24.20 – 25.07)	19.42 (19.30 – 19.55)
1998	25.42 (25.88 – 25.56)	20.05 (19.93 – 20.18)
1999	24.57 (24.44 – 24.71)	19.51 (19.39 – 19.64)
2000	24.77 (24.63 – 24.91)	19.73 (19.61 – 19.86)
2001	26.22 (26.08 – 26.36)	20.75 (20.63 – 20.88)
2002	28.22 (28.07 – 28.37)	22.22 (22.10 – 22.36)
2003	30.81 (30.65 – 30.96)	24.28 (24.15 – 24.42)
2004	33.51 (33.35 – 33.67)	27.00 (26.86 – 27.14)
2005	32.98 (32.82 – 33.14)	24.56 (24.42 – 24.70)
2006	32.55 (32.39 – 32.70)	24.14 (24.00 – 24.27)
2007	33.48 (33.32 – 33.64)	25.52 (25.38 – 25.66)
2008	35.59 (35.43 – 25.76)	27.07 (26.92 – 27.21)
2009	36.81 (36.64 – 36.98)	28.19 (28.05 – 28.34)
2010	36.40 (36.24 – 36.66)	27.53 (27.39 – 27.68)
2011	35.28 (35.12 – 35.44)	26.59 (26.45 – 26.74)
2012	35.50 (35.34 – 35.67)	26.75 (26.61 – 26.89)
2013	35.45 (35.29 – 35.61)	26.34 (26.01 – 26.49)

For peer review only

Suppl. Table 2. Demographics of the crude prevalent population presenting with CTS in each calendar year

Year	Female median age (25% - 75% Interquartile range)	Male median age (25% - 75% Interquartile range)
**1993**	49 (38 – 62)	53 (42 – 66)
**1994**	49 (39 – 62)	53 (42 – 66)
**1995**	50 (39 – 62)	52 (41 – 64)
**1996**	50 (40 – 62)	53 (41 – 66)
**1997**	51 (40 – 62)	53 (42 – 67)
**1998**	51 (40 – 62)	54 (43 – 67)
**1999**	51 (40 – 62)	54 (44 – 66)
**2000**	52 (41 – 64)	55 (44 – 67)
**2001**	53 (42 – 65)	55 (44 – 68)
**2002**	53 (41 – 64)	55 (44 – 67)
**2003**	54 (42 – 65)	55 (44 – 68)
**2004**	55 (43 – 65)	56 (45 – 68)
**2005**	54 (43 – 65)	58 (45 – 70)
**2006**	54 (43 – 66)	58 (45 – 70)
**2007**	54 (42 – 66)	54 (42 – 66)
**2008**	54 (43 – 66)	58 (46 – 70)
**2009**	54 (43 – 67)	58 (47 – 70)
**2010**	54 (43 – 67)	57 (46 – 71)
**2011**	54 (43 – 67)	58 (47 – 71)
**2012**	54 (43 – 67)	59 (48 – 71)

For peer review only

Suppl. Table 3. Demographics of the crude incident population presenting with CTS in each calendar year

Year	Female median age (25% - 75% Interquartile range)	Male median age (25% - 75% Interquartile range)
1993	50 (39 – 63)	51 (42 – 65)
1994	50 (40 – 63)	53 (43 – 66)
1995	51 (40 – 63)	53 (42 – 64)
1996	51 (40 – 64)	52 (41 – 65)
1997	51 (40 – 64)	55 (45 – 67)
1998	51 (40 – 63)	54 (44 – 68)
1999	52 (41 – 64)	55 (45 – 67)
2000	53 (42 – 65)	55 (44 – 68)
2001	53 (42 – 66)	55 (45 – 68)
2002	54 (42 – 66)	55 (44 – 67)
2003	55 (43 – 66)	56 (45 – 68)
2004	55 (44 – 66)	57 (45 – 68)
2005	55 (43 – 66)	58 (46 – 70)
2006	55 (44 – 67)	58 (46 – 70)
2007	54 (43 – 66)	58 (47 – 70)
2008	55 (44 – 67)	58 (47 – 70)
2009	55 (44 – 67)	59 (47 – 71)

For peer review only

Suppl. Table 4 The crude prevalence of CTS by age and gender

Prevalence by age and gender	1993	1994	1995	1996	1997	1998	1999	2000	2001	2002	2003	2004	2005	2006	2007	2008	2009	2010	2011	2012	2013
Female 18-29	14.66	15.22	16.72	14.60	13.97	13.74	11.98	10.71	9.78	11.87	13.44	13.23	13.28	13.02	13.46	13.49	15.54	14.84	14.30	14.48	13.79
Female 30-39	42.21	36.87	37.37	36.04	33.57	36.34	34.20	31.94	32.55	36.57	38.21	38.97	39.67	39.34	41.98	42.99	45.15	44.34	41.87	41.24	38.78
Female 40-49	50.08	53.46	44.75	49.95	46.04	49.25	43.91	43.88	43.57	46.22	47.23	49.51	48.66	47.80	52.37	55.67	54.90	54.84	53.87	56.71	53.94
Female 50-59	59.46	56.02	57.71	56.78	55.46	56.23	57.87	54.94	58.40	61.41	71.39	78.41	76.71	73.29	70.77	73.87	75.44	73.46	70.48	67.67	70.60
Female 60-69	31.26	36.03	32.47	32.60	28.06	33.34	31.91	33.92	37.71	40.10	47.64	56.92	52.16	50.23	48.61	56.21	55.19	54.01	50.21	48.92	50.48
Female 70+	33.13	31.28	33.53	32.65	32.44	31.40	31.76	34.13	37.52	39.92	44.97	49.08	49.73	53.28	52.85	55.85	61.06	59.96	59.47	58.25	58.05
Male18-29	5.04	4.00	3.93	4.00	3.55	2.78	2.88	2.42	2.74	2.80	3.22	3.69	3.34	3.31	3.95	3.70	3.41	3.76	4.36	4.21	4.12
Male 30-39	9.95	11.02	11.36	11.19	11.11	10.60	8.61	10.32	10.78	11.75	12.00	13.06	12.23	11.57	11.30	11.78	12.17	12.94	11.67	12.29	12.55
Male 40-49	15.85	17.59	17.65	15.30	16.00	16.33	16.73	16.81	17.71	20.15	20.18	22.04	20.02	18.45	21.06	23.14	22.93	24.87	23.06	23.21	22.58
Male 50-59	18.72	21.66	21.08	20.23	20.36	19.03	19.51	19.64	22.12	24.46	25.71	26.68	26.55	26.62	28.09	29.90	31.06	30.86	31.16	28.64	32.01
Male 60-69	17.64	19.92	16.22	16.78	15.68	17.62	19.27	19.99	22.91	23.93	24.61	29.08	28.71	28.87	30.73	31.59	34.81	31.41	31.71	34.84	34.75
Male 70+	20.95	24.12	22.16	21.69	23.71	23.65	22.11	25.86	27.93	28.43	31.65	34.51	37.91	37.76	39.16	43.19	44.93	45.60	46.33	46.05	49.14

only

For peer review only - http://bmjopen.bmj.com/site/about/guidelines.xhtml

Suppl. Table 5 The crude incidence of CTS by age and gender

Incidence by age and gender	1993	1994	1995	1996	1997	1998	1999	2000	2001	2002	2003	2004	2005	2006	2007	2008	2009	2010	2011	2012	2013
Female 18-29	12.69	12.69	12.84	12.88	11.45	12.32	11.77	10.14	8.63	10.23	11.54	12.26	10.70	10.48	11.61	9.96	13.90	12.19	11.55	11.89	10.68
Female 30-39	31.40	28.58	28.00	27.62	28.09	30.09	27.83	25.88	27.18	28.31	30.31	33.31	30.53	30.06	32.17	33.13	34.97	33.96	32.51	31.72	28.69
Female 40-49	38.43	37.75	33.08	36.31	35.17	37.42	31.74	34.72	34.05	34.94	35.63	37.37	36.27	35.62	40.06	42.51	40.89	40.86	40.05	42.06	39.50
Female 50-59	39.86	41.02	41.93	40.82	41.44	43.24	44.70	42.33	43.94	46.44	54.52	61.11	54.56	52.25	58.55	54.70	56.14	54.10	51.62	50.07	50.97
Female 60-69	25.54	29.79	24.64	25.70	25.08	26.41	26.43	26.86	30.23	32.54	39.12	46.20	38.44	36.30	31.00	42.47	42.24	41.87	37.74	35.80	37.32
Female 70+	24.45	22.29	24.21	25.28	26.17	25.09	25.25	27.67	29.29	32.87	34.96	39.57	37.78	39.60	39.13	41.89	46.03	44.89	44.46	42.29	43.53
Male18-29	3.58	3.69	3.09	3.02	2.32	2.29	2.35	1.44	2.24	2.75	2.60	3.14	2.60	2.58	3.33	3.13	2.77	3.00	3.56	3.51	3.35
Male 30-39	9.09	8.63	8.63	10.67	7.82	8.45	5.92	8.74	8.60	10.01	10.72	11.28	9.76	8.80	9.53	9.16	9.90	10.30	9.19	9.67	9.72
Male 40-49	14.23	14.35	12.63	11.83	11.86	12.41	13.43	13.26	14.39	16.32	16.08	17.78	14.28	14.25	16.36	18.23	17.76	19.58	17.15	18.13	17.63
Male 50-59	13.90	17.40	17.11	15.36	16.26	15.17	16.54	15.87	18.00	18.59	19.87	21.36	20.23	20.02	22.30	23.69	23.38	23.91	23.51	24.40	25.45
Male 60-69	13.62	15.14	11.42	12.22	13.02	13.48	15.58	15.96	18.78	18.49	19.80	23.71	21.12	21.81	23.95	24.79	27.56	23.24	24.59	27.90	26.23

For peer review only

Male 70+	14.88	18.38	15.72	16.64	18.34	18.06	17.29	19.27	20.53	21.95	24.71	27.24	27.92	26.94	29.67	31.84	33.81	32.52	33.23	33.78	33.83

For peer review only

Suppl. Table 6 Comparison of population studies reporting the prevalence and / or incidence of carpal tunnel syndrome

Study Identifier	Study method	Definition of CTS	Comments
De Krom et al. 1992	Survey of a random age sex stratified sample of the general population taken from the population register of Maastricht between 1983 and 1985	Questionnaire based on symptoms and signs	[1]
Ferry et al. 1998	i) Cross sectional survey to estimate the point prevalence of hand symptoms (from a random sample of 1000 individuals from the UK general population, aged 18 to 75 years) and<br>ii) nerve conduction testing of a weighted sample<br>- Circa. 1998 (not stated)<br>- point prevalence determined	Based on nerve conduction studies using defined cut offs	Subjects over 54yrs had a higher prevalence than younger participants. No difference between genders was noted.[2]
Nordstrom et al. 1998	Prospective study conducted in the general population of the Marshfield Epidemiologic Study Area, Wisconsin, between 1991 and 1993	1. any diagnosis of possible, probable or definite CTS;<br>2. any diagnosis of probable or definite CTS; and<br>3. any diagnosis of possible , probable or definite CTS plus at least one of six clinical signs	A 3.5 fold increase in CTS incidence was noted compared with data from 20 years previously in the same study population[3]
Atroshi et al. 2000	Survey of a random sample of the age sex stratified general population of Southern Sweden, in 1997	Diagnosis based on clinical examination and positive electrophysiological findings	The population prevalence of symptoms was 14.4%; the prevalence of clinically and electrophysiologically confirmed CTS was 2.7% [4]
Papanicolaou, McCable & Firrell 2001	Cross-sectional study to evaluate prevalence of carpal tunnel syndrome in the General population of the United States	Katz hand diagram	After correcting for nonresponders the lowest possible estimate of CTS was 3.72% [5]

For peer review only

Mondelli, Giannini & Giacchi 2002	Prospective study of patients referred to four electrodiagnostic laboratories in the Siena area, Italy. Mean annual incidence calculated from time period 1991 to 1998	Diagnosis based on clinical history and electrodiagnostic evidence of a reduced distal conduction velocity of the median nerve (American Academy of Neurology standards)	Of the patients presenting 79.7% were women. The mean age at diagnosis was 55.0 +/- 14.4 years (range 16 to 97) [6]
Bland, Rudolfer 2003	Prospective collection of neurophysiological and clinical data of patients referred to two electromyography clinics in the UK between 1991 to 1993 and 1992 to 2001	Based on nerve conduction studies using defined cut offs	An increase in diagnosed cases was observed between the two data collection periods; attributed to referral of milder cases. Median nerve impairment was more severe in the elderly and men at all ages. [7]
Latinovic, Gulliford & Hughes 2006	Population study based in a general practice database of consulting primary care patients from 253 practices between January 1992 and 31 December 2000.	Read and Oxmis codes for carpal tunnel syndrome	Most frequent in women aged 45-54. In 2000 operative treatment was undertaken for 31% of incident CTS presentations [8]
Bonger et al. 2007	Analysis of the first and second Dutch National Survey of General Practice, conducted in 1987 and 2001	(International Classification of Primary Care) ICPC coded diagnosis	A crude increase in incidence over time was not statistically significant after subdividing by age and sex. Incidence rates were related to the job level in women, but not men [9]
Dieleman et al. 2008	Population study based in a general practice database (Integrated Primary Care Information (IPCI) database): data of consulting primary care patients in the Netherlands between 1996 and 2003	ICPC coded diagnosis	Neuropathic pain was noted to affect almost 1% of the population. Mononeuropathies and carpal tunnel syndrome were the most common causes [10]
Gelfman et al. 2009	Analysis of medical records linkage system 1981-1985 to 2000-2005 of residents of Olmsted County, Minnesota (Rochester Epidemiology Project)	Clinical coding with a sample verified by full record review	An increase in incidence was observed over the study period. An increase in young individuals seeking care for less severe CTS in the mid-1980's was followed in the 1990's by an increasing incidence in older people [11]

For peer review only

Atroshi et al. 2011	Analysis of the Skane Health Care Register (SCHR) (inhabitants presenting to public health providers), incident cases identified between 2006 - 2008	Physician diagnosed	[12]
Jenkins et al. 2012b	Prospective audit of patients referred to a regional hand service based in secondary care in Scotland between November 2004 and May 2010	Symptoms of pain or paraesthesia in the median nerve distribution and one or more of: nerve conduction deficit, thenar muscle wasting or positive Tinel or Phalen signs	Mean age of presentation 55.1years (range 22 to 96, SD 13.5 years). Mean body mass index at presentation 29.5 kg/m2 CTS more common in: females (OR 1.9, 95% CI 1.5 to 2.5) Incidence varied significantly between deprivation groups: most deprived 81/100,000 and least deprived 62/100,000 (OR 1.3, 95% CI 1.1 to 1.6) [13]
Jenkins et al. 2013	Prospective audit of patients referred to a regional hand service based in secondary care in Scotland between November 2004 and May 2010, who were employed	Clinical diagnosis based on history and examination, in most cases substantiated by nerve conduction studies	The greatest incidence as in caring and leisure occupations (197 per 100,000) and the lowest incidence was in the associate professional group (37 per 100,000) [14]
Dale 2013	Pooled analysis of six prospective studies collecting data from >50 workplaces, over variable time frames	A pooled case definition was derived to include clinical and elctrodiagnostic criteria	7.8% of 4321 subjects studied had prevalent CTS, with an additional 204 subjects meeting the CTS criteria, leading to an incidence of 2.3 cases per 100 person years [15]

For peer review only

1 de Krom MC, Knipschild PG, Kester AD, et al. Carpal tunnel syndrome: prevalence in the general population. *J Clin Epidemiol* 1992;45:373-6.

2 Ferry S, Pritchard T, Keenan J, et al. Estimating the prevalence of delayed median nerve conduction in the general population. *Br J Rheumatol* 1998;37:630-5.

3 Nordstrom DL, DeStefano F, Vierkant RA, et al. Incidence of diagnosed carpal tunnel syndrome in a general population. *Epidemiology* 1998;9:342-5.

4 Atroshi I. Carpal tunnel syndrome--prevalence, electrodiagnosis and outcome instruments. *Acta Orthop Scand* 1999;70:70-1.

5 Papanicolaou GD, McCabe SJ, Firrell J. The prevalence and characteristics of nerve compression symptoms in the general population. *Journal of Hand Surgery - American Volume* 2001;26:460-6.

6 Mondelli M, Giannini F, Giacchi M. Carpal tunnel syndrome incidence in a general population. *Neurology* 2002;58:289-94.

7 Bland JDP, Rudolfer SM. Clinical surveillance of carpal tunnel syndrome in two areas of the United Kingdom, 1991–2001. *Journal of Neurology, Neurosurgery & Psychiatry* 2003;74:1674-9 doi:10.1136/jnnp.74.12.1674.

8 Latinovic R, Gulliford MC, Hughes RA. Incidence of common compressive neuropathies in primary care. *J Neurol Neurosurg Psychiatry* 2006;77:263-5 doi:10.1136/jnnp.2005.066696.

9 Bongers FJ, Schellevis FG, van den Bosch WJ, et al. Carpal tunnel syndrome in general practice (1987 and 2001): incidence and the role of occupational and non-occupational factors. *Br J Gen Pract* 2007;57:36-9.

10 Dieleman JP, Kerklaan J, Huygen FJ, et al. Incidence rates and treatment of neuropathic pain conditions in the general population. *Pain* 2008;137:681-8 doi:10.1016/j.pain.2008.03.002 [doi].

11 Gelfman R, Melton LJ,3rd, Yawn BP, et al. Long-term trends in carpal tunnel syndrome. *Neurology* 2009;72:33-41 doi:10.1212/01.wnl.0000338533.88960.b9; 10.1212/01.wnl.0000338533.88960.b9.

12 Atroshi I, Englund M, Turkiewicz A, et al. Incidence of physician-diagnosed carpal tunnel syndrome in the general population. *Arch Intern Med* 2011;171:943-5.

13 Jenkins PJ, Watts AC, Duckworth AD, et al. Socioeconomic deprivation and the epidemiology of carpal tunnel syndrome. *Journal of Hand Surgery-European Volume* 2012;37E:123-9 doi:10.1177/1753193411419952.

14 Jenkins PJ, Srikantharajah D, Duckworth AD, et al. Carpal tunnel syndrome: the association with occupation at a population level. *Journal of Hand Surgery-European Volume* 2013;38E:67-72 doi:10.1177/1753193412455790.

15 Dale AM, Harris-Adamson C, Rempel D, et al. Prevalence and incidence of carpal tunnel syndrome in US working populations: Pooled analysis of six prospective studies. *Scandinavian Journal of Work, Environment and Health* 2013;39:495-505.

For peer review only

For peer review only

Suppl. Table 7 summary of reported prevalence and incidence by gender

Study Identifier	Country of Origin Data collection (Prevalence or Incidence)	Prevalence or Incidence per 100,000, per annum			Female / male ratio
		All	Female	Male	
De Krom et al. 1992	The Netherlands 1983 - July 1985 (Prevalence)	5700	5800	600	9.66
Atroshi et al. 2000	Sweden 1997 (Prevalence)	3800	4600	2800	1.64
Papanicolaou, McCable & Firrell 2001	United States 2001 (Prevalence)	3720			4.8
Ferry et al. 1998	United Kingdom Not stated (Incidence)	8200	6400	8200	0.78
Nordstrom et al. 1998	United States 1991 - 1993 (Incidence)	346	373	318	1.17
Mondelli, Giannini & Giacchi 2002	Italy 1991 – 1998 (mean) (Incidence)	276	506	139	3.64
Bland, Rudolfer 2003	Kent, UK 1991 - 2001 (Incidence)	105	120.5	60	2
	Huddersfield, UK		61.5	30	2

For peer review only

Latinovic, Gulliford & Hughes 2006	United Kingdom (Incidence)		192.8	87.8	2.23
Bongers et al. 2007	The Netherlands (Incidence) 1987	130	190	60	3.17
	2001	180	280	90	3.11
Dieleman et al. 2008	The Netherlands 1996 - 2003 (Incidence)	233.1			
Gelfman et al. 2009	United States (Incidence) 1981-1985	258	337	177	1.90
	2001-2005	424	542	303	1.79
Atroshi et al. 2011	Sweden 2006 - 2008 (Incidence)		428	182	2.35
Jenkins et al. 2012b	Scotland 2004 - 2010 (Incidence)	72	98	43	2.28
Jenkins et al. 2013	Scotland 2004 - 2010 (Incidence)	103			
Dale 2013	United States (Incidence)	2300			

For peer review only

STROBE Statement—checklist of items that should be included in reports of observational studies

	Item No	Recommendation	Page
**Title and abstract**	1	(*a*) Indicate the study's design with a commonly used term in the title or the abstract	1-2
		(*b*) Provide in the abstract an informative and balanced summary of what was done and what was found	1-2
**Introduction**			
Background/rationale	2	Explain the scientific background and rationale for the investigation being reported	4
Objectives	3	State specific objectives, including any prespecified hypotheses	4-5
**Methods**			
Study design	4	Present key elements of study design early in the paper	5
Setting	5	Describe the setting, locations, and relevant dates, including periods of recruitment, exposure, follow-up, and data collection	5-6
Participants	6	(*a*) *Cohort study*—Give the eligibility criteria, and the sources and methods of selection of participants. Describe methods of follow-up<br>*Case-control study*—Give the eligibility criteria, and the sources and methods of case ascertainment and control selection. Give the rationale for the choice of cases and controls<br>*Cross-sectional study*—Give the eligibility criteria, and the sources and methods of selection of participants	5-6
		(*b*) *Cohort study*—For matched studies, give matching criteria and number of exposed and unexposed<br>*Case-control study*—For matched studies, give matching criteria and the number of controls per case	
Variables	7	Clearly define all outcomes, exposures, predictors, potential confounders, and effect modifiers. Give diagnostic criteria, if applicable	5-6
Data sources/ measurement	8*	For each variable of interest, give sources of data and details of methods of assessment (measurement). Describe comparability of assessment methods if there is more than one group	na
Bias	9	Describe any efforts to address potential sources of bias	na
Study size	10	Explain how the study size was arrived at	na
Quantitative variables	11	Explain how quantitative variables were handled in the analyses. If applicable, describe which groupings were chosen and why	6-7
Statistical methods	12	(*a*) Describe all statistical methods, including those used to control for confounding	6-7
		(*b*) Describe any methods used to examine subgroups and interactions	na
		(*c*) Explain how missing data were addressed	na
		(*d*) *Cohort study*—If applicable, explain how loss to follow-up was addressed<br>*Case-control study*—If applicable, explain how matching of cases and controls was addressed<br>*Cross-sectional study*—If applicable, describe analytical methods taking account of sampling strategy	na
		(*e*) Describe any sensitivity analyses	6-7

For peer review only - http://bmjopen.bmj.com/site/about/guidelines.xhtml

Continued on next page.

**Results**			
Participants	13*	(a) Report numbers of individuals at each stage of study—eg numbers potentially eligible, examined for eligibility, confirmed eligible, included in the study, completing follow-up, and analysed	Na
		(b) Give reasons for non-participation at each stage	Na
		(c) Consider use of a flow diagram	Na
-Descriptive data	14*	(a) Give characteristics of study participants (eg demographic, clinical, social) and information on exposures and potential confounders	7-9
		(b) Indicate number of participants with missing data for each variable of interest	na
		(c) *Cohort study*—Summarise follow-up time (eg, average and total amount)	na
Outcome data	15*	*Cohort study*—Report numbers of outcome events or summary measures over time	
		Case-control study—Report numbers in each exposure category, or summary measures of exposure	
		Cross-sectional study—Report numbers of outcome events or summary measures	7-9
Main results	16	(*a*) Give unadjusted estimates and, if applicable, confounder-adjusted estimates and their precision (eg, 95% confidence interval). Make clear which confounders were adjusted for and why they were included	7-9+ tables
		(*b*) Report category boundaries when continuous variables were categorized	na
		(*c*) If relevant, consider translating estimates of relative risk into absolute risk for a meaningful time period	na
Other analyses	17	Report other analyses done—eg analyses of subgroups and interactions, and sensitivity analyses	na
**Discussion**			
Key results	18	Summarise key results with reference to study objectives	12
Limitations	19	Discuss limitations of the study, taking into account sources of potential bias or imprecision. Discuss both direction and magnitude of any potential bias	14
Interpretation	20	Give a cautious overall interpretation of results considering objectives, limitations, multiplicity of analyses, results from similar studies, and other relevant evidence	12-15
Generalisability	21	Discuss the generalisability (external validity) of the study results	14
**Other information**			
Funding	22	Give the source of funding and the role of the funders for the present study and, if applicable, for the original study on which the present article is based	15

*Give information separately for cases and controls in case-control studies and, if applicable, for exposed and unexposed groups in cohort and cross-sectional studies.

Note: An Explanation and Elaboration article discusses each checklist item and gives methodological background and published examples of transparent reporting. The STROBE checklist is best used in conjunction with this article (freely available on the Web sites of PLoS Medicine at http://www.plosmedicine.org/, Annals of Internal Medicine at http://www.annals.org/, and Epidemiology at http://www.epidem.com/). Information on the STROBE Initiative is available at www.strobe-statement.org.

For peer review only

For peer review only - http://bmjopen.bmj.com/site/about/guidelines.xhtml
